# Supplementary material for: Estimating health spending associated with chronic multimorbidity in 2018: An observational study among adults in the United States
Source: PLoS Med. 2023 Apr 4;20(4):e1004205. doi: 10.1371/journal.pmed.1004205 (PMC10072449; doi:10.1371/journal.pmed.1004205)
Supplement: S1 Appendix — Table A1. List of most-detailed GBD causes determined as chronic conditions. Table A2. Chronic conditions and its most-detailed GBD causes. Table A3. Summary statistics of 50 SGD model runs. Table A4. Summary statistics of study population. Table A5. Covariates and their regression coefficients. Table A6. Observed prevalence and estimated multimorbidity-adjusted annual spending per treated case for the “other” conditions. Table A7. Regression coefficients for disease combinations with prevalence rate of greater than 100 per 100,000 (ordered by spending). (DOCX) [file pmed.1004205.s002.docx]

**Estimating health spending associated with chronic multimorbidity in 2018: an observational study among adults in the United States**

Angela Y. Chang, Dana Bryazka, Joseph L. Dieleman

Appendix

**Table of content**

1. Selection of chronic conditions
2. Distributing spending associated with disease combinations to individual diseases
3. Stochastic gradient descent model parameter selection and summary statistics of the model runs
4. Detailed results figures and tables
5. **Selection of chronic conditions**
6. **List of most-detailed Global Burden of Disease causes identified as chronic conditions**

Medical experts from the Institute for Health Metric and Evaluation screened and selected a subset of conditions they consider as “chronic” among all 293 Global Burden of Disease **(**GBD) causes, listed in Table A1. Injuries were excluded from this analysis.

Table A1. List of most-detailed Global Burden of Disease causes determined as chronic conditions

| HIV/AIDS resulting in other diseases | Non-Hodgkin lymphoma | Interstitial lung disease and pulmonary sarcoidosis | Rheumatoid arthritis | Edentulism and severe tooth loss |
| --- | --- | --- | --- | --- |
| HIV/AIDS - Drug-susceptible Tuberculosis | Multiple myeloma | Other chronic respiratory diseases | Osteoarthritis | Atopic dermatitis |
| HIV/AIDS - Multidrug-resistant Tuberculosis without extensive drug resistance | Acute lymphoid leukemia | Cirrhosis and other chronic liver diseases due to hepatitis B | Low back pain | Contact dermatitis |
| HIV/AIDS - Extensively drug-resistant Tuberculosis | Chronic lymphoid leukemia | Cirrhosis and other chronic liver diseases due to hepatitis C | Neck pain | Seborrhoeic dermatitis |
| Esophageal cancer | Acute myeloid leukemia | Cirrhosis and other chronic liver diseases due to alcohol use | Gout | Psoriasis |
| Stomach cancer | Chronic myeloid leukemia | Cirrhosis and other chronic liver diseases due to other causes | Other musculoskeletal disorders | Acne vulgaris |
| Liver cancer due to hepatitis B | Other leukemia | Cirrhosis due to NASH | Benign prostatic hyperplasia | Alopecia areata |
| Liver cancer due to hepatitis C | Other malignant neoplasms | Inguinal, femoral, and abdominal hernia | Male infertility | Pruritus |
| Liver cancer due to alcohol use | Myelodysplastic, myeloproliferative, and other hematopoietic neoplasms | Inflammatory bowel disease | Uterine fibroids | Urticaria |
| Liver cancer due to other causes | Benign and in situ intestinal neoplasms | Vascular intestinal disorders | Polycystic ovarian syndrome | Decubitus ulcer |
| Liver cancer due to NASH | Benign and in situ cervical and uterine neoplasms | Gallbladder and biliary diseases | Female infertility | Age-related and other hearing loss |
| Larynx cancer | Other benign and in situ neoplasms | Peptic ulcer disease | Endometriosis | Near vision loss |
| Tracheal, bronchus, and lung cancer | Rheumatic heart disease | Gastritis and duodenitis | Genital prolapse | Glaucoma |
| Breast cancer | Ischemic heart disease | Gastroesophageal reflux disease | Premenstrual syndrome | Cataract |
| Cervical cancer | Ischemic stroke | Alzheimer's disease and other dementias | Thalassemias | Age-related macular degeneration |
| Uterine cancer | Intracerebral hemorrhage | Parkinson's disease | Sickle cell disorders | Other vision loss |
| Prostate cancer | Subarachnoid hemorrhage | Epilepsy | G6PD deficiency | Refraction disorders |
| Colon and rectum cancer | Hypertensive heart disease | Multiple sclerosis | Other hemoglobinopathies and hemolytic anemias | Alcohol use disorders |
| Lip and oral cavity cancer | Alcoholic cardiomyopathy | Motor neuron disease | Thalassemias trait | Opioid use disorders |
| Nasopharynx cancer | Myocarditis | Other neurological disorders | Sickle cell trait | Cocaine use disorders |
| Other pharynx cancer | Other cardiomyopathy | Migraine | G6PD trait | Amphetamine use disorders |
| Gallbladder and biliary tract cancer | Atrial fibrillation and flutter | Tension-type headache | Endocrine, metabolic, blood, and immune disorders | Cannabis use disorders |
| Pancreatic cancer | Peripheral artery disease | Schizophrenia | Neural tube defects | Other drug use disorders |
| Malignant skin melanoma | Endocarditis | Major depressive disorder | Congenital heart anomalies | Diabetes mellitus type 1 |
| Non-melanoma skin cancer (squamous-cell carcinoma) | Non-rheumatic calcific aortic valve disease | Dysthymia | Orofacial clefts | Diabetes mellitus type 2 |
| Non-melanoma skin cancer (basal-cell carcinoma) | Non-rheumatic degenerative mitral valve disease | Bipolar disorder | Down syndrome | Chronic kidney disease due to hypertension |
| Ovarian cancer | Other non-rheumatic valve diseases | Anxiety disorders | Turner syndrome | Chronic kidney disease due to glomerulonephritis |
| Testicular cancer | Other cardiovascular and circulatory diseases | Anorexia nervosa | Klinefelter syndrome | Chronic kidney disease due to other and unspecified causes |
| Kidney cancer | Chronic obstructive pulmonary disease | Bulimia nervosa | Other chromosomal abnormalities | Chronic kidney disease due to diabetes mellitus type 1 |
| Bladder cancer | Silicosis | Autism spectrum disorders | Congenital musculoskeletal and limb anomalies | Chronic kidney disease due to diabetes mellitus type 2 |
| Brain and nervous system cancer | Asbestosis | Attention-deficit/hyperactivity disorder | Urogenital congenital anomalies |  |
| Thyroid cancer | Coal workers pneumoconiosis | Conduct disorder | Digestive congenital anomalies |  |
| Mesothelioma | Other pneumoconiosis | Idiopathic developmental intellectual disability | Other congenital birth defects |  |
| Hodgkin lymphoma | Asthma | Other mental disorders | Periodontal diseases |  |

1. **List of 63 chronic conditions included in the study and the most-detailed Global Burden of Disease causes included under this condition**

166 most-detailed GBD causes were determined as chronic, of which we reduced down to 63 by combining them into larger disease categories to improve data efficiency. Table A2 summarizes how these causes were combined to a larger cause category.

Table A2. Chronic conditions and its most-detailed Global Burden of Disease (GBD) causes

| **Chronic condition included in this study** | **Most-detailed GBD causes included under this condition** |
| --- | --- |
| *Neoplasms* | |
| Bladder and kidney cancers | Bladder cancer  Kidney cancer |
| Blood cancers | Leukemia: acute lymphoid leukemia, chronic lymphoid leukemia, acute myeloid leukemia, chronic myeloid leukemia, other leukemia  Multiple myeloma  Non-Hodgkin lymphoma |
| Brain and nervous system cancer | Brain and nervous system cancer |
| Breast cancer | Breast cancer |
| Colon and rectum cancer | Colon and rectum cancer |
| Ear, nose, throat cancers | Larynx cancer  Lip and oral cavity cancer  Nasopharynx cancer  Other pharynx cancer |
| Reproductive organ cancers | Cervical cancer  Ovarian cancer  Uterine cancer  Prostate cancer  Testicular cancer |
| Gastrointestinal gland cancers | Liver cancer: liver cancer due to hepatitis B, hepatitis C, alcohol use, NASH, and other causes  Gallbladder and biliary tract cancer  Pancreatic cancer |
| Hodgkin lymphoma | Hodgkin lymphoma |
| Lip and oral cavity cancers | Lip and oral cavity cancers |
| Skin cancers | Malignant skin melanoma  Non-melanoma skin cancer: squamous-cell carcinoma, basal-cell carcinoma |
| Thyroid cancer | Thyroid cancer |
| Tracheal, bronchus, and lung cancer | Tracheal, bronchus, and lung cancer |
| Upper gastrointestinal cancers | Esophageal cancer  Stomach cancer |
| Other neoplasms | Benign neoplasms  Mesothelioma  Myelodysplatic, myeloproliferative, and other hematopoietic neoplasms  Other malignant neoplasms |
| *Cardiovascular diseases* | |
| Atrial fibrillation and flutter | Atrial fibrillation and flutter |
| Ischemic heart disease-related heart conditions | Cardiomyopathy and myocarditis: myocarditis, alcoholic cardiomyopathy, other cardiomyopathy  Hypertensive heart disease  Ischemic heart disease |
| Rheumatic heart disease | Rheumatic heart disease |
| Stroke | Intracerebral hemorrhage  Ischemic stroke  Subarachnoid hemorrhage |
| Peripheral vascular disease | Peripheral artery disease  Peripheral vascular disease |
| Other cardiovascular and circulatory diseases | Endocarditis  Non-rheumatic valvular heart disease  Other cardiovascular and circulatory diseases |
| *Chronic respiratory diseases* | |
| Asthma | Asthma |
| Chronic obstructive pulmonary disease | Chronic obstructive pulmonary disease |
| Interstitial lung disease and pulmonary sarcoidosis | Interstitial lung disease and pulmonary sarcoidosis |
| Other chronic respiratory diseases | Pneumoconiosis: silicosis, asbestosis, coal workers pneumoconiosis  Other chronic respiratory diseases |
| *Digestive diseases* | |
| Cirrhosis | Cirrhosis and other chronic liver diseases due to hepatitis B, hepatitis C, alcohol use, NASH, and other causes |
| Inflammatory bowel disease | Inflammatory bowel disease |
| Inguinal, femoral, and abdominal hernia | Inguinal, femoral, and abdominal hernia |
| Gallbladder and biliary diseases | Gallbladder and biliary diseases |
| Gastritis and duodenitis, peptic ulcer disease | Gastritis and duodenitis  Peptic ulcer disease |
| Other digestive diseases | Vascular intestinal disorders  Other digestive diseases |
| *Neurological disorders* | |
| Alzheimer’s disease and other dementias | Alzheimer’s disease and other dementias |
| Epilepsy | Epilepsy |
| Headache disorders | Migraine  Tension-type headache |
| Multiple sclerosis | Multiple sclerosis |
| Parkinson’s disease | Parkinson’s disease |
| Other neurological disorders | Motor neuron disease  Other neurological disorders |
| *Mental disorders* | |
| Attention-deficit / hyperactivity disorder | Attention-deficit / hyperactivity disorder |
| Anxiety disorders | Anxiety disorders |
| Bipolar disorder | Bipolar disorder |
| Depressive disorders | Dysthymia  Major depressive disorder |
| Schizophrenia | Schizophrenia |
| Substance use disorders | Alcohol use disorders  Drug use disorders: opioid, cocaine, amphetamine, cannabis, and other drug use disorders |
| Other mental disorders | Autism spectrum disorders  Conduct disorder  Eating disorders: anorexia nervosa, bulimia nervosa  Idiopathic developmental intellectual disability  Other mental disorders |
| *Diabetes and kidney diseases* | |
| Chronic kidney disease | Chronic kidney disease due to diabetes mellitus type 1, diabetes mellitus type 2, hypertension, glomerulonephritis, and other unspecified causes |
| Diabetes | Diabetes mellitus type 1  Diabetes mellitus type 2 |
| *Skin and subcutaneous diseases* | |
| Skin and subcutaneous diseases | Acne vulgaris  Alopecia areata  Atopic dermatitis  Contact dermatitis  Decubitus ulcer  Pruritus  Psoriasis  Seborrheic dermatitis  Urticaria |
| *Sense organ diseases* | |
| Sense organ diseases | Blindness and vision impairment: glaucoma, cataract, age-related macular degeneration, refraction disorders, near vision loss, other vision loss  Age-related and other hearing loss |
| *Musculoskeletal disorders* | |
| Gout | Gout |
| Low back and neck pain | Low back pain  Neck pain |
| Osteoarthritis | Osteoarthritis |
| Rheumatoid arthritis | Rheumatoid arthritis |
| Other musculoskeletal disorders | Other musculoskeletal disorders |
| *Other non-communicable diseases* | |
| Congenital birth defects | Congenital heart anomalies  Congenital musculoskeletal and limb anomalies  Digestive congenital anomalies  Down syndrome  Klinefelter syndrome  Neural tube defects  Orofacial clefts  Other chromosomal abnormalities  Turner syndrome  Urogenital congenital anomalies  Other congenital birth defects |
| Gynecological diseases | Endometriosis  Female infertility  Genital prolapse  Polycystic ovarian syndrome  Premenstrual syndrome  Uterine fibroids |
| Hemoglobinopathies and hemolytic anemias | G6PD deficiency  G6PD trait  Sickle cell disorders  Sickle cell trait  Thalassemias  Thalassemias trait  Other hemoglobinopathies and hemolytic anemias |
| Endocrine, metabolic, blood, and immune disorders | Endocrine, metabolic, blood, and immune disorders |
| Oral disorders | Peridontal diseases  Edentulism and sever tooth loss |
| *Communicable diseases* | |
| HIV | HIV/AIDS - Drug-susceptible Tuberculosis  HIV/AIDS - Multidrug-resistant Tuberculosis without extensive drug resistance  HIV/AIDS - Extensively drug-resistant Tuberculosis  HIV/AIDS resulting in other disease |
| *Risk factors* | |
| Hyperlipidemia | Hyperlipidemia |
| Hypertension | Hypertension |
| Obesity | Obesity |
| Tobacco use | Tobacco use |

**C. Discussion on the exclusion of “other” categories**

For each main disease categories in the GBD hierarchy, there is a “other” sub-category that encompasses conditions that are less prevalent. Some of these “other” sub-categories contain a mix of chronic and non-chronic conditions. For simplicity, we classified some as chronic if we believed the majority of these other conditions are in fact chronic. A more accurate categorization of more detailed conditions would be desirable in theory but difficult to implement in reality due to constrains in computational power. Due to this limitation, we included these “other” conditions in the main model but only report the results in the Appendix.

**2. Distributing spending associated with disease combinations to individual diseases**

1. **Distributing spending for two- and three-way combinations**

This study includes two- and three-way combinations of diseases in the regression model. For two-way combinations, we briefly described the approach in the main manuscript and provide more detail here:

Taking the same example as the manuscript, the coefficient of the interaction term for the diabetes–osteoarthritis combination needs to be split into one part associated with diabetes and another with osteoarthritis. Here are the four steps to do so:

Step 1: we ran a linear regression model among study population with diabetes to estimate the effect of having osteoarthritis on annual health spending:

${spend}_{j}= {\beta_{0}}_{j}+ \sum_{k=1}^{K-1} \beta_{jk}{dx}_{jk} + {age}_{j}+ {sex}_{j}+ {region}_{i}+ \varepsilon_{i}$ (1)

where $j$ is the enrollee with the disease (diabetes in this example), ${dx}_{k}$ is each additional chronic condition beyond diabetes. $\beta_{osteoarthritis | diabetes}$ is the coefficient representing the effect of having osteoarthritis on spending among people with diabetes. We run the same model for people with osteoarthritis to derive $\beta_{diabetes | osteoarthritis}$, the coefficient representing the effect of having diabetes on health spending among people with osteoarthritis.

Step 2: we take the coefficient of the interaction term for diabetes and osteoarthritis, $\beta_{diabetes, osteoarthritis}$ (derived from equation 1), and split the coefficient into two parts:

$\beta_{diabetes, osteoarthritis}= \beta_{diabetes | combination}+ \beta_{osteoarthritis | combination}$ (2)

$$= \beta_{diabetes, osteoarthritis} \times\frac{\beta_{diabetes | osteoarthritis}}{\beta_{diabetes | osteoarthritis}+ \beta_{osteoarthritis | diabetes}}$$

$+ \beta_{diabetes, osteoarthritis} \times\frac{\beta_{osteoarthritis | diabetes}}{\beta_{diabetes | osteoarthritis}+ \beta_{osteoarthritis | diabetes}}$

where $\beta_{diabetes | combination}$ is the estimated part of the interaction coefficient that is attributed to diabetes, $\beta_{osteoarthritis | combination}$ is the part attributed to osteoarthritis. $\beta_{diabetes | osteoarthritis}$ is the coefficient from equation (1) on the indicator variable of whether those with osteoarthritis also have diabetes as a comorbidity, and $\beta_{osteoarthritis | diabetes}$ is the coefficient on the indicator variable of whether those with diabetes also have osteoarthritis as a comorbidity.

Step 3: we calculated the probability of the disease combinations occurring among people with diabetes (for example, the probability of someone with diabetes also having osteoarthritis).

Step 4: we multiplied each estimated spending associated with combinations of diabetes and another disease with its probability from step 3, and summed across disease combinations. This final figure is the part of the spending associated with dyads that should be attributed to diabetes.

For three-way combinations, the approach is similar to what is described above. Using diabetes, osteoarthritis, and hypertension as an example, the first step is to run the following model for all two-disease combinations:

${spend}_{i}= {\beta_{0}}_{i}+ \sum_{j=1}^{J-2} \beta_{ij}{dx}_{ij} + {age}_{i}+ {sex}_{i}+ {region}_{i}+ \varepsilon_{i}$ (3)

where $i$ represents enrollees with diabetes and osteoarthritis, and ${dx}_{j}$ represent all chronic conditions in the study except diabetes and osteoarthritis. $\beta_{hypertension}$ is the coefficient representing the effect of having hypertension on health spending among people with diabetes and osteoarthritis, and ${{\beta_{0}}_{diabetes \& osteoarthritis}+ \beta}_{hypertension}$ is the estimated spending for people with hypertension (among those with diabetes and osteoarthritis), where ${\beta_{0}}_{diabetes \& osteoarthritis}$ is the intercept term of the regression model among people with diabetes and osteoarthritis. We run the same model for people with the combination of osteoarthritis and hypertension and the combination of diabetes and hypertension, to derive $\beta_{diabetes}$ and $\beta_{osteoarthritis}$, respectively. Then,

$\beta_{diabetes, osteoarthritis, hypertension}$ (4)

$$= \beta_{diabetes | combo}+ \beta_{osteoarthritis | combo}+ \beta_{hypertension | combo}$$

$$= \beta_{diabetes, osteoarthritis, hypertension} \times\frac{\beta_{dm | osteo+htn}}{\beta_{dm | osteo+htn}+ \beta_{osteo | dm+htn}+\beta_{htn | dm+osteo}}$$

$+ \beta_{diabetes, osteoarthritis, hypertension}\times\frac{\beta_{osteo | dm+htn}}{\beta_{dm | osteo+htn}+ \beta_{osteo | dm+htn}+\beta_{htn | dm+osteo}}$

$+ \beta_{diabetes, osteoarthritis, hypertension}\times\frac{\beta_{htn | dm+osteo}}{\beta_{dm | osteo+htn}+ \beta_{osteo | dm+htn}+\beta_{htn | dm+osteo}}$

1. **Restrictions on combinations with both positive and negative coefficients**

One issue arises with the approach discussed in section A above when the coefficients (for example, $\beta_{diabetes | osteoarthritis}$ and $\beta_{osteoarthritis | diabetes}$) have different signs.

In equation (2), if $\beta_{diabetes}$ is positive and $\beta_{osteoarthritis}$ is negative, for example, the ratio $\frac{\beta_{diabetes | osteoarthritis}}{\beta_{diabetes | osteoarthritis}+ \beta_{osteoarthritis | diabetes}}$ becomes greater than one or becomes negative.

For example, let

$\beta_{diabetes, osteoarthritis}=500$

$$\beta_{diabetes | osteoarthritis}=300$$

$$\beta_{osteoarthritis | diabetes}=-100$$

Then,

$$\beta_{diabetes | combo}$$

$$= \beta_{diabetes, osteoarthritis} \times\frac{\beta_{diabetes | osteoarthritis}}{\beta_{diabetes | osteoarthritis}+ \beta_{osteoarthritis | diabetes}}=500*1.5$$

$$\beta_{osteoarthritis | combo}$$

$$= \beta_{diabetes, osteoarthritis} \times\frac{\beta_{osteoarthritis | diabetes}}{\beta_{diabetes | osteoarthritis}+ \beta_{osteoarthritis | diabetes}}=500*(-0.5)$$

The coefficient of the combination (500) would be split into +750 and – 250.

Consider another scenario in which the difference between the two coefficients are much smaller:

$\beta_{diabetes, osteoarthritis}=500$

$\beta_{diabetes | osteoarthritis}=100$00

$$\beta_{osteoarthritis | diabetes}=-9999$$

Then,

$$\beta_{diabetes | combo}$$

$$= \beta_{diabetes, osteoarthritis} \times\frac{\beta_{diabetes | osteoarthritis}}{\beta_{diabetes | osteoarthritis}+ \beta_{osteoarthritis | diabetes}}=500*10000$$

$$\beta_{osteoarthritis | combo}=$$

$$= \beta_{diabetes, osteoarthritis} \times\frac{\beta_{osteoarthritis | diabetes}}{\beta_{diabetes | osteoarthritis}+ \beta_{osteoarthritis | diabetes}}=500*(-9999)$$

In this example, the coefficient of the combination (500) would be split into +5,000,000 and -4,999,500. While the first example seemed reasonable, the second scenario is not. To avoid these unreasonable scenarios, we placed the following restriction on the range in which the proportion distributed to individual conditions should fall in:

$\beta_{diabetes, osteoarthritis}- 2SD< \beta_{diabetes | combo}< \beta_{diabetes, osteoarthritis}+ 2SD$

The proportion distributed each condition (in these examples, the proportion of the combination distributed to diabetes or osteoarthritis) cannot be greater than the estimated spending associated with the combination plus two standard deviation of the combination estimate, and cannot be less than the spending associated with the combination minus two of its standard deviation. This restriction was applied among 2.9% of all dyads and 28.1% of all triads.

1. **Stochastic gradient descent model parameter selection and summary statistics of the model runs**
2. **Model description and parameter selection**

Gradient descent is a common approach for optimizing a function to find a local minimum. The *stochastic* in stochastic gradient descent (SGD) is due to the shuffled nature of the data: the model randomly shuffles the dataset and runs it iteratively, compared to running the entire dataset as a single source (as in standard gradient descent).^[[1]](#footnote-1)^ In this paper, we implemented SGD for linear regression since it involved a large dataset and a large number of covariates (mainly composed of the two- and three-way combinations of chronic conditions) and cannot be run using a standard gradient descent. The model is fitted on the input data and the algorithm stops when a given level of pre-defined convergence is reached.

To first select a good set of model parameters, we first ran a multiple linear regression without the disease interaction terms (the dyads and triads) with the full dataset to gauge the approximate range of the estimates, R^2^, and mean squared error (MSE). Then, using the estimates from the multiple linear regression as a guide, we manually adjusted each of the SGD parameters listed below to get SGD estimates that are at the similar levels. The parameters needed for the SGD model include:

- Stopping criterion (tolerance): We set the stopping criterion as the difference between the R-squared of two consecutive model runs to be equal or less than 0.005. In other words, the criterion is evaluated after every run, and the algorithm stops when the difference between the R-squared of the previous run and the current run is less than our set threshold. We did not set the number of iterations required and relied on the tolerance level instead
- Learning rate: learning rate controls the step-size in the parameter space, i.e., the size of steps the model takes in any direction to optimize the cost function. We chose an adaptively decreasing learning rate with a starting learning rate of 0.00005
- Penalty: also called the regularization term. We chose L1 regularization (Lasso) to shrink the parameters to zero
- Size of the iteration files: 512 random rows for each iteration, shuffled
- Alpha, the constant that multiplies the regularization term: 0.01

**B. Summary statistics of the model runs**

Given the large size of the dataset (6.7+ billion claims), over 40,000 covariates representing all possible dyads and triads of chronic conditions, and the need for strong computational power, we applied the stochastic gradient descent (SGD) approach, a commonly used method in solving large machine learning problems. To ensure stability of the model results, we conducted 50 SGD model runs and bootstrapped the results across runs for 10,000 times to get the estimates for all coefficients. Table A3 summarizes the statistics across the SGD model runs.

Table A3. Summary statistics of 50 SGD model runs

|  | Mean | Median | Range | SD |
| --- | --- | --- | --- | --- |
| R2 | 0.320 | 0.324 | 0.165 – 0.417 | 0.058 |
| MSE | 983699001 | 796118493 | 434285235 -  5118039237 | 712178273 |
| Intercept | 574.3 | 546.4 | 226.6-965.1 | 183.0 |
| Female | -265.0 | -241.8 | -710.2 – 347.1 | 219.5 |
| Age group 2 | 1024.8 | 976.6 | 219.7-1426.7 | 286.9 |
| Age group 3 | 145.2 | 147.8 | -45.2 – 326.2 | 104.5 |
| Age group 4 | -519.3 | -488.6 | -952.7 – 77.3 | 238.3 |
| Age group 5 | -648.5 | -623.8 | -1421.3 – 549.0 | 492.8 |
| Region dummy 2 | -230.2 | -210.0 | -562.4 – 235.9 | 183.6 |
| Region dummy 3 | -623.9 | -632.5 | -951.1 - -109.5 | 167.1 |
| Region dummy 4 | 783.8 | 789.7 | 244.1-1184.8 | 196.7 |
| Region dummy 5 | -9.6 | -22.4 | -162.9 – 98.9 | 51.9 |

**4. Full results**

This section includes more detailed results from our analysis. Table A4 summarizes the regression coefficients from Equation (1) in the manuscript. Table A5 presents the results for the "other” conditions, and Table A6 lists estimated spending of disease combinations with prevalence rate of greater than 100 per 100,000.

Table A4. Covariates and their regression coefficients

| Variable | Regression coefficient (95% UI) |
| --- | --- |
| Intercept | 574.3 [524.5,624.5] |
| Female | -265.0 [-327.8, -205.8] |
| Age group 25-34 | 976.6 [899.9-1058.0] |
| Age group 35-44 | 142.8 [114.6-171.5] |
| Age group 45-54 | -488.6 [-557.2, -425.0] |
| Age group 55-64 | -623.8 [-760.4, -488.6] |
| Region 2 (North Central) | -209.7 [-261.6, -159.8] |
| Region 3 (South) | -632.5 [-678.8, -587.8] |
| Region 4 (West) | 789.7 [736.5, 844.5] |
| Region 5 (Unknown) | -22.4 [-36.3, -8.1] |

* Reference groups: age group 1 (ages 18-24) and region 1 (Northeast)

Table A5. Observed prevalence and estimated multimorbidity-adjusted annual spending per treated case for the “other” conditions

| Chronic condition | Observed prevalence rate (per 100,000) | Multimorbidity-adjusted annual spending per treated case [95% UI] |
| --- | --- | --- |
| Other neoplasms | 13,573.1 | 2446.0 [2370-2514] |
| Other cardiovascular diseases | 3,301.9 | 10784.8 [10235-11263] |
| Other digestive diseases | 25.4 | 2909.0 [2650-3196] |
| Other neurological disorders | 8,998.7 | 4131.3 [4028-4253] |
| Other mental disorders | 4,507.8 | 1119.5 [1072-1169] |
| Other chronic respiratory diseases | 10,922.0 | 1250.3 [1084-1392] |
| Other musculoskeletal disorders | 24,869.3 | 1582.3 [1397-1749] |

* estimated spending per treated case of 35-44 year old females from the South region

Table A6. Regression coefficients for disease combinations with prevalence rate of greater than 100 per 100,000 (ordered by spending)

| Disease combinations | Observed prevalence rate (per 100,000) | Estimated spending per treated case [95% UI] |
| --- | --- | --- |
| chronic kidney disease + EMBI-disorders + hemoglobinopathies and hemolytic anemias | 192 | 3111.1 [2500, 3798.2] |
| chronic kidney disease + hemoglobinopathies and hemolytic anemias | 323.4 | 3073.6 [2553.4, 3721.8] |
| chronic kidney disease + EMBI-disorders | 556 | 2887.3 [2331, 2993.8] |
| EMBI-disorders + hemoglobinopathies and hemolytic anemias | 1444.9 | 2478 [2016.4, 2931.4] |
| cirrhosis + EMBI-disorders + hemoglobinopathies and hemolytic anemias | 168 | 2336.5 [2144, 2891] |
| cirrhosis + hemoglobinopathies and hemolytic anemias | 290.9 | 2235.8 [2104.5, 2550.5] |
| chronic kidney disease + cirrhosis | 142.3 | 2101.8 [1789.6, 2340] |
| chronic kidney disease + hemoglobinopathies and hemolytic anemias + skin and subcutaneous diseases | 137.3 | 2083.4 [1813.4, 2468.7] |
| cirrhosis + EMBI-disorders | 551 | 2049.2 [1918.2, 2391.4] |
| chronic kidney disease + IHD | 277.6 | 1920.1 [1829.1, 2273.6] |
| gastritis and biliary diseases + EMBI-disorders | 241.6 | 1859.3 [1682.3, 2031.6] |
| chronic kidney disease + EMBI-disorders + skin and subcutaneous diseases | 216.9 | 1698.1 [1632.3, 1912.1] |
| gastritis and biliary diseases + hemoglobinopathies and hemolytic anemias | 130.8 | 1624.9 [1305.3, 1805.4] |
| EMBI-disorders + hemoglobinopathies and hemolytic anemias + skin and subcutaneous diseases | 584.5 | 1548.9 [1218.9, 1781.2] |
| chronic kidney disease + skin and subcutaneous diseases | 462.2 | 1523.3 [1316.5, 1698.9] |
| stroke + EMBI-disorders | 237.7 | 1499.9 [1149.1, 1711.9] |
| chronic kidney disease + IHD + EMBI-disorders | 142.6 | 1489.3 [1310, 1817.2] |
| cirrhosis + hemoglobinopathies and hemolytic anemias + skin and subcutaneous diseases | 125.2 | 1478.8 [1142.8, 1632] |
| gastritis and biliary diseases + cirrhosis | 194.6 | 1475 [1380.8, 1598.3] |
| chronic kidney disease + IHD + hemoglobinopathies and hemolytic anemias | 107.3 | 1459.6 [1154.7, 1582.1] |
| chronic kidney disease + hemoglobinopathies and hemolytic anemias + hypertension | 227.3 | 1443.7 [1308.3, 1629.5] |
| IHD + hemoglobinopathies and hemolytic anemias | 371.5 | 1413.8 [1321.6, 1482.3] |
| IHD + hypertension | 1441.4 | 1409.7 [1316.4, 1480.5] |
| IHD + EMBI-disorders + hemoglobinopathies and hemolytic anemias | 183.1 | 1386 [1110.2, 1623.7] |
| inflammatory bowel disease + EMBI-disorders | 244.2 | 1372.3 [1306.2, 1697] |
| IHD + EMBI-disorders | 636.9 | 1359.8 [1046, 1455] |
| atrial fibrillation and flutter + IHD | 211.7 | 1333.8 [1184.7, 1600.7] |
| stroke + hemoglobinopathies and hemolytic anemias | 133.3 | 1299.4 [1150.6, 1420.2] |
| inflammatory bowel disease + hemoglobinopathies and hemolytic anemias | 148 | 1248.8 [946.1, 1363.4] |
| cirrhosis + EMBI-disorders + skin and subcutaneous diseases | 221.1 | 1224.2 [1045.5, 1312.6] |
| EMBI-disorders + oral disorders | 131.2 | 1220.3 [1077.9, 1491] |
| EMBI-disorders + hemoglobinopathies and hemolytic anemias + anxiety disorders | 307.8 | 1194.3 [902.2, 1480.3] |
| chronic kidney disease + EMBI-disorders + hypertension | 372.3 | 1178.6 [1051.2, 1322.7] |
| stroke + hypertension | 401.9 | 1162.8 [964.4, 1262.4] |
| anxiety disorders + substance use disorders | 680.8 | 1092.4 [862.9, 1313.2] |
| gastritis and biliary diseases + skin and subcutaneous diseases | 235.9 | 1065.9 [981.1, 1106.7] |
| atrial fibrillation and flutter + EMBI-disorders | 193.3 | 1058.4 [869.2, 1279] |
| atrial fibrillation and flutter + hemoglobinopathies and hemolytic anemias | 106.1 | 1045 [926.2, 1192.3] |
| congenital birth defects + EMBI-disorders | 198.2 | 1035.4 [937, 1237.2] |
| chronic kidney disease + EMBI-disorders + gynecological diseases | 113.6 | 1030.7 [988.9, 1236.8] |
| substance use disorders + tobacco use | 357.7 | 1030 [850.3, 1245.2] |
| chronic kidney disease + diabetes + hemoglobinopathies and hemolytic anemias | 151.5 | 1019 [841.7, 1254.4] |
| gastritis and duodenitis, peptic ulcer disease + obesity | 1330 | 1007.5 [818.5, 1117] |
| cirrhosis + skin and subcutaneous diseases | 516.9 | 1001.6 [963.6, 1079.3] |
| chronic kidney disease + gynecological diseases | 233.3 | 974.4 [743.6, 1051.2] |
| chronic kidney disease + hypertension | 901 | 963.7 [739.8, 1026.7] |
| chronic kidney disease + IHD + skin and subcutaneous diseases | 109.9 | 953.6 [726.7, 991.6] |
| osteoarthritis + obesity | 927.5 | 948.2 [875.7, 1156.6] |
| chronic kidney disease + anxiety disorders | 190.8 | 931.1 [785.6, 961] |
| inflammatory bowel disease + skin and subcutaneous diseases | 332.1 | 929.8 [725.7, 1094.3] |
| chronic kidney disease + IHD + hypertension | 218.6 | 929.8 [804.3, 1015.4] |
| IHD + stroke | 161.6 | 918 [790.6, 1086.5] |
| IHD + hyperlipidemia | 1285.5 | 917.9 [742.9, 1140.7] |
| gastritis and biliary diseases + hypertension | 268.2 | 912.6 [876.5, 975.4] |
| EMBI-disorders + hemoglobinopathies and hemolytic anemias + hypertension | 633.7 | 903.7 [695.2, 1113.7] |
| cirrhosis + EMBI-disorders + anxiety disorders | 120 | 898.2 [733, 1095.4] |
| EMBI-disorders + epilepsy | 100.5 | 897.5 [832.9, 1017.4] |
| cirrhosis + anxiety disorders | 269.3 | 887 [857.4, 931.1] |
| inguinal, femoral, and abdominal hernia + obesity | 251.8 | 885.5 [776.9, 935.6] |
| substance use disorders + depressive disorders | 522.5 | 879.6 [682.4, 997.1] |
| chronic kidney disease + gastritis and duodenitis, peptic ulcer disease + EMBI-disorders | 114.8 | 872 [694.6, 992.9] |
| IHD + tobacco use | 233.6 | 850.4 [812.2, 885.1] |
| stroke + EMBI-disorders + hypertension | 151.5 | 843.6 [733.2, 956.3] |
| IHD + hemoglobinopathies and hemolytic anemias + skin and subcutaneous diseases | 153.7 | 838 [666.4, 1001.7] |
| IHD + cirrhosis | 137.4 | 804.8 [690.8, 878.1] |
| substance use disorders + skin and subcutaneous diseases | 464.9 | 795.2 [745.9, 974.2] |
| chronic kidney disease + gastritis and duodenitis, peptic ulcer disease | 221.5 | 785.8 [649.6, 852] |
| gastritis and biliary diseases + EMBI-disorders + hypertension | 110.5 | 770.9 [622.8, 806] |
| congenital birth defects + hypertension | 218.9 | 770.2 [648.1, 871] |
| atrial fibrillation and flutter + IHD + hypertension | 153.1 | 769.8 [705.2, 841] |
| chronic kidney disease + diabetes + EMBI-disorders | 262.2 | 766.4 [661.1, 957.7] |
| EMBI-disorders + tobacco use | 494.2 | 759.4 [721.2, 850.7] |
| IHD + diabetes | 751.5 | 752.6 [713.8, 860.1] |
| inflammatory bowel disease + EMBI-disorders + skin and subcutaneous diseases | 102 | 747.8 [640.9, 838.8] |
| IHD + hyperlipidemia + hypertension | 958.7 | 746.2 [681.7, 911.9] |
| oral disorders + skin and subcutaneous diseases | 261.4 | 745.2 [630.1, 809.5] |
| stroke + EMBI-disorders + skin and subcutaneous diseases | 114 | 744.3 [658.2, 848.8] |
| anxiety disorders + substance use disorders + depressive disorders | 353.6 | 741.7 [616.1, 904.3] |
| osteoarthritis + hypertension | 1512.2 | 733 [699.1, 878.2] |
| cirrhosis + gynecological diseases | 338.2 | 730.9 [581.1, 815.6] |
| EMBI-disorders + substance use disorders | 386.3 | 729.7 [575.1, 856.7] |
| atrial fibrillation and flutter + hypertension | 376.8 | 721.8 [594.1, 772.3] |
| epilepsy + skin and subcutaneous diseases | 121.1 | 692.3 [661.9, 857.8] |
| cirrhosis + EMBI-disorders + gynecological diseases | 142.5 | 688.1 [544.3, 790.2] |
| diabetes + gastritis and biliary diseases | 139.5 | 684.9 [580.5, 788.4] |
| IHD + anxiety disorders | 302 | 675.8 [552.6, 812.6] |
| stroke + skin and subcutaneous diseases | 291.5 | 667.9 [571.1, 708.9] |
| cirrhosis + hemoglobinopathies and hemolytic anemias + hypertension | 149.7 | 654 [561.1, 795.1] |
| inguinal, femoral, and abdominal hernia + hypertension | 351 | 646.6 [487.4, 737.3] |
| gastritis and biliary diseases + gynecological diseases | 198.3 | 646 [510, 783.2] |
| osteoarthritis + hyperlipidemia | 1126.4 | 643.7 [550.9, 799.9] |
| gastritis and biliary diseases + anxiety disorders | 142.3 | 638.7 [551.9, 764.1] |
| diabetes + EMBI-disorders + hemoglobinopathies and hemolytic anemias | 392 | 632.6 [585.3, 674.4] |
| hemoglobinopathies and hemolytic anemias + anxiety disorders | 884.4 | 631.2 [494, 667] |
| cirrhosis + colon and rectum cancer | 118.1 | 630 [535, 673.2] |
| epilepsy + hypertension | 110.3 | 624.5 [588.4, 765.3] |
| chronic kidney disease + hypertension + skin and subcutaneous diseases | 306 | 622.8 [493.7, 671.7] |
| inguinal, femoral, and abdominal hernia + EMBI-disorders | 229.8 | 622.6 [591.4, 746.4] |
| chronic kidney disease + diabetes | 641.1 | 622.3 [554, 657.7] |
| hypertension + tobacco use | 957.2 | 622.1 [497.1, 703.4] |
| IHD + hemoglobinopathies and hemolytic anemias + hypertension | 270 | 619.4 [540.9, 693] |
| stroke + anxiety disorders | 135.9 | 608.9 [582, 639.6] |
| EMBI-disorders + COPD | 218 | 599.7 [493.4, 648.8] |
| gastritis and duodenitis, peptic ulcer disease + osteoarthritis | 578.1 | 594.3 [483.6, 685.5] |
| peripheral vascular disease+ hypertension | 230.1 | 588.4 [462.6, 692.9] |
| gastritis and duodenitis, peptic ulcer disease + EMBI-disorders + hemoglobinopathies and hemolytic anemias | 340.7 | 585.1 [565.5, 665.4] |
| cirrhosis + anxiety disorders + skin and subcutaneous diseases | 110.8 | 584.8 [558, 628.4] |
| chronic kidney disease + IHD + diabetes | 157.6 | 582.8 [448.6, 629] |
| chronic kidney disease + diabetes + skin and subcutaneous diseases | 226.2 | 580.8 [524.1, 665.6] |
| IHD + EMBI-disorders + skin and subcutaneous diseases | 250.1 | 580.4 [546.2, 683.3] |
| IHD + stroke + hypertension | 126.3 | 572.4 [472.5, 688.8] |
| hemoglobinopathies and hemolytic anemias + substance use disorders | 211.9 | 570 [456.2, 672.2] |
| IHD + skin and subcutaneous diseases | 687.6 | 568.3 [539.7, 629.4] |
| gastritis and biliary diseases + obesity | 214 | 558.6 [484.1, 648.2] |
| atrial fibrillation and flutter + EMBI-disorders + hypertension | 129.7 | 548.2 [484.7, 650.2] |
| IHD + hyperlipidemia + tobacco use | 145.4 | 537.1 [427.7, 644.6] |
| stroke + sense organ diseases | 177 | 533.6 [410.5, 641.9] |
| gastritis and biliary diseases + gastritis and duodenitis, peptic ulcer disease | 231.1 | 531.5 [418.4, 612.5] |
| chronic kidney disease + gynecological diseases + hypertension | 142.9 | 530.2 [425.8, 610.3] |
| congenital birth defects + gynecological diseases | 202.6 | 522.9 [499.2, 571.2] |
| hemoglobinopathies and hemolytic anemias + anxiety disorders + skin and subcutaneous diseases | 358.7 | 510.6 [399.8, 567.3] |
| IHD + EMBI-disorders + anxiety disorders | 118.7 | 503.7 [464.4, 604.2] |
| EMBI-disorders + gynecological diseases + hemoglobinopathies and hemolytic anemias | 444.4 | 503.4 [409.7, 563.2] |
| anxiety disorders + substance use disorders + tobacco use | 163.9 | 498.8 [437.9, 528.4] |
| stroke + diabetes | 198.6 | 498.5 [374.6, 594.2] |
| gastritis and duodenitis, peptic ulcer disease + depressive disorders | 694.1 | 497.7 [471.3, 553.8] |
| atrial fibrillation and flutter + skin and subcutaneous diseases | 212.4 | 490.4 [404.7, 514] |
| inguinal, femoral, and abdominal hernia + skin and subcutaneous diseases | 312.2 | 489.6 [409.3, 530.2] |
| osteoarthritis + hypertension + obesity | 554.1 | 488.1 [393.2, 508.8] |
| atrial fibrillation and flutter + IHD + hyperlipidemia | 116.3 | 487.6 [404.2, 606.8] |
| IHD + diabetes + hypertension | 587.8 | 487.3 [424.4, 606.1] |
| atrial fibrillation and flutter + diabetes | 169 | 486.6 [432.4, 606.5] |
| IHD + hypertension + tobacco use | 171.7 | 486.2 [419.4, 519.3] |
| gynecological diseases + hemoglobinopathies and hemolytic anemias | 1372.1 | 485.3 [382.2, 535.1] |
| gynecological diseases + obesity | 2151.9 | 481.1 [449.8, 517.8] |
| tobacco use + skin and subcutaneous diseases | 697.9 | 480.1 [392.4, 592.9] |
| gastritis and duodenitis, peptic ulcer disease + EMBI-disorders | 1430.3 | 474.9 [432.7, 491.8] |
| bipolar disorder + substance use disorders | 136.3 | 473.2 [419.2, 557.2] |
| gastritis and duodenitis, peptic ulcer disease + substance use disorders | 242.2 | 472 [355.8, 557.3] |
| IHD + peripheral vascular disease | 104.8 | 472 [440.2, 541.6] |
| IHD + EMBI-disorders + hypertension | 447.3 | 470 [396.6, 487.8] |
| peripheral vascular disease+ diabetes | 138.1 | 468 [416.5, 540.8] |
| congenital birth defects + anxiety disorders | 140.8 | 465.3 [369.3, 527.5] |
| cirrhosis + gastritis and duodenitis, peptic ulcer disease + hemoglobinopathies and hemolytic anemias | 105.6 | 465 [449.3, 491.1] |
| asthma + obesity | 649.4 | 459.8 [359.2, 519.8] |
| inflammatory bowel disease + gynecological diseases | 216.2 | 457.2 [407.6, 496.9] |
| inflammatory bowel disease + gastritis and duodenitis, peptic ulcer disease | 237 | 454.6 [381.7, 565] |
| gynecological diseases + substance use disorders | 273.7 | 447.4 [369.7, 517.3] |
| hemoglobinopathies and hemolytic anemias + skin and subcutaneous diseases | 1695.9 | 439.5 [373.4, 469.4] |
| substance use disorders + hypertension | 565.6 | 436.8 [380.1, 542.6] |
| inflammatory bowel disease + anxiety disorders | 190.1 | 431.6 [327.8, 500] |
| peripheral vascular disease+ skin and subcutaneous diseases | 216.9 | 427.9 [395.2, 451.8] |
| EMBI-disorders + hemoglobinopathies and hemolytic anemias + depressive disorders | 203 | 426.2 [378.8, 447.8] |
| cirrhosis + substance use disorders | 120.2 | 422.8 [329.9, 436.3] |
| stroke + gynecological diseases | 129.5 | 422.4 [373.2, 485.3] |
| bipolar disorder + depressive disorders | 241.2 | 420.3 [370.9, 478.2] |
| inguinal, femoral, and abdominal hernia + hypertension + obesity | 134.3 | 416.6 [314.6, 478.9] |
| substance use disorders + depressive disorders + tobacco use | 137.1 | 415.9 [390.5, 451.8] |
| oral disorders + hypertension | 146.1 | 415.7 [331.1, 430.9] |
| diabetes + hemoglobinopathies and hemolytic anemias + skin and subcutaneous diseases | 351 | 413.7 [332.4, 442.6] |
| anxiety disorders + oral disorders | 113.8 | 412.8 [384.1, 508.5] |
| peripheral vascular disease+ diabetes + hypertension | 104.3 | 406.1 [390.1, 505.7] |
| inguinal, femoral, and abdominal hernia + gastritis and duodenitis, peptic ulcer disease + obesity | 143.3 | 406 [383.7, 465.5] |
| congenital birth defects + skin and subcutaneous diseases | 304.6 | 404.1 [332.8, 497.4] |
| low back and neck pain + tobacco use | 618.6 | 402.9 [367.6, 486.6] |
| IHD + diabetes + hemoglobinopathies and hemolytic anemias | 158.8 | 402.4 [363.2, 440.1] |
| gynecological diseases + breast cancer | 1692.9 | 401.6 [365.4, 433.1] |
| anxiety disorders + tobacco use | 605.6 | 401.1 [347.7, 468.2] |
| congenital birth defects + gastritis and duodenitis, peptic ulcer disease | 105.6 | 399.4 [374.3, 453.1] |
| osteoarthritis + low back and neck pain | 1242.5 | 394.4 [375.9, 476.7] |
| peripheral vascular disease+ hypertension + skin and subcutaneous diseases | 113.7 | 390.8 [331.9, 460] |
| cirrhosis + gastritis and duodenitis, peptic ulcer disease + EMBI-disorders | 167.9 | 389.6 [374.6, 402.5] |
| hyperlipidemia + tobacco use | 669.3 | 387.6 [367.8, 474.8] |
| inguinal, femoral, and abdominal hernia + hemoglobinopathies and hemolytic anemias | 145.9 | 381.5 [347.2, 394.3] |
| osteoarthritis + hyperlipidemia + hypertension | 711.7 | 380.1 [302.4, 462.2] |
| osteoarthritis + hyperlipidemia + obesity | 378.4 | 375.2 [281.6, 428.6] |
| gastritis and duodenitis, peptic ulcer disease + hypertension + obesity | 699.5 | 374.5 [362, 400.2] |
| anxiety disorders + depressive disorders | 3031.1 | 374.1 [323.3, 431.3] |
| anxiety disorders + substance use disorders + skin and subcutaneous diseases | 192.7 | 372.9 [314.2, 398.7] |
| EMBI-disorders + substance use disorders + skin and subcutaneous diseases | 139.3 | 371.6 [312.7, 408.9] |
| EMBI-disorders + anxiety disorders | 1845.6 | 371.1 [357.7, 389.6] |
| depressive disorders + tobacco use | 411.6 | 370.1 [278.4, 454.1] |
| cirrhosis + EMBI-disorders + hypertension | 274.2 | 368 [279.4, 391.1] |
| atrial fibrillation and flutter + hyperlipidemia | 271.4 | 363.7 [309.1, 430.2] |
| peripheral vascular disease+ hyperlipidemia | 194.1 | 363.5 [302.9, 450.6] |
| hyperlipidemia + hypertension + tobacco use | 419.9 | 362.3 [343.7, 447.5] |
| cirrhosis + depressive disorders | 175.7 | 360.2 [310.4, 446.1] |
| peripheral vascular disease+ hyperlipidemia + hypertension | 138.2 | 359.6 [333.7, 399] |
| congenital birth defects + obesity | 129 | 357.9 [325.3, 378.8] |
| gynecological diseases + asthma | 697.5 | 353.5 [271.3, 428.7] |
| inflammatory bowel disease + hypertension | 256.4 | 351.3 [311.7, 368.7] |
| hemoglobinopathies and hemolytic anemias + hypertension | 1724.4 | 349.1 [335.3, 367.3] |
| gastritis and duodenitis, peptic ulcer disease + osteoarthritis + obesity | 210.5 | 349 [285.5, 395.7] |
| inguinal, femoral, and abdominal hernia + gynecological diseases | 194.1 | 348.8 [303.1, 410.7] |
| IHD + obesity | 593.2 | 348.1 [310.1, 383.2] |
| chronic kidney disease + IHD + hyperlipidemia | 169.3 | 347.6 [301.6, 412] |
| anxiety disorders + bipolar disorder + depressive disorders | 174.6 | 347.1 [277, 365.3] |
| chronic kidney disease + hemoglobinopathies and hemolytic anemias + hyperlipidemia | 154.7 | 344.4 [314.8, 401] |
| hemoglobinopathies and hemolytic anemias + anxiety disorders + hypertension | 318.1 | 340.4 [317.1, 385.4] |
| stroke + diabetes + hypertension | 156.3 | 336.1 [310.9, 356.1] |
| COPD + hypertension | 363.8 | 334 [273.6, 391.2] |
| anxiety disorders + bipolar disorder | 399.8 | 334 [273, 386.5] |
| gastritis and duodenitis, peptic ulcer disease + tobacco use | 355.1 | 331.6 [302.5, 358.3] |
| COPD + skin and subcutaneous diseases | 227.1 | 330.4 [267.7, 349.2] |
| diabetes + hemoglobinopathies and hemolytic anemias + anxiety disorders | 152.6 | 329.8 [300.6, 342.5] |
| IHD + COPD | 148.7 | 328 [258.6, 351.1] |
| gastritis and duodenitis, peptic ulcer disease + anxiety disorders + depressive disorders | 397.3 | 327.1 [282.1, 399.4] |
| osteoarthritis + asthma | 237.5 | 326.5 [248.1, 367.8] |
| IHD + gastritis and duodenitis, peptic ulcer disease | 361.8 | 324.2 [284.6, 356.2] |
| chronic kidney disease + hemoglobinopathies and hemolytic anemias + obesity | 107.3 | 323.6 [249.4, 387.2] |
| low back and neck pain + asthma | 698.3 | 322.8 [278.8, 372.6] |
| atrial fibrillation and flutter + diabetes + hypertension | 134 | 320.3 [245.2, 353.8] |
| congenital birth defects + low back and neck pain | 204 | 319.9 [255.3, 378.3] |
| stroke + hypertension + skin and subcutaneous diseases | 172 | 319.6 [283.2, 365] |
| diabetes + hemoglobinopathies and hemolytic anemias | 924.9 | 315.9 [255, 334.8] |
| diabetes + inguinal, femoral, and abdominal hernia | 154.5 | 315.3 [247.4, 339.9] |
| hemoglobinopathies and hemolytic anemias + COPD | 197.3 | 310.4 [288.5, 321.5] |
| EMBI-disorders + anxiety disorders + substance use disorders | 159.4 | 309.6 [241.3, 371.2] |
| IHD + anxiety disorders + skin and subcutaneous diseases | 120 | 309 [253.9, 386] |
| gastritis and duodenitis, peptic ulcer disease + hypertension | 2170.9 | 306 [296.4, 364] |
| IHD + diabetes + EMBI-disorders | 271.1 | 302.9 [280.1, 371.8] |
| cirrhosis + gastritis and duodenitis, peptic ulcer disease | 398.6 | 300.9 [240.5, 324.5] |
| cirrhosis + gynecological diseases + skin and subcutaneous diseases | 146.8 | 299 [240.7, 369.2] |
| IHD + EMBI-disorders + gynecological diseases | 119.2 | 298.2 [285.2, 336.2] |
| substance use disorders + asthma | 103 | 297.2 [285.5, 320.8] |
| cirrhosis + gynecological diseases + hypertension | 134 | 294.4 [259.2, 326.4] |
| peripheral vascular disease+ EMBI-disorders | 145.8 | 291.9 [235.5, 364.2] |
| IHD + diabetes + hyperlipidemia | 541.2 | 290.8 [263.6, 343.1] |
| hemoglobinopathies and hemolytic anemias + hypertension + skin and subcutaneous diseases | 622.2 | 289 [237.4, 317.3] |
| IHD + anxiety disorders + hypertension | 207.2 | 288.9 [244.6, 339.5] |
| cirrhosis + hypertension | 649.6 | 288 [274.2, 327.4] |
| inguinal, femoral, and abdominal hernia + gastritis and duodenitis, peptic ulcer disease | 445.3 | 286 [260.4, 350.6] |
| gastritis and duodenitis, peptic ulcer disease + osteoarthritis + hypertension | 310.2 | 285.8 [271.2, 346.3] |
| chronic kidney disease + anxiety disorders + hypertension | 123.5 | 285.8 [224.5, 318.2] |
| diabetes + cirrhosis + EMBI-disorders | 185.3 | 285.5 [254.7, 332.8] |
| inguinal, femoral, and abdominal hernia + hyperlipidemia | 267.1 | 283 [255.8, 305.6] |
| low back and neck pain + obesity | 2147.4 | 282.5 [218, 316.2] |
| osteoarthritis + tobacco use | 187.1 | 282.1 [230.1, 330.2] |
| stroke + hypertension + sense organ diseases | 107 | 281.2 [215.6, 323.2] |
| anxiety disorders + depressive disorders + tobacco use | 255 | 279 [260.9, 309] |
| low back and neck pain + hyperlipidemia | 2757.1 | 278.5 [264.9, 287.8] |
| inguinal, femoral, and abdominal hernia + anxiety disorders | 160.6 | 278.1 [220, 346.9] |
| EMBI-disorders + tobacco use + skin and subcutaneous diseases | 173.7 | 278 [234.4, 323.8] |
| chronic kidney disease + depressive disorders | 150.9 | 274.3 [239.5, 327.2] |
| congenital birth defects + hyperlipidemia | 159.6 | 272 [217.9, 301.9] |
| gynecological diseases + hemoglobinopathies and hemolytic anemias + hypertension | 411.1 | 267.1 [238.8, 299.9] |
| depressive disorders + osteoarthritis | 390.5 | 267.1 [210.9, 281] |
| low back and neck pain + skin and subcutaneous diseases | 4554.5 | 266.6 [229.1, 299.9] |
| gastritis and duodenitis, peptic ulcer disease + osteoarthritis + hyperlipidemia | 241.7 | 266.1 [200.5, 317.3] |
| diabetes + skin and subcutaneous diseases | 2454.9 | 265.2 [218.9, 294.3] |
| gynecological diseases + reproductive organ cancers | 1081.7 | 263.4 [240.6, 314.3] |
| stroke + low back and neck pain | 204.8 | 260.1 [227.7, 268.9] |
| atrial fibrillation and flutter + obesity | 180 | 259.4 [223, 311.9] |
| IHD + gynecological diseases | 291.5 | 257.9 [217, 274.6] |
| IHD + stroke + hyperlipidemia | 109.6 | 253 [238.9, 278.7] |
| diabetes + inflammatory bowel disease | 126 | 252.6 [227.8, 308.3] |
| obesity + tobacco use | 467.9 | 252.1 [208.3, 311.7] |
| diabetes + EMBI-disorders + anxiety disorders | 323.1 | 249.3 [191.4, 286.9] |
| anxiety disorders + COPD | 148.1 | 248.4 [225.9, 289.5] |
| diabetes + tobacco use | 396 | 247.4 [235.3, 265.8] |
| gastritis and duodenitis, peptic ulcer disease + low back and neck pain | 1571.6 | 242.6 [210.7, 284.5] |
| gynecological diseases + low back and neck pain | 3000.9 | 242.2 [199.3, 283.7] |
| low back and neck pain + sense organ diseases | 1444.5 | 241.5 [216.9, 257.8] |
| cirrhosis + anxiety disorders + depressive disorders | 100.4 | 237.8 [204.5, 283] |
| diabetes + COPD | 178.7 | 233.2 [204.6, 244.8] |
| depressive disorders + low back and neck pain | 1396.4 | 231.1 [178.8, 250.6] |
| EMBI-disorders + anxiety disorders + skin and subcutaneous diseases | 723.4 | 230.4 [201.6, 269.8] |
| gastritis and duodenitis, peptic ulcer disease + anxiety disorders | 1182.9 | 229.9 [214.4, 253.3] |
| congenital birth defects + sense organ diseases | 120.1 | 229.7 [221.6, 283.8] |
| chronic kidney disease + EMBI-disorders + low back and neck pain | 136.7 | 227.3 [186.5, 269.1] |
| low back and neck pain + hypertension | 3510.4 | 226 [207.8, 241.7] |
| gastritis and biliary diseases + low back and neck pain | 174.3 | 225.9 [190.2, 281.9] |
| diabetes + gastritis and duodenitis, peptic ulcer disease | 983.4 | 225.5 [201.9, 277.1] |
| atrial fibrillation and flutter + hypertension + skin and subcutaneous diseases | 131 | 222.8 [200.4, 231.3] |
| hemoglobinopathies and hemolytic anemias + depressive disorders | 542.4 | 221.6 [192, 258.8] |
| gynecological diseases + COPD | 107.2 | 221 [176.9, 231.3] |
| gastritis and duodenitis, peptic ulcer disease + EMBI-disorders + depressive disorders | 225.9 | 217.9 [176.9, 268.7] |
| substance use disorders + depressive disorders + skin and subcutaneous diseases | 147.4 | 216.4 [162.9, 264.9] |
| atrial fibrillation and flutter + hyperlipidemia + hypertension | 202.7 | 216.4 [208.4, 248.8] |
| hyperlipidemia + hypertension + obesity | 1914.3 | 213.3 [169.3, 262.1] |
| cirrhosis + tobacco use | 114.3 | 212.1 [163.1, 233.9] |
| gastritis and duodenitis, peptic ulcer disease + COPD | 151.6 | 210.9 [161.1, 230.3] |
| depressive disorders + asthma | 345.4 | 210.8 [195.7, 217.7] |
| chronic kidney disease + gastritis and duodenitis, peptic ulcer disease + hypertension | 156.4 | 210.6 [181.5, 255.2] |
| gynecological diseases + reproductive organ cancers + hypertension | 202.4 | 209.7 [191.5, 236.9] |
| gynecological diseases + anxiety disorders + substance use disorders | 129.1 | 208.4 [178.8, 243.9] |
| gynecological diseases + breast cancer + skin and subcutaneous diseases | 698.3 | 208.4 [169.5, 216] |
| substance use disorders + sense organ diseases | 133.3 | 208.1 [198.3, 254.8] |
| gastritis and duodenitis, peptic ulcer disease + asthma | 502.2 | 207.8 [185, 214.2] |
| atrial fibrillation and flutter + diabetes + hyperlipidemia | 108.1 | 206.8 [180.3, 250.1] |
| gastritis and duodenitis, peptic ulcer disease + depressive disorders + obesity | 225.2 | 206.6 [184.4, 247.6] |
| hemoglobinopathies and hemolytic anemias + depressive disorders + skin and subcutaneous diseases | 219.4 | 205.8 [164.9, 229.8] |
| inguinal, femoral, and abdominal hernia + EMBI-disorders + hypertension | 111.3 | 204.8 [184, 222.9] |
| IHD + gastritis and duodenitis, peptic ulcer disease + hemoglobinopathies and hemolytic anemias | 102.9 | 202.5 [161.3, 253] |
| gynecological diseases + substance use disorders + skin and subcutaneous diseases | 103.2 | 201.8 [167.6, 230.4] |
| substance use disorders + low back and neck pain + tobacco use | 118.5 | 200.9 [181.9, 233.1] |
| gynecological diseases + oral disorders | 130.3 | 199.6 [185.2, 244.3] |
| gastritis and duodenitis, peptic ulcer disease + depressive disorders + skin and subcutaneous diseases | 272.6 | 199.1 [187.7, 231.8] |
| EMBI-disorders + gynecological diseases + reproductive organ cancers | 202.7 | 196.8 [163.6, 240.7] |
| substance use disorders + low back and neck pain | 673.2 | 196 [162.4, 230.4] |
| gynecological diseases + anxiety disorders + breast cancer | 278.4 | 195.8 [184.1, 211.2] |
| gynecological diseases + reproductive organ cancers + obesity | 169.1 | 195.7 [168.7, 226] |
| headache disorders + sense organ diseases | 355.9 | 193.1 [183.2, 216.5] |
| substance use disorders + obesity | 278.5 | 192.8 [178.5, 235.9] |
| IHD + hypertension + skin and subcutaneous diseases | 464 | 191.7 [177.1, 218.4] |
| gynecological diseases + hypertension | 2593.4 | 191.1 [151.2, 208] |
| inflammatory bowel disease + depressive disorders | 114.5 | 189.7 [182.6, 201.1] |
| substance use disorders + depressive disorders + low back and neck pain | 177.9 | 187.6 [157.2, 195.7] |
| chronic kidney disease + low back and neck pain + skin and subcutaneous diseases | 127.3 | 187.5 [174.5, 201.5] |
| osteoarthritis + low back and neck pain + obesity | 345.7 | 187.1 [157.5, 200.5] |
| anxiety disorders + osteoarthritis | 548.6 | 186.4 [152.2, 213.7] |
| cirrhosis + anxiety disorders + hypertension | 126.3 | 183.4 [173.4, 200.8] |
| EMBI-disorders + gynecological diseases + hypertension | 769.5 | 183.1 [139.2, 195.4] |
| diabetes + EMBI-disorders + skin and subcutaneous diseases | 792.9 | 182.5 [154.5, 220.5] |
| depressive disorders + obesity | 1071.3 | 182.3 [144.1, 217] |
| asthma + hypertension | 850.3 | 179.9 [161.7, 210.7] |
| Attention-deficit / hyperactivity disorder + substance use disorders | 122.9 | 179.7 [174.1, 196.5] |
| stroke + gastritis and duodenitis, peptic ulcer disease | 117.1 | 179.3 [153.1, 196.5] |
| gastritis and duodenitis, peptic ulcer disease + depressive disorders + hypertension | 284 | 177.6 [148.4, 193.2] |
| gynecological diseases + hemoglobinopathies and hemolytic anemias + reproductive organ cancers | 102.6 | 177.4 [145.5, 205.5] |
| diabetes + anxiety disorders | 948.5 | 177.1 [143, 220] |
| EMBI-disorders + thyroid cancer | 115.3 | 177.1 [136.9, 203.9] |
| hemoglobinopathies and hemolytic anemias + reproductive organ cancers | 267.3 | 174.8 [152.8, 203.5] |
| anxiety disorders + substance use disorders + low back and neck pain | 242.6 | 172.7 [129.9, 182] |
| gastritis and duodenitis, peptic ulcer disease + hyperlipidemia + obesity | 503.2 | 171.3 [163.8, 210.7] |
| IHD + hyperlipidemia + obesity | 376.8 | 171.3 [132.1, 194.4] |
| EMBI-disorders + skin and subcutaneous diseases | 3772.7 | 169.7 [152, 195.8] |
| IHD + diabetes + skin and subcutaneous diseases | 258.9 | 169.3 [147.9, 193.4] |
| gastritis and duodenitis, peptic ulcer disease + hemoglobinopathies and hemolytic anemias + depressive disorders | 144.7 | 167.7 [132.1, 199.8] |
| osteoarthritis + skin and subcutaneous diseases | 1325.5 | 166.7 [129.2, 172.9] |
| IHD + depressive disorders | 199.1 | 166 [145.8, 197] |
| gastritis and duodenitis, peptic ulcer disease + osteoarthritis + low back and neck pain | 250.6 | 165.5 [148.8, 177.6] |
| IHD + gynecological diseases + skin and subcutaneous diseases | 128 | 164.1 [135.6, 198.4] |
| hemoglobinopathies and hemolytic anemias + anxiety disorders + depressive disorders | 310 | 163.4 [133.2, 171.7] |
| gastritis and duodenitis, peptic ulcer disease + gynecological diseases | 1355.9 | 162.2 [130, 194.8] |
| inflammatory bowel disease + obesity | 153.7 | 162.2 [131.9, 187] |
| inguinal, femoral, and abdominal hernia + hyperlipidemia + hypertension | 160.3 | 161.7 [129.1, 193.3] |
| diabetes + gastritis and duodenitis, peptic ulcer disease + EMBI-disorders | 347.5 | 160.9 [136.4, 182.9] |
| anxiety disorders + breast cancer | 675.1 | 160.7 [140.7, 179.8] |
| inguinal, femoral, and abdominal hernia + hypertension + skin and subcutaneous diseases | 124.1 | 160.2 [131.7, 179.1] |
| chronic kidney disease + EMBI-disorders + obesity | 174 | 159.7 [130.7, 169.2] |
| EMBI-disorders + depressive disorders | 1153 | 159.6 [138, 173.7] |
| gastritis and biliary diseases + hyperlipidemia | 181.4 | 159.3 [134.9, 187] |
| hyperlipidemia + sense organ diseases | 1836.6 | 159 [136.4, 175.3] |
| hemoglobinopathies and hemolytic anemias + colon and rectum cancer | 267.9 | 158.1 [120.4, 184.2] |
| stroke + hyperlipidemia + hypertension | 245.9 | 157.7 [147.3, 183.8] |
| IHD + substance use disorders | 113 | 156.4 [123, 176.2] |
| osteoarthritis + sense organ diseases | 550.2 | 155.6 [143.8, 177.3] |
| gastritis and biliary diseases + hypertension + obesity | 100.7 | 155.6 [126.3, 178.1] |
| depressive disorders + hypertension | 1392.8 | 154.9 [123.7, 168.3] |
| depressive disorders + osteoarthritis + obesity | 139.3 | 154.4 [135.9, 192.9] |
| osteoarthritis + low back and neck pain + hyperlipidemia | 413.3 | 152 [129.5, 184.6] |
| anxiety disorders + sense organ diseases | 849 | 151.3 [119.6, 184.3] |
| anxiety disorders + asthma | 563.3 | 150.8 [123.1, 164.5] |
| osteoarthritis + asthma + hypertension | 120.8 | 150.8 [143.5, 166.2] |
| EMBI-disorders + hypertension + tobacco use | 238.5 | 150.7 [122.7, 166.3] |
| IHD + hypertension + obesity | 457.3 | 148.4 [112.7, 167.3] |
| asthma + hypertension + obesity | 304.5 | 147.7 [131.1, 173.7] |
| diabetes + hypertension + tobacco use | 254.7 | 146.7 [138.9, 169.6] |
| EMBI-disorders + substance use disorders + depressive disorders | 125.8 | 142.8 [112.6, 174.5] |
| inguinal, femoral, and abdominal hernia + low back and neck pain | 233.2 | 142.4 [111.2, 177.4] |
| depressive disorders + skin and subcutaneous diseases | 1657.8 | 142.2 [117.2, 155.7] |
| diabetes + inguinal, femoral, and abdominal hernia + hypertension | 101.5 | 142.2 [108.8, 152.6] |
| cirrhosis + hypertension + skin and subcutaneous diseases | 236.2 | 142.1 [109.9, 161.5] |
| gastritis and duodenitis, peptic ulcer disease + hemoglobinopathies and hemolytic anemias | 898 | 139.1 [134.5, 170.4] |
| chronic kidney disease + EMBI-disorders + sense organ diseases | 108.2 | 138 [110.1, 168.4] |
| EMBI-disorders + anxiety disorders + hypertension | 625.4 | 136.4 [118, 170.3] |
| depressive disorders + breast cancer | 388.1 | 136 [125.1, 168.3] |
| diabetes + EMBI-disorders | 2342.6 | 135.8 [106.8, 152.2] |
| inflammatory bowel disease + low back and neck pain | 220.8 | 135.3 [104.9, 158.6] |
| gastritis and duodenitis, peptic ulcer disease + reproductive organ cancers | 311.1 | 135.1 [119.3, 149.4] |
| EMBI-disorders + skin cancers | 152.3 | 134.9 [124.9, 154.5] |
| anxiety disorders + depressive disorders + asthma | 199.7 | 134.6 [119.2, 144.4] |
| skin cancers + skin and subcutaneous diseases | 639.4 | 134.2 [111.3, 167.7] |
| obesity + skin and subcutaneous diseases | 2846 | 134 [106.4, 162.7] |
| gastritis and duodenitis, peptic ulcer disease + asthma + obesity | 162.9 | 132.4 [102.9, 164.6] |
| depressive disorders + sense organ diseases | 527.4 | 132.1 [107.4, 164] |
| Attention-deficit / hyperactivity disorder + bipolar disorder | 113.4 | 131.6 [110.9, 159] |
| asthma + tobacco use | 159.9 | 129.9 [123.3, 142.9] |
| gastritis and duodenitis, peptic ulcer disease + anxiety disorders + substance use disorders | 110.1 | 129.7 [98.6, 146.1] |
| gynecological diseases + tobacco use | 425.4 | 128.4 [113.5, 148] |
| anxiety disorders + depressive disorders + osteoarthritis | 196.9 | 128 [116.9, 158.8] |
| gastritis and duodenitis, peptic ulcer disease + anxiety disorders + obesity | 325.4 | 127.5 [107.5, 140.4] |
| EMBI-disorders + hypertension + skin and subcutaneous diseases | 1301.1 | 127.2 [95.5, 155.5] |
| diabetes + gastritis and duodenitis, peptic ulcer disease + hypertension | 630.9 | 127.1 [108, 158.6] |
| gynecological diseases + breast cancer + obesity | 279.8 | 126.7 [102.1, 149.2] |
| atrial fibrillation and flutter + low back and neck pain | 136.2 | 126.5 [115.8, 143.5] |
| depressive disorders + headache disorders | 444.6 | 126.4 [121.3, 132.7] |
| depressive disorders + asthma + obesity | 107.7 | 126.3 [106.4, 139] |
| gynecological diseases + reproductive organ cancers + hyperlipidemia | 155.1 | 125.6 [105.3, 144.9] |
| headache disorders + obesity | 481.3 | 124.8 [100.5, 147.1] |
| diabetes + hyperlipidemia + tobacco use | 225.9 | 124.5 [98.3, 148.7] |
| low back and neck pain + asthma + obesity | 189.4 | 123.1 [108.5, 152.9] |
| EMBI-disorders + hemoglobinopathies and hemolytic anemias + sense organ diseases | 229.1 | 122.6 [111.7, 128.3] |
| atrial fibrillation and flutter + hypertension + obesity | 134.4 | 121 [104, 138.1] |
| diabetes + substance use disorders | 226 | 120.5 [109.2, 128.9] |
| substance use disorders + hypertension + skin and subcutaneous diseases | 178.1 | 118.6 [108.2, 140.3] |
| IHD + gastritis and duodenitis, peptic ulcer disease + EMBI-disorders | 141.1 | 118.2 [111, 133.7] |
| EMBI-disorders + depressive disorders + skin and subcutaneous diseases | 441.5 | 117.2 [100.8, 122.2] |
| osteoarthritis + reproductive organ cancers | 200.2 | 117.2 [102, 143.5] |
| asthma + hyperlipidemia | 597.8 | 117.1 [106.1, 123.1] |
| EMBI-disorders + anxiety disorders + depressive disorders | 606.7 | 116.7 [108.1, 131] |
| hemoglobinopathies and hemolytic anemias + tobacco use | 334.5 | 116.1 [91.8, 125.2] |
| stroke + diabetes + hyperlipidemia | 136.1 | 115 [90.3, 140.5] |
| low back and neck pain + hyperlipidemia + hypertension | 1501.3 | 114.2 [101.5, 136.8] |
| diabetes + hypertension + skin and subcutaneous diseases | 1425.1 | 114.2 [102, 132] |
| gastritis and duodenitis, peptic ulcer disease + hypertension + tobacco use | 166.9 | 113.7 [86.6, 123.1] |
| diabetes + gastritis and duodenitis, peptic ulcer disease + depressive disorders | 130.7 | 113 [87.2, 130.6] |
| depressive disorders + osteoarthritis + hypertension | 185.3 | 112.7 [91.6, 118.3] |
| osteoarthritis + hyperlipidemia + skin and subcutaneous diseases | 452.3 | 112.7 [103.3, 130.1] |
| gastritis and duodenitis, peptic ulcer disease + EMBI-disorders + anxiety disorders | 348.3 | 111.7 [106.4, 117.9] |
| hypertension + obesity + tobacco use | 245.7 | 111.4 [86.9, 130.6] |
| EMBI-disorders + hypertension | 3838.9 | 111 [102.6, 121.4] |
| EMBI-disorders + gynecological diseases | 2849.3 | 110.2 [92, 119.3] |
| gastritis and duodenitis, peptic ulcer disease + depressive disorders + low back and neck pain | 254.6 | 110.2 [98.2, 127] |
| low back and neck pain + headache disorders | 1110.2 | 109.8 [85.3, 127.4] |
| diabetes + anxiety disorders + skin and subcutaneous diseases | 341.1 | 109.4 [88.6, 131.6] |
| COPD + hyperlipidemia | 278.7 | 109 [102, 130] |
| asthma + skin and subcutaneous diseases | 965.6 | 108.7 [97.3, 116.6] |
| EMBI-disorders + COPD + hypertension | 129.2 | 108.4 [83.3, 115.5] |
| osteoarthritis + hypertension + tobacco use | 100.5 | 107.8 [82.1, 132.7] |
| EMBI-disorders + hemoglobinopathies and hemolytic anemias + tobacco use | 113.6 | 107.3 [96, 114.1] |
| anxiety disorders + depressive disorders + obesity | 559.5 | 106.7 [89.4, 119.3] |
| low back and neck pain + COPD | 204.4 | 106.1 [86.4, 126.3] |
| chronic kidney disease + diabetes + hypertension | 473.1 | 105.7 [82.2, 128.8] |
| hyperlipidemia + obesity + tobacco use | 170.8 | 104.6 [95.3, 117.4] |
| anxiety disorders + low back and neck pain | 2418.3 | 103 [98.5, 118.7] |
| osteoarthritis + hyperlipidemia + sense organ diseases | 216.6 | 101.3 [82.3, 115.1] |
| substance use disorders + headache disorders | 114.1 | 101 [80.5, 112.4] |
| hypertension + sense organ diseases | 2130.8 | 100.7 [94.9, 117.5] |
| low back and neck pain + hyperlipidemia + obesity | 714.5 | 98.8 [93.2, 111.9] |
| depressive disorders + osteoarthritis + hyperlipidemia | 143.4 | 98.4 [91, 103.7] |
| gynecological diseases + substance use disorders + depressive disorders | 100.5 | 97.9 [91.6, 113.9] |
| gynecological diseases + depressive disorders + breast cancer | 154.3 | 97.7 [79, 115.3] |
| low back and neck pain + hyperlipidemia + sense organ diseases | 450.9 | 97.6 [93.3, 118.9] |
| anxiety disorders + depressive disorders + breast cancer | 187.6 | 97.2 [90.4, 106.7] |
| headache disorders + sense organ diseases + skin and subcutaneous diseases | 160.3 | 97.1 [77.2, 103.2] |
| anxiety disorders + obesity | 1582.1 | 97 [86.4, 116.1] |
| anxiety disorders + asthma + obesity | 149.9 | 96.9 [90.4, 107.5] |
| reproductive organ cancers + obesity | 516.6 | 96.8 [84, 117.3] |
| asthma + COPD | 116.5 | 95.9 [92.5, 114.1] |
| gastritis and duodenitis, peptic ulcer disease + headache disorders | 392.8 | 95.6 [80.6, 116.1] |
| diabetes + obesity + tobacco use | 126 | 94.5 [71.8, 104.7] |
| osteoarthritis + obesity + skin and subcutaneous diseases | 363.1 | 94.2 [88.4, 114] |
| diabetes + hyperlipidemia + obesity | 1324 | 94.2 [84.4, 114.7] |
| chronic kidney disease + tobacco use | 105.1 | 93.9 [80.9, 103.4] |
| inguinal, femoral, and abdominal hernia + sense organ diseases | 111.8 | 93.2 [79.2, 113.3] |
| headache disorders + asthma | 200.8 | 93.2 [86.5, 110.1] |
| diabetes + gynecological diseases + reproductive organ cancers | 101 | 93.2 [77.5, 111.5] |
| hyperlipidemia + obesity | 2964.6 | 92.9 [85.4, 108.8] |
| osteoarthritis + low back and neck pain + hypertension | 544.5 | 92.4 [81.7, 109.4] |
| substance use disorders + hypertension + tobacco use | 138 | 90.9 [85.5, 100.5] |
| anxiety disorders + osteoarthritis + hyperlipidemia | 190 | 90.9 [81.9, 110.5] |
| diabetes + hemoglobinopathies and hemolytic anemias + depressive disorders | 110.8 | 90.9 [68.4, 109.8] |
| gynecological diseases + asthma + obesity | 189.3 | 90.6 [79.9, 103.3] |
| breast cancer + headache disorders | 240.4 | 90.4 [70.6, 102.7] |
| reproductive organ cancers + hypertension | 1003.9 | 90.3 [70.6, 111.3] |
| EMBI-disorders + gout | 161.4 | 89.6 [69.3, 106] |
| diabetes + gastritis and duodenitis, peptic ulcer disease + obesity | 360 | 89.6 [82.2, 111.9] |
| anxiety disorders + hypertension + obesity | 665.9 | 89.5 [85, 105.8] |
| anxiety disorders + depressive disorders + low back and neck pain | 778.1 | 88.8 [75.2, 97.5] |
| asthma + hyperlipidemia + obesity | 194.3 | 88.6 [72.8, 108.3] |
| skin cancers + sense organ diseases | 107.8 | 87.2 [83.6, 93.1] |
| diabetes + cirrhosis | 421.4 | 87.2 [66.9, 104.1] |
| anxiety disorders + hypertension + sense organ diseases | 286.8 | 87 [70.7, 98] |
| depressive disorders + hyperlipidemia + obesity | 333.9 | 86.4 [71.7, 105.5] |
| low back and neck pain + asthma + hypertension | 239.8 | 86.4 [74.3, 99.7] |
| depressive disorders + asthma + hypertension | 113.3 | 86.1 [71.4, 90.7] |
| gastritis and duodenitis, peptic ulcer disease + depressive disorders + osteoarthritis | 101.1 | 86.1 [72.5, 99.6] |
| anxiety disorders + depressive disorders + skin and subcutaneous diseases | 901.2 | 85.5 [69.2, 97] |
| IHD + diabetes + gynecological diseases | 101.6 | 85.4 [79.3, 100.3] |
| gynecological diseases + anxiety disorders + hypertension | 497 | 84.9 [75.1, 88.6] |
| hemoglobinopathies and hemolytic anemias + anxiety disorders + sense organ diseases | 117.4 | 84.6 [64.2, 88.7] |
| reproductive organ cancers + hyperlipidemia | 859.1 | 84.1 [74.1, 93.6] |
| reproductive organ cancers + tobacco use | 106.1 | 81.3 [62.1, 97.8] |
| stroke + low back and neck pain + hypertension | 118.4 | 80.6 [65.4, 95] |
| stroke + hyperlipidemia | 334.2 | 80.5 [63.5, 97.1] |
| breast cancer + asthma | 234.7 | 80.1 [73.4, 84.4] |
| EMBI-disorders + anxiety disorders + sense organ diseases | 236.2 | 80.1 [75.8, 97.9] |
| gynecological diseases + depressive disorders | 1342.7 | 80 [62, 86.9] |
| IHD + gynecological diseases + hypertension | 187.6 | 79.7 [71.2, 97.2] |
| anxiety disorders + bipolar disorder + low back and neck pain | 104.9 | 79.5 [63.5, 89.2] |
| bipolar disorder + low back and neck pain | 179 | 79.3 [63.2, 83.9] |
| inguinal, femoral, and abdominal hernia + gastritis and duodenitis, peptic ulcer disease + EMBI-disorders | 129.1 | 79.1 [65.3, 85.2] |
| diabetes + EMBI-disorders + depressive disorders | 241.9 | 78.7 [65.9, 94.6] |
| diabetes + COPD + hypertension | 126.6 | 78.1 [73.8, 92] |
| gastritis and duodenitis, peptic ulcer disease + colon and rectum cancer | 477.1 | 77.7 [60.5, 92.2] |
| bipolar disorder + skin and subcutaneous diseases | 207.5 | 77.7 [60.8, 94.8] |
| low back and neck pain + asthma + hyperlipidemia | 174.7 | 77.2 [65.2, 82.5] |
| gastritis and biliary diseases + hyperlipidemia + hypertension | 112.2 | 76.4 [72, 82.7] |
| anxiety disorders + osteoarthritis + obesity | 172.9 | 76.3 [64.1, 79.2] |
| anxiety disorders + depressive disorders + headache disorders | 273.1 | 75.6 [66, 90.7] |
| gout + hyperlipidemia | 307.6 | 75.2 [69.5, 82.6] |
| IHD + anxiety disorders + depressive disorders | 103.2 | 74.1 [61.3, 78.8] |
| diabetes + cirrhosis + skin and subcutaneous diseases | 156.9 | 74 [68.5, 86.5] |
| gastritis and duodenitis, peptic ulcer disease + substance use disorders + hypertension | 120.9 | 74 [58.6, 86.2] |
| skin cancers + hypertension | 216.4 | 73.9 [55.6, 76.3] |
| anxiety disorders + depressive disorders + hypertension | 681 | 73.6 [62.9, 86.1] |
| gynecological diseases + anxiety disorders + reproductive organ cancers | 171.8 | 72.5 [57.1, 75.6] |
| diabetes + gastritis and duodenitis, peptic ulcer disease + anxiety disorders | 182.1 | 71.8 [59, 82.4] |
| gout + hyperlipidemia + hypertension | 213.8 | 71.4 [60.9, 82.7] |
| depressive disorders + low back and neck pain + asthma | 115.2 | 71.4 [58.5, 75.1] |
| low back and neck pain + skin cancers | 154.5 | 70.9 [68.1, 75.8] |
| depressive disorders + hypertension + sense organ diseases | 195 | 70.2 [53.8, 86.2] |
| anxiety disorders + bipolar disorder + skin and subcutaneous diseases | 114.8 | 69.4 [53.5, 77.8] |
| IHD + diabetes + obesity | 275.7 | 68.8 [61.3, 85.4] |
| colon and rectum cancer + tobacco use | 115.2 | 68.6 [63.6, 74.2] |
| tobacco use + sense organ diseases | 205.6 | 68.5 [63.8, 79.9] |
| headache disorders + hypertension + sense organ diseases | 107.6 | 68.4 [51.3, 70.9] |
| gastritis and duodenitis, peptic ulcer disease + gynecological diseases + depressive disorders | 212.6 | 68 [63.4, 74.9] |
| cirrhosis + breast cancer | 131.7 | 67.8 [60, 74.6] |
| bipolar disorder + obesity | 135.3 | 67.7 [53.1, 74.1] |
| depressive disorders + hyperlipidemia + hypertension | 584.4 | 67.2 [56, 73.9] |
| chronic kidney disease + obesity | 396.9 | 66.9 [55.7, 73.2] |
| EMBI-disorders + depressive disorders + hypertension | 422.3 | 66.8 [58.6, 81.3] |
| reproductive organ cancers + asthma | 131.8 | 66.2 [55.6, 77.5] |
| osteoarthritis + obesity + sense organ diseases | 153 | 66.1 [50.2, 73.3] |
| EMBI-disorders + depressive disorders + sense organ diseases | 158.8 | 66.1 [53.9, 71.6] |
| headache disorders + tobacco use | 124.1 | 66.1 [61, 75.5] |
| gastritis and duodenitis, peptic ulcer disease + headache disorders + obesity | 110.4 | 66 [56.3, 71] |
| COPD + hyperlipidemia + hypertension | 188.7 | 66 [62.2, 76.4] |
| substance use disorders + hyperlipidemia | 344.8 | 65.8 [57.2, 74] |
| hypertension + obesity | 4290.8 | 65.3 [60.8, 74.8] |
| gastritis and duodenitis, peptic ulcer disease + skin and subcutaneous diseases | 1972.8 | 64.7 [61.1, 74] |
| gout + skin and subcutaneous diseases | 206.3 | 64.7 [51.3, 78.1] |
| headache disorders + hypertension | 626.3 | 64.6 [58.1, 77.6] |
| EMBI-disorders + colon and rectum cancer | 640.7 | 64.3 [58.5, 71] |
| depressive disorders + low back and neck pain + tobacco use | 127.4 | 63 [52.1, 72.9] |
| EMBI-disorders + reproductive organ cancers | 622.2 | 62.6 [55.2, 69.2] |
| depressive disorders + osteoarthritis + low back and neck pain | 177.3 | 62.5 [54.6, 72.3] |
| headache disorders + hyperlipidemia | 460.1 | 62.5 [48.1, 71.2] |
| diabetes + gynecological diseases + hemoglobinopathies and hemolytic anemias | 221.8 | 61.5 [50.6, 64.3] |
| COPD + obesity | 165 | 61.3 [57.3, 74] |
| gastritis and duodenitis, peptic ulcer disease + gynecological diseases + obesity | 368.5 | 61.1 [51.2, 72.5] |
| low back and neck pain + hypertension + obesity | 1007 | 60.2 [54, 66] |
| diabetes + anxiety disorders + hypertension | 534.4 | 59.9 [53.2, 69.5] |
| diabetes + osteoarthritis + hyperlipidemia | 412.4 | 59.4 [52.1, 65] |
| diabetes + depressive disorders | 680.5 | 59.4 [48.1, 66.9] |
| gastritis and duodenitis, peptic ulcer disease + hyperlipidemia + hypertension | 1022.9 | 59.1 [56.1, 73.4] |
| gynecological diseases + anxiety disorders + bipolar disorder | 101.9 | 58.5 [50.7, 64.1] |
| diabetes + obesity | 2381.4 | 58.5 [46.5, 70.6] |
| diabetes + gastritis and duodenitis, peptic ulcer disease + hemoglobinopathies and hemolytic anemias | 206 | 58.2 [51.5, 71.8] |
| IHD + COPD + hypertension | 109.8 | 57.4 [48.5, 59.6] |
| diabetes + depressive disorders + skin and subcutaneous diseases | 244 | 57 [44.4, 65.4] |
| gynecological diseases + hemoglobinopathies and hemolytic anemias + anxiety disorders | 300.2 | 56.8 [50.8, 70.1] |
| gout + low back and neck pain | 140 | 56.4 [49.4, 66] |
| diabetes + osteoarthritis + obesity | 288 | 56.1 [44.4, 66.2] |
| diabetes + hemoglobinopathies and hemolytic anemias + hypertension | 589.8 | 56 [45.2, 69.3] |
| EMBI-disorders + anxiety disorders + tobacco use | 143.5 | 55.4 [48.4, 61.4] |
| diabetes + hyperlipidemia + hypertension | 3069.1 | 54.6 [48.5, 59.4] |
| EMBI-disorders + depressive disorders + tobacco use | 104.3 | 53.8 [48.3, 65.2] |
| diabetes + COPD + hyperlipidemia | 107.9 | 53.7 [46.4, 56.9] |
| diabetes + hypertension + obesity | 1571.1 | 52.9 [47.5, 62.2] |
| anxiety disorders + colon and rectum cancer | 320.7 | 52.8 [45.4, 57.8] |
| diabetes + gastritis and duodenitis, peptic ulcer disease + osteoarthritis | 136.9 | 52 [39.8, 57] |
| gastritis and duodenitis, peptic ulcer disease + low back and neck pain + obesity | 416.9 | 51.9 [46.7, 54.7] |
| EMBI-disorders + gynecological diseases + depressive disorders | 350.9 | 51.8 [41.7, 63.4] |
| osteoarthritis + headache disorders | 176.9 | 51.4 [42.9, 57.7] |
| osteoarthritis + breast cancer + obesity | 106 | 50.6 [45.8, 57.4] |
| rheumarthritis + skin and subcutaneous diseases | 108.1 | 49.9 [43.2, 61.8] |
| gastritis and duodenitis, peptic ulcer disease + Attention-deficit / hyperactivity disorder | 128.9 | 49.6 [40.4, 52.3] |
| low back and neck pain + hypertension + sense organ diseases | 516 | 49.4 [42.4, 58.7] |
| diabetes + depressive disorders + hypertension | 394.1 | 49.2 [45.5, 53.1] |
| EMBI-disorders + low back and neck pain + tobacco use | 147.1 | 49 [40.7, 59.5] |
| gastritis and duodenitis, peptic ulcer disease + anxiety disorders + osteoarthritis | 134.8 | 49 [45.3, 56.3] |
| EMBI-disorders + asthma | 619.6 | 48.5 [41.9, 54.1] |
| gynecological diseases + headache disorders | 827.5 | 48.5 [46.5, 58.7] |
| IHD + diabetes + gastritis and duodenitis, peptic ulcer disease | 131.6 | 48.4 [42.5, 53.1] |
| gastritis and duodenitis, peptic ulcer disease + hyperlipidemia | 1753.2 | 48.1 [39.8, 58.3] |
| chronic kidney disease + IHD + obesity | 105.5 | 48 [38.2, 59.1] |
| gastritis and duodenitis, peptic ulcer disease + hemoglobinopathies and hemolytic anemias + skin and subcutaneous diseases | 370.7 | 47.8 [42.7, 58.8] |
| anxiety disorders + headache disorders + sense organ diseases | 102.9 | 47.8 [37, 56.1] |
| sense organ diseases + skin and subcutaneous diseases | 2285 | 46.7 [40.4, 48.7] |
| depressive disorders + hypertension + skin and subcutaneous diseases | 470.8 | 46.5 [36.1, 54.3] |
| gynecological diseases + skin cancers | 125.3 | 46.4 [38.5, 49.2] |
| inguinal, femoral, and abdominal hernia + gastritis and duodenitis, peptic ulcer disease + hypertension | 183.9 | 46.1 [43.5, 56.8] |
| gastritis and duodenitis, peptic ulcer disease + osteoarthritis + sense organ diseases | 112.1 | 45.9 [35.3, 49] |
| breast cancer + obesity | 739.9 | 45 [40.7, 56.1] |
| headache disorders + skin and subcutaneous diseases | 933.8 | 44.5 [40.9, 50.7] |
| hemoglobinopathies and hemolytic anemias + tobacco use + skin and subcutaneous diseases | 114.9 | 44.2 [33.5, 51.3] |
| substance use disorders + osteoarthritis | 184.5 | 43.8 [33.2, 50.1] |
| gastritis and duodenitis, peptic ulcer disease + colon and rectum cancer + obesity | 108.1 | 43.6 [38.2, 47.3] |
| depressive disorders + headache disorders + obesity | 111 | 43.6 [35.5, 48.1] |
| cirrhosis + gastritis and duodenitis, peptic ulcer disease + gynecological diseases | 105.4 | 43.2 [37, 47.3] |
| gastritis and duodenitis, peptic ulcer disease + substance use disorders + low back and neck pain | 116.8 | 43.2 [34.7, 46] |
| depressive disorders + hyperlipidemia + sense organ diseases | 173.2 | 43.1 [34.2, 50.7] |
| gynecological diseases + low back and neck pain + asthma | 210 | 43.1 [37, 53.4] |
| depressive disorders + hypertension + obesity | 461.9 | 42.9 [34.3, 45] |
| low back and neck pain + asthma + sense organ diseases | 101.2 | 42.9 [39.8, 49] |
| cirrhosis + gynecological diseases + obesity | 111.7 | 42.3 [35.6, 51.7] |
| diabetes + gynecological diseases + depressive disorders | 158.4 | 41.9 [32.3, 51.4] |
| asthma + obesity + skin and subcutaneous diseases | 238.9 | 41.5 [36.9, 50] |
| hyperlipidemia + hypertension | 7177.3 | 40.6 [32.4, 45.8] |
| skin cancers + hyperlipidemia | 192.8 | 40.5 [32.1, 45.8] |
| anxiety disorders + hypertension | 2299.1 | 40.4 [32.7, 42.4] |
| COPD + tobacco use | 233.4 | 40.3 [38.5, 48.9] |
| diabetes + gynecological diseases + anxiety disorders | 224.6 | 40 [35.3, 46.8] |
| low back and neck pain + headache disorders + sense organ diseases | 159 | 39.5 [36.6, 41.9] |
| gynecological diseases + bipolar disorder | 171.7 | 38.3 [35.6, 39.5] |
| depressive disorders + colon and rectum cancer | 192.3 | 38.2 [31.6, 40] |
| cirrhosis + gastritis and duodenitis, peptic ulcer disease + skin and subcutaneous diseases | 155.3 | 37.5 [31.8, 43.7] |
| diabetes + obesity + skin and subcutaneous diseases | 772.8 | 37.5 [33.6, 46.2] |
| anxiety disorders + depressive disorders + sense organ diseases | 266.3 | 36.7 [32.6, 42.4] |
| Attention-deficit / hyperactivity disorder + anxiety disorders + depressive disorders | 328.2 | 36.4 [29.4, 44.6] |
| hemoglobinopathies and hemolytic anemias + depressive disorders + hypertension | 212.9 | 36.4 [27.4, 41.7] |
| low back and neck pain + obesity + sense organ diseases | 278.2 | 36.3 [32.9, 40.2] |
| anxiety disorders + low back and neck pain + tobacco use | 187.1 | 36 [27.9, 37.6] |
| gynecological diseases + depressive disorders + hypertension | 307.7 | 36 [27.9, 40.9] |
| gastritis and duodenitis, peptic ulcer disease + depressive disorders + hyperlipidemia | 224.6 | 35.7 [33.3, 37.9] |
| anxiety disorders + tobacco use + skin and subcutaneous diseases | 187.3 | 35.6 [30.9, 40.8] |
| reproductive organ cancers + hyperlipidemia + obesity | 180 | 35.4 [27.9, 39.9] |
| chronic kidney disease + gynecological diseases + hyperlipidemia | 101.6 | 35.2 [27, 43.1] |
| hypertension + skin and subcutaneous diseases | 4738.8 | 35.1 [27.6, 43] |
| breast cancer + headache disorders + skin and subcutaneous diseases | 109.3 | 35 [31.4, 41.6] |
| depressive disorders + low back and neck pain + headache disorders | 202.6 | 34.9 [26.6, 38.8] |
| chronic kidney disease + low back and neck pain | 301.3 | 34.8 [31.3, 36.5] |
| chronic kidney disease + sense organ diseases | 258.1 | 34.6 [33.3, 36.8] |
| diabetes + depressive disorders + hyperlipidemia | 363.4 | 33.9 [29.2, 35.4] |
| anxiety disorders + hypertension + skin and subcutaneous diseases | 761.2 | 33.9 [27, 37.2] |
| colon and rectum cancer + skin and subcutaneous diseases | 1000.1 | 33.5 [25.7, 36.4] |
| headache disorders + hypertension + obesity | 190.9 | 33.4 [30.6, 40.7] |
| gastritis and duodenitis, peptic ulcer disease + asthma + hypertension | 200.4 | 32.8 [26.9, 40.9] |
| depressive disorders + low back and neck pain + hyperlipidemia | 340.1 | 32.3 [30.2, 33.9] |
| gynecological diseases + headache disorders + obesity | 171.9 | 31.9 [26.5, 39.5] |
| gynecological diseases + anxiety disorders | 2421.1 | 31.9 [26.4, 37] |
| gynecological diseases + breast cancer + headache disorders | 103.8 | 31.6 [27.6, 34.6] |
| obesity + sense organ diseases | 1005.2 | 31.1 [28.8, 37.2] |
| hyperlipidemia + hypertension + sense organ diseases | 1071.5 | 31 [27.3, 34.3] |
| osteoarthritis + colon and rectum cancer | 241.6 | 30.8 [24.1, 36.5] |
| gastritis and duodenitis, peptic ulcer disease + gynecological diseases + colon and rectum cancer | 115.4 | 30.7 [29.6, 36.2] |
| gynecological diseases + colon and rectum cancer + obesity | 111.1 | 30 [26.5, 36.7] |
| reproductive organ cancers + sense organ diseases | 387.7 | 29.6 [25.3, 33.6] |
| asthma + hyperlipidemia + hypertension | 334.5 | 29.4 [25.5, 31.3] |
| depressive disorders + tobacco use + skin and subcutaneous diseases | 127 | 29.3 [24.5, 34.1] |
| osteoarthritis + low back and neck pain + sense organ diseases | 210 | 29.1 [27.6, 33] |
| gynecological diseases + depressive disorders + sense organ diseases | 141.2 | 29 [25.9, 31] |
| chronic kidney disease + EMBI-disorders + hyperlipidemia | 275.7 | 29 [22.1, 33.7] |
| hemoglobinopathies and hemolytic anemias + substance use disorders + hypertension | 103.4 | 28.9 [25.1, 33] |
| low back and neck pain + breast cancer + headache disorders | 104.4 | 28.7 [23.9, 35.7] |
| anxiety disorders + depressive disorders + reproductive organ cancers | 120 | 28.6 [22.6, 29.9] |
| anxiety disorders + colon and rectum cancer + hyperlipidemia | 107.3 | 28.5 [26.8, 30.4] |
| diabetes + osteoarthritis + hypertension | 486.5 | 28.4 [22.7, 34.5] |
| diabetes + EMBI-disorders + tobacco use | 120.1 | 28.2 [23, 31.6] |
| gout + hypertension + skin and subcutaneous diseases | 119.3 | 28.2 [24.5, 30.4] |
| EMBI-disorders + gout + hypertension | 103.9 | 28.1 [24.5, 29.7] |
| COPD + sense organ diseases | 103 | 27.8 [24.4, 34.6] |
| gastritis and duodenitis, peptic ulcer disease + EMBI-disorders + colon and rectum cancer | 131.9 | 27.7 [22.7, 32.5] |
| cirrhosis + obesity | 429.1 | 26.8 [20.4, 29.4] |
| COPD + hypertension + skin and subcutaneous diseases | 123.7 | 26.8 [23.4, 33.4] |
| anxiety disorders + breast cancer + obesity | 127.8 | 26.7 [24, 33.3] |
| reproductive organ cancers + hyperlipidemia + hypertension | 467.4 | 26.2 [23.9, 29.1] |
| gastritis and duodenitis, peptic ulcer disease + anxiety disorders + skin and subcutaneous diseases | 462.5 | 25.3 [19.9, 28.5] |
| diabetes + anxiety disorders + depressive disorders | 306.8 | 24.8 [19.3, 25.9] |
| reproductive organ cancers + hypertension + obesity | 239.1 | 24.7 [18.6, 25.6] |
| reproductive organ cancers + headache disorders | 123.5 | 24.5 [21.9, 26.1] |
| anxiety disorders + substance use disorders + hypertension | 212.4 | 23.9 [19.6, 26.3] |
| anxiety disorders + low back and neck pain + asthma | 181.1 | 23.9 [23.1, 25.9] |
| IHD + hemoglobinopathies and hemolytic anemias + low back and neck pain | 106.8 | 23.8 [18.8, 29] |
| osteoarthritis + hypertension + sense organ diseases | 261 | 23.1 [18.8, 24.6] |
| anxiety disorders + substance use disorders + obesity | 111.4 | 22.9 [18.1, 24.1] |
| colon and rectum cancer + obesity | 498.9 | 22.8 [21.2, 28.4] |
| substance use disorders + hypertension + obesity | 152 | 22.7 [19.6, 24.8] |
| Attention-deficit / hyperactivity disorder + headache disorders | 100.2 | 22.3 [17, 26.4] |
| depressive disorders + low back and neck pain + breast cancer | 122.4 | 22 [19.8, 26.6] |
| stroke + EMBI-disorders + hyperlipidemia | 127.4 | 21.7 [20.4, 22.5] |
| gynecological diseases + depressive disorders + headache disorders | 157.7 | 21.3 [16.6, 22] |
| gout + hypertension | 414.2 | 20.6 [20, 22.2] |
| EMBI-disorders + depressive disorders + breast cancer | 113.1 | 20.4 [17.8, 21.7] |
| depressive disorders + reproductive organ cancers | 230.1 | 20.3 [17.9, 25] |
| substance use disorders + low back and neck pain + skin and subcutaneous diseases | 213.8 | 19.8 [18.7, 24.5] |
| gout + obesity | 174.1 | 19.6 [16.2, 20.8] |
| substance use disorders + depressive disorders + hypertension | 164.3 | 19.4 [17.5, 23.1] |
| asthma + sense organ diseases | 320.7 | 19.1 [15.7, 20.4] |
| gastritis and duodenitis, peptic ulcer disease + reproductive organ cancers + hypertension | 129.1 | 18.9 [15.4, 22.4] |
| gynecological diseases + hemoglobinopathies and hemolytic anemias + depressive disorders | 181.6 | 18.3 [15.1, 19.9] |
| gynecological diseases + low back and neck pain + reproductive organ cancers | 213 | 18 [14.6, 21.4] |
| gastritis and duodenitis, peptic ulcer disease + low back and neck pain + hyperlipidemia | 523.9 | 17.9 [16.5, 19.1] |
| inguinal, femoral, and abdominal hernia + hyperlipidemia + skin and subcutaneous diseases | 100.4 | 17.8 [15.8, 21.8] |
| cirrhosis + EMBI-disorders + low back and neck pain | 149.4 | 17.7 [15.4, 19.5] |
| EMBI-disorders + osteoarthritis + obesity | 286.5 | 17.5 [13.2, 20.6] |
| gastritis and duodenitis, peptic ulcer disease + hemoglobinopathies and hemolytic anemias + anxiety disorders | 221.3 | 17.2 [14.6, 18.8] |
| gynecological diseases + obesity + skin and subcutaneous diseases | 776.2 | 16.8 [14.3, 20.2] |
| gout + hypertension + obesity | 120.9 | 16.6 [13.4, 17.4] |
| colon and rectum cancer + asthma | 125.4 | 16.4 [15.8, 17.4] |
| Attention-deficit / hyperactivity disorder + depressive disorders + skin and subcutaneous diseases | 138.1 | 15.3 [11.8, 17.4] |
| low back and neck pain + reproductive organ cancers | 664.9 | 14.9 [11.2, 18.3] |
| gastritis and duodenitis, peptic ulcer disease + reproductive organ cancers + hyperlipidemia | 116.3 | 14.9 [11.9, 15.7] |
| gastritis and duodenitis, peptic ulcer disease + anxiety disorders + breast cancer | 113.7 | 14.8 [13, 15.3] |
| gynecological diseases + depressive disorders + obesity | 312 | 14.5 [11.2, 15.3] |
| gynecological diseases + reproductive organ cancers + skin and subcutaneous diseases | 345 | 14.5 [14, 17.8] |
| depressive disorders + sense organ diseases + skin and subcutaneous diseases | 222.7 | 14.5 [11.1, 16.2] |
| depressive disorders + low back and neck pain + hypertension | 431.9 | 14.5 [13, 17.1] |
| diabetes + depressive disorders + sense organ diseases | 121.5 | 14.2 [13.5, 17.6] |
| osteoarthritis + asthma + skin and subcutaneous diseases | 106.8 | 14.1 [11.3, 15.4] |
| gastritis and duodenitis, peptic ulcer disease + breast cancer + obesity | 132.9 | 14 [12.8, 17.5] |
| anxiety disorders + reproductive organ cancers | 435.6 | 13.5 [12.1, 14.9] |
| gastritis and duodenitis, peptic ulcer disease + EMBI-disorders + tobacco use | 103.7 | 13.4 [12.1, 15.7] |
| gynecological diseases + asthma + skin and subcutaneous diseases | 291.1 | 13 [11.6, 15.7] |
| colon and rectum cancer + hypertension + obesity | 275.5 | 12.8 [9.9, 15.7] |
| gynecological diseases + skin and subcutaneous diseases | 4588.7 | 12.8 [10.1, 14.9] |
| anxiety disorders + osteoarthritis + low back and neck pain | 247.9 | 12.7 [9.6, 14.1] |
| depressive disorders + hyperlipidemia | 1097.8 | 12.5 [11.7, 13.8] |
| EMBI-disorders + colon and rectum cancer + obesity | 135.6 | 12.1 [10.5, 13.4] |
| chronic kidney disease + obesity + skin and subcutaneous diseases | 153.4 | 12 [10.9, 13.9] |
| gynecological diseases + substance use disorders + low back and neck pain | 126.5 | 12 [9.5, 12.7] |
| low back and neck pain + reproductive organ cancers + hyperlipidemia | 176.8 | 12 [11.4, 13.7] |
| skin cancers + hyperlipidemia + hypertension | 104.5 | 11.6 [9.4, 12.2] |
| gastritis and duodenitis, peptic ulcer disease + reproductive organ cancers + skin and subcutaneous diseases | 121.9 | 11.6 [9.4, 13.2] |
| EMBI-disorders + gynecological diseases + anxiety disorders | 586.5 | 11.3 [8.5, 13.5] |
| depressive disorders + breast cancer + skin and subcutaneous diseases | 165.1 | 11.2 [8.9, 13.1] |
| Attention-deficit / hyperactivity disorder + depressive disorders | 506.1 | 11 [9.8, 11.5] |
| osteoarthritis + low back and neck pain + breast cancer | 140.3 | 10.9 [9.5, 11.4] |
| IHD + sense organ diseases | 343 | 10.3 [9, 12.2] |
| anxiety disorders + hyperlipidemia + obesity | 458.5 | 10.3 [8, 12.3] |
| hypertension + tobacco use + skin and subcutaneous diseases | 281.9 | 10.2 [8.2, 12] |
| gynecological diseases + headache disorders + hyperlipidemia | 130.6 | 9.9 [9.6, 11.7] |
| anxiety disorders + asthma + hypertension | 167.1 | 9.6 [8.3, 10] |
| depressive disorders + breast cancer + hyperlipidemia | 110.7 | 9.5 [7.4, 10.7] |
| gynecological diseases + osteoarthritis + hyperlipidemia | 209.5 | 8.9 [8.1, 10.3] |
| gastritis and duodenitis, peptic ulcer disease + anxiety disorders + asthma | 133.7 | 8.5 [7, 10.6] |
| stroke + low back and neck pain + skin and subcutaneous diseases | 105.7 | 8.4 [7.3, 8.9] |
| gastritis and duodenitis, peptic ulcer disease + colon and rectum cancer + hyperlipidemia | 179.8 | 8.4 [7.6, 9.6] |
| gastritis and duodenitis, peptic ulcer disease + low back and neck pain + hypertension | 641.1 | 8.2 [6.9, 10.1] |
| diabetes + substance use disorders + hypertension | 146.9 | 8.2 [7.3, 8.8] |
| diabetes + reproductive organ cancers + obesity | 134.5 | 8.1 [7.8, 9.7] |
| EMBI-disorders + reproductive organ cancers + obesity | 125.2 | 8 [7.7, 9] |
| low back and neck pain + skin cancers + skin and subcutaneous diseases | 131.3 | 7.5 [6.1, 8.9] |
| depressive disorders + osteoarthritis + skin and subcutaneous diseases | 168.3 | 7.3 [6.8, 7.9] |
| EMBI-disorders + obesity + tobacco use | 122.6 | 6.5 [6, 7.7] |
| stroke + obesity | 152.9 | 6.2 [5.4, 6.5] |
| depressive disorders + low back and neck pain + skin and subcutaneous diseases | 536.8 | 6.1 [4.9, 6.9] |
| osteoarthritis + breast cancer | 384.9 | 6 [4.9, 6.5] |
| EMBI-disorders + gynecological diseases + tobacco use | 103.6 | 5.8 [4.6, 6.7] |
| low back and neck pain + tobacco use + skin and subcutaneous diseases | 214.2 | 5.6 [4.6, 6.1] |
| gynecological diseases + anxiety disorders + depressive disorders | 766.1 | 5.2 [4.8, 5.4] |
| diabetes + headache disorders | 242.2 | 5.1 [4.1, 5.4] |
| diabetes + low back and neck pain | 1697.4 | 5.1 [3.9, 5.3] |
| EMBI-disorders + substance use disorders + hypertension | 186.8 | 4.3 [3.3, 5] |
| depressive disorders + breast cancer + hypertension | 123 | 4.2 [4, 5.3] |
| IHD + reproductive organ cancers | 125 | 4.2 [3.7, 5.1] |
| breast cancer + obesity + skin and subcutaneous diseases | 283 | 4 [3.4, 4.1] |
| anxiety disorders + breast cancer + skin and subcutaneous diseases | 295.1 | 3.7 [3.2, 4.3] |
| diabetes + asthma | 406 | 3.5 [3, 3.7] |
| anxiety disorders + osteoarthritis + hypertension | 248.4 | 3.5 [3.2, 3.6] |
| diabetes + tobacco use + skin and subcutaneous diseases | 133.1 | 3.1 [2.4, 3.4] |
| depressive disorders + obesity + skin and subcutaneous diseases | 371.1 | 2.4 [2.1, 2.9] |
| gastritis and duodenitis, peptic ulcer disease + obesity + skin and subcutaneous diseases | 493.9 | 2.4 [2.3, 2.7] |
| anxiety disorders + headache disorders | 736.9 | 2.1 [1.9, 2.4] |
| anxiety disorders + skin and subcutaneous diseases | 3032.6 | 2 [1.5, 2.5] |
| low back and neck pain + oral disorders | 146.5 | 1.7 [1.6, 2.1] |
| gynecological diseases + colon and rectum cancer + hyperlipidemia | 171 | 1.1 [0.9, 1.4] |
| low back and neck pain + breast cancer + hyperlipidemia | 256.5 | 0.9 [0.9, 1.1] |
| gynecological diseases + anxiety disorders + asthma | 178 | 0.5 [0.4, 0.5] |
| gynecological diseases + breast cancer + hypertension | 432.2 | 0.4 [0.3, 0.5] |
| breast cancer + hyperlipidemia + obesity | 250.9 | 0.1 [0.1, 0.1] |
| EMBI-disorders + headache disorders + sense organ diseases | 104.2 | 0 [0, 0] |
| anxiety disorders + headache disorders + obesity | 156.7 | -0.1 [-0.1, -0.1] |
| gynecological diseases + depressive disorders + skin and subcutaneous diseases | 515.3 | -0.7 [-0.7, -0.6] |
| gastritis and duodenitis, peptic ulcer disease + low back and neck pain + sense organ diseases | 246.3 | -1.2 [-1.4, -1] |
| gastritis and duodenitis, peptic ulcer disease + asthma + hyperlipidemia | 151.6 | -1.2 [-1.4, -0.9] |
| diabetes + hyperlipidemia + sense organ diseases | 806.4 | -1.4 [-1.6, -1.1] |
| IHD + gastritis and duodenitis, peptic ulcer disease + hypertension | 263.6 | -1.7 [-1.8, -1.4] |
| inflammatory bowel disease + hyperlipidemia | 181 | -1.8 [-2, -1.6] |
| anxiety disorders + breast cancer + hyperlipidemia | 170.1 | -2 [-2.1, -1.7] |
| Attention-deficit / hyperactivity disorder + depressive disorders + low back and neck pain | 113.7 | -2 [-2.2, -1.9] |
| low back and neck pain + colon and rectum cancer | 600.9 | -2 [-2.5, -1.9] |
| low back and neck pain + headache disorders + obesity | 205.6 | -2.1 [-2.4, -1.8] |
| gynecological diseases + anxiety disorders + hyperlipidemia | 371.7 | -2.7 [-2.8, -2.3] |
| hemoglobinopathies and hemolytic anemias + COPD + hypertension | 110.9 | -2.7 [-3.2, -2.4] |
| bipolar disorder + hypertension | 159.5 | -2.7 [-3.2, -2.4] |
| hemoglobinopathies and hemolytic anemias + colon and rectum cancer + hypertension | 120.3 | -2.8 [-3.1, -2.4] |
| gynecological diseases + low back and neck pain + headache disorders | 359.5 | -3 [-3.6, -2.4] |
| depressive disorders + headache disorders + hypertension | 120.7 | -3 [-3.6, -2.6] |
| Attention-deficit / hyperactivity disorder + hyperlipidemia | 179.6 | -3.5 [-3.6, -2.8] |
| EMBI-disorders + skin cancers + skin and subcutaneous diseases | 128.7 | -3.7 [-3.9, -2.8] |
| diabetes + osteoarthritis | 714.9 | -4 [-4.8, -3.2] |
| gastritis and duodenitis, peptic ulcer disease + headache disorders + hypertension | 139.6 | -4.1 [-5, -3.7] |
| colon and rectum cancer + reproductive organ cancers + hyperlipidemia | 105.7 | -4.3 [-5.2, -3.4] |
| gynecological diseases + asthma + hyperlipidemia | 133.5 | -4.4 [-4.6, -3.7] |
| colon and rectum cancer + hyperlipidemia + obesity | 212.4 | -4.8 [-5.6, -4.3] |
| diabetes + reproductive organ cancers | 503.6 | -5 [-5.9, -3.9] |
| osteoarthritis + low back and neck pain + skin and subcutaneous diseases | 527.2 | -5.1 [-6.3, -4.3] |
| Attention-deficit / hyperactivity disorder + anxiety disorders + low back and neck pain | 169.4 | -5.3 [-5.5, -4.9] |
| Attention-deficit / hyperactivity disorder + low back and neck pain | 361.7 | -5.7 [-6.1, -5.2] |
| reproductive organ cancers + obesity + skin and subcutaneous diseases | 169.2 | -5.7 [-6.2, -4.5] |
| gastritis and duodenitis, peptic ulcer disease + tobacco use + skin and subcutaneous diseases | 120.5 | -6.1 [-7.6, -5.8] |
| headache disorders + hyperlipidemia + obesity | 125.4 | -6.2 [-7.5, -5.6] |
| gastritis and duodenitis, peptic ulcer disease + low back and neck pain + tobacco use | 117.9 | -6.3 [-6.6, -5.7] |
| asthma + hyperlipidemia + sense organ diseases | 106.2 | -6.5 [-7.4, -4.9] |
| gastritis and duodenitis, peptic ulcer disease + hyperlipidemia + tobacco use | 125 | -6.5 [-7.7, -5.5] |
| diabetes + gynecological diseases + sense organ diseases | 236.3 | -6.5 [-7.7, -6.2] |
| anxiety disorders + breast cancer + hypertension | 196.3 | -6.6 [-7.6, -5.2] |
| hyperlipidemia + obesity + sense organ diseases | 442.8 | -7 [-7.4, -6.3] |
| gastritis and duodenitis, peptic ulcer disease + EMBI-disorders + skin and subcutaneous diseases | 598.8 | -7.1 [-7.7, -6] |
| low back and neck pain + asthma + skin and subcutaneous diseases | 293.9 | -8.3 [-9.9, -7.9] |
| EMBI-disorders + hemoglobinopathies and hemolytic anemias + low back and neck pain | 413 | -8.4 [-9.1, -7.1] |
| gynecological diseases + depressive disorders + hyperlipidemia | 241.5 | -9.1 [-11.1, -7.9] |
| breast cancer + tobacco use | 129.7 | -9.6 [-11.6, -7.4] |
| diabetes + gynecological diseases | 1327.5 | -10 [-10.9, -9] |
| gynecological diseases + headache disorders + hypertension | 180 | -10 [-11.3, -7.6] |
| gynecological diseases + osteoarthritis + sense organ diseases | 127.4 | -10 [-10.6, -8.1] |
| osteoarthritis + breast cancer + hypertension | 153 | -10.6 [-12.2, -9.1] |
| depressive disorders + headache disorders + skin and subcutaneous diseases | 169.8 | -11.3 [-12.9, -10] |
| gynecological diseases + hemoglobinopathies and hemolytic anemias + obesity | 335.9 | -11.4 [-14.1, -9.4] |
| low back and neck pain + colon and rectum cancer + hyperlipidemia | 197.8 | -11.5 [-12.4, -10] |
| colon and rectum cancer + reproductive organ cancers + skin and subcutaneous diseases | 105.5 | -11.6 [-13.5, -9.2] |
| depressive disorders + asthma + skin and subcutaneous diseases | 132.4 | -11.6 [-13.3, -8.7] |
| depressive disorders + low back and neck pain + obesity | 333 | -11.9 [-12.8, -11] |
| gynecological diseases + anxiety disorders + sense organ diseases | 235.7 | -12.1 [-14.5, -10.8] |
| substance use disorders + hyperlipidemia + hypertension | 220.8 | -12.1 [-14.8, -9.7] |
| IHD + colon and rectum cancer | 139.2 | -12.3 [-13, -10.7] |
| gastritis and duodenitis, peptic ulcer disease + EMBI-disorders + hypertension | 643.6 | -12.3 [-13.6, -10.5] |
| gynecological diseases + skin cancers + skin and subcutaneous diseases | 105.9 | -12.9 [-15.6, -11.5] |
| colon and rectum cancer + obesity + skin and subcutaneous diseases | 171.4 | -13.1 [-14.5, -11.5] |
| colon and rectum cancer + reproductive organ cancers | 326.8 | -13.2 [-16, -10.8] |
| headache disorders + hyperlipidemia + hypertension | 221.1 | -13.3 [-14, -10.1] |
| breast cancer + asthma + skin and subcutaneous diseases | 104.6 | -13.5 [-14.7, -11.5] |
| breast cancer + skin and subcutaneous diseases | 1807.8 | -14.1 [-16.7, -12.8] |
| diabetes + low back and neck pain + sense organ diseases | 315.3 | -14.2 [-17.3, -12.6] |
| hypertension + tobacco use + sense organ diseases | 100.6 | -14.8 [-15.6, -12.1] |
| colon and rectum cancer + reproductive organ cancers + hypertension | 115.8 | -15 [-18.2, -13.4] |
| diabetes + low back and neck pain + asthma | 115.8 | -15 [-15.4, -12.3] |
| anxiety disorders + low back and neck pain + sense organ diseases | 276.6 | -15.5 [-18.5, -12.1] |
| depressive disorders + low back and neck pain + sense organ diseases | 176.8 | -15.5 [-16.6, -14.5] |
| diabetes + asthma + obesity | 165.5 | -15.6 [-18.9, -13.8] |
| IHD + depressive disorders + hypertension | 140 | -16.6 [-19.9, -15.5] |
| anxiety disorders + low back and neck pain + reproductive organ cancers | 110.1 | -17 [-18.5, -15.5] |
| diabetes + depressive disorders + obesity | 254 | -17 [-20.8, -14.2] |
| EMBI-disorders + osteoarthritis + hyperlipidemia | 368.5 | -17.1 [-21, -16.3] |
| diabetes + gynecological diseases + obesity | 459.5 | -17.2 [-18.4, -14.4] |
| depressive disorders + obesity + sense organ diseases | 126.1 | -17.3 [-20.7, -16] |
| gynecological diseases + asthma + hypertension | 190.4 | -17.4 [-18.9, -13.6] |
| EMBI-disorders + bipolar disorder | 155.1 | -17.5 [-19.6, -14.7] |
| IHD + anxiety disorders + obesity | 101.8 | -17.7 [-19.3, -17] |
| gastritis and duodenitis, peptic ulcer disease + gynecological diseases + breast cancer | 221.6 | -17.9 [-21.5, -16.9] |
| low back and neck pain + breast cancer + sense organ diseases | 178.2 | -18.7 [-21.9, -17.6] |
| gastritis and duodenitis, peptic ulcer disease + anxiety disorders + headache disorders | 139.9 | -19.9 [-20.7, -16.5] |
| diabetes + gastritis and duodenitis, peptic ulcer disease + gynecological diseases | 222.5 | -20.3 [-24, -17.7] |
| diabetes + headache disorders + hyperlipidemia | 119.3 | -20.4 [-23.9, -15.7] |
| anxiety disorders + hyperlipidemia + sense organ diseases | 246.8 | -20.5 [-22.8, -19] |
| diabetes + depressive disorders + low back and neck pain | 205.1 | -20.8 [-24.9, -18.5] |
| diabetes + anxiety disorders + osteoarthritis | 107.8 | -20.9 [-22.9, -17.8] |
| breast cancer + hyperlipidemia + sense organ diseases | 205 | -21.2 [-21.8, -16.5] |
| anxiety disorders + colon and rectum cancer + hypertension | 121.1 | -21.3 [-23.6, -16.4] |
| low back and neck pain + reproductive organ cancers + obesity | 114.7 | -21.7 [-24.1, -18.9] |
| stroke + hypertension + obesity | 112.7 | -21.9 [-26.5, -19.8] |
| gynecological diseases + low back and neck pain + obesity | 566.7 | -22.1 [-24.6, -17.1] |
| diabetes + low back and neck pain + obesity | 546.3 | -22.5 [-23.3, -21.5] |
| reproductive organ cancers + hyperlipidemia + sense organ diseases | 135.2 | -22.5 [-23.7, -19.7] |
| low back and neck pain + obesity + tobacco use | 134.4 | -22.8 [-25.5, -22] |
| diabetes + anxiety disorders + obesity | 327.8 | -22.9 [-24.6, -17.4] |
| gastritis and duodenitis, peptic ulcer disease + low back and neck pain + asthma | 167.8 | -22.9 [-25.2, -21.1] |
| osteoarthritis + breast cancer + hyperlipidemia | 119.3 | -23 [-25.9, -19.1] |
| low back and neck pain + sense organ diseases + skin and subcutaneous diseases | 637.1 | -23.1 [-24.1, -21.8] |
| Attention-deficit / hyperactivity disorder + anxiety disorders + hypertension | 110.7 | -23.1 [-27.9, -19.9] |
| diabetes + gynecological diseases + skin and subcutaneous diseases | 506.8 | -23.6 [-27.9, -18.5] |
| hemoglobinopathies and hemolytic anemias + colon and rectum cancer + hyperlipidemia | 100.7 | -24.3 [-26, -22.4] |
| oral disorders + hyperlipidemia | 106.1 | -24.3 [-26.5, -23.5] |
| Attention-deficit / hyperactivity disorder + low back and neck pain + skin and subcutaneous diseases | 123.1 | -24.5 [-29.5, -20.9] |
| headache disorders + hyperlipidemia + skin and subcutaneous diseases | 183.5 | -24.8 [-27.1, -20.5] |
| gynecological diseases + hemoglobinopathies and hemolytic anemias + breast cancer | 192.3 | -24.9 [-30.1, -19.4] |
| breast cancer + reproductive organ cancers + hypertension | 100.7 | -25.6 [-27.1, -19.8] |
| colon and rectum cancer + hyperlipidemia | 926.9 | -25.9 [-27.1, -23.9] |
| gynecological diseases + hyperlipidemia + obesity | 531.2 | -25.9 [-28.2, -21.7] |
| gynecological diseases + headache disorders + sense organ diseases | 115.3 | -26.8 [-30.3, -25.7] |
| diabetes + EMBI-disorders + hypertension | 1342.7 | -27 [-30.2, -22.7] |
| reproductive organ cancers + hyperlipidemia + skin and subcutaneous diseases | 280.8 | -27 [-29.5, -21.4] |
| low back and neck pain + colon and rectum cancer + obesity | 116.8 | -27.1 [-30.8, -25.1] |
| diabetes + colon and rectum cancer + hyperlipidemia | 313.4 | -27.6 [-32.9, -21.3] |
| anxiety disorders + reproductive organ cancers + skin and subcutaneous diseases | 156.6 | -27.8 [-34.4, -24.8] |
| low back and neck pain + headache disorders + hyperlipidemia | 201.4 | -27.8 [-34.4, -23.8] |
| low back and neck pain + breast cancer + obesity | 191.5 | -28.1 [-33.3, -21.6] |
| EMBI-disorders + anxiety disorders + breast cancer | 185.9 | -28.2 [-34.3, -22.2] |
| IHD + gastritis and duodenitis, peptic ulcer disease + skin and subcutaneous diseases | 145.2 | -28.3 [-33.5, -25.6] |
| gastritis and duodenitis, peptic ulcer disease + EMBI-disorders + obesity | 415.6 | -28.3 [-29.9, -23.6] |
| low back and neck pain + colon and rectum cancer + skin and subcutaneous diseases | 248.7 | -28.3 [-30.7, -22.3] |
| anxiety disorders + asthma + hyperlipidemia | 116.9 | -28.4 [-31.6, -24.5] |
| diabetes + reproductive organ cancers + hyperlipidemia | 282.5 | -28.7 [-30.2, -23.4] |
| anxiety disorders + low back and neck pain + breast cancer | 205.3 | -28.7 [-33.2, -22.7] |
| gynecological diseases + hypertension + sense organ diseases | 375.2 | -29 [-34.7, -23.9] |
| gastritis and duodenitis, peptic ulcer disease + osteoarthritis + skin and subcutaneous diseases | 258 | -29.1 [-36.3, -23.7] |
| reproductive organ cancers + hypertension + sense organ diseases | 146.1 | -29.1 [-30, -23.8] |
| gynecological diseases + depressive disorders + osteoarthritis | 102.6 | -29.2 [-35.5, -22] |
| IHD + asthma | 130.1 | -29.4 [-32.5, -27.1] |
| hemoglobinopathies and hemolytic anemias + obesity | 1080.5 | -30 [-33.1, -27.8] |
| inguinal, femoral, and abdominal hernia + gastritis and duodenitis, peptic ulcer disease + gynecological diseases | 109.4 | -30.1 [-36.9, -27.2] |
| low back and neck pain + obesity + skin and subcutaneous diseases | 810.5 | -30.2 [-34.8, -27.7] |
| diabetes + low back and neck pain + colon and rectum cancer | 108.2 | -30.3 [-32.9, -27.2] |
| breast cancer + hypertension | 1300.9 | -30.5 [-34.2, -24.2] |
| gastritis and duodenitis, peptic ulcer disease + gynecological diseases + headache disorders | 146.5 | -30.6 [-36, -26.6] |
| peripheral vascular disease+ low back and neck pain | 131 | -31 [-38.3, -27.3] |
| hypertension + obesity + skin and subcutaneous diseases | 1315.2 | -31.1 [-35.8, -26.5] |
| gynecological diseases + Attention-deficit / hyperactivity disorder + depressive disorders | 108.2 | -31.5 [-33.3, -23.7] |
| anxiety disorders + sense organ diseases + skin and subcutaneous diseases | 366.1 | -32 [-34.8, -28.3] |
| gynecological diseases + low back and neck pain + sense organ diseases | 359.9 | -32.1 [-39.4, -29.3] |
| anxiety disorders + headache disorders + hypertension | 181.5 | -32.6 [-39.4, -25.9] |
| anxiety disorders + headache disorders + hyperlipidemia | 128.9 | -32.7 [-38.4, -25.3] |
| diabetes + gastritis and duodenitis, peptic ulcer disease + hyperlipidemia | 576.3 | -32.7 [-36.4, -30.1] |
| breast cancer + hypertension + obesity | 349.8 | -32.8 [-37.7, -27] |
| gastritis and duodenitis, peptic ulcer disease + gynecological diseases + anxiety disorders | 359.2 | -32.8 [-36.5, -30.4] |
| low back and neck pain + headache disorders + skin and subcutaneous diseases | 432 | -32.9 [-34.6, -28] |
| colon and rectum cancer + hyperlipidemia + sense organ diseases | 157.9 | -33 [-39, -29.4] |
| diabetes + EMBI-disorders + gynecological diseases | 472.7 | -33 [-38.7, -30] |
| diabetes + colon and rectum cancer + obesity | 154.7 | -33.6 [-40.1, -30.8] |
| anxiety disorders + low back and neck pain + obesity | 476.7 | -34.4 [-40.3, -28.3] |
| diabetes + gout + hyperlipidemia | 115.9 | -34.4 [-42.3, -26.8] |
| gynecological diseases + hyperlipidemia + sense organ diseases | 328.3 | -34.6 [-36.2, -28.2] |
| diabetes + EMBI-disorders + reproductive organ cancers | 127.9 | -34.9 [-36.5, -29.1] |
| breast cancer + obesity + sense organ diseases | 116.4 | -35.2 [-39.5, -27.5] |
| depressive disorders + hypertension + tobacco use | 150.3 | -35.5 [-37.1, -31.7] |
| gastritis and duodenitis, peptic ulcer disease + breast cancer | 528.3 | -35.5 [-38.9, -28.6] |
| EMBI-disorders + gynecological diseases + colon and rectum cancer | 172.2 | -37 [-44.9, -31.7] |
| gynecological diseases + anxiety disorders + obesity | 461.8 | -37.1 [-43.5, -35.3] |
| gynecological diseases + tobacco use + skin and subcutaneous diseases | 148.1 | -37.6 [-39.4, -30.1] |
| gynecological diseases + low back and neck pain + hyperlipidemia | 549.4 | -37.6 [-42.4, -29.7] |
| gastritis and duodenitis, peptic ulcer disease + low back and neck pain + headache disorders | 190.3 | -37.6 [-46.9, -36.3] |
| anxiety disorders + reproductive organ cancers + hypertension | 107.8 | -37.7 [-42.8, -36.5] |
| gynecological diseases + osteoarthritis | 676.2 | -37.8 [-45.3, -31] |
| EMBI-disorders + low back and neck pain + reproductive organ cancers | 146.4 | -37.9 [-45.5, -32.9] |
| diabetes + anxiety disorders + low back and neck pain | 281.5 | -38.1 [-46.3, -32.6] |
| diabetes + headache disorders + hypertension | 130 | -38.2 [-45.2, -29.3] |
| diabetes + sense organ diseases | 1405.9 | -38.6 [-46.4, -30.7] |
| inflammatory bowel disease + hyperlipidemia + hypertension | 105.1 | -39.5 [-44.5, -34.6] |
| gynecological diseases + hypertension + obesity | 807.4 | -39.6 [-42.4, -37.3] |
| EMBI-disorders + hyperlipidemia + tobacco use | 186.7 | -40.2 [-45.3, -32.5] |
| low back and neck pain + hypertension + tobacco use | 254.6 | -40.6 [-46.4, -30.5] |
| diabetes + colon and rectum cancer | 541.1 | -41.4 [-50.5, -31.5] |
| EMBI-disorders + anxiety disorders + asthma | 149.1 | -41.8 [-51.4, -33.7] |
| diabetes + hypertension + sense organ diseases | 873.3 | -41.8 [-46, -39.4] |
| gynecological diseases + osteoarthritis + breast cancer | 138.4 | -42.1 [-46.3, -40.5] |
| gynecological diseases + depressive disorders + asthma | 108.9 | -42.3 [-50.9, -40] |
| skin cancers + hypertension + skin and subcutaneous diseases | 181.2 | -42.4 [-48.1, -32.7] |
| EMBI-disorders + COPD + hyperlipidemia | 101.6 | -42.4 [-49.9, -36.4] |
| osteoarthritis + hypertension + skin and subcutaneous diseases | 573.1 | -42.5 [-50.1, -40.8] |
| diabetes + anxiety disorders + sense organ diseases | 156.8 | -42.8 [-47.1, -37.4] |
| skin cancers + hyperlipidemia + skin and subcutaneous diseases | 163.4 | -43 [-50.3, -32.4] |
| gynecological diseases + colon and rectum cancer + hypertension | 193.9 | -43.1 [-46.7, -35] |
| reproductive organ cancers + sense organ diseases + skin and subcutaneous diseases | 151 | -43.4 [-47, -36.8] |
| gastritis and duodenitis, peptic ulcer disease + headache disorders + hyperlipidemia | 108.1 | -43.4 [-54, -40.8] |
| Attention-deficit / hyperactivity disorder + obesity | 214.5 | -43.5 [-53.9, -37.7] |
| anxiety disorders + depressive disorders + hyperlipidemia | 511.5 | -43.6 [-49, -35.8] |
| Attention-deficit / hyperactivity disorder + hypertension | 264.8 | -43.9 [-52.7, -35.6] |
| gastritis and duodenitis, peptic ulcer disease + colon and rectum cancer + hypertension | 204 | -44 [-49.6, -39.3] |
| gynecological diseases + breast cancer + hyperlipidemia | 356.4 | -44.1 [-48.2, -35.4] |
| diabetes + reproductive organ cancers + skin and subcutaneous diseases | 155.4 | -44.1 [-50.8, -35.3] |
| anxiety disorders + hypertension + tobacco use | 219.1 | -44.2 [-50, -39.8] |
| Attention-deficit / hyperactivity disorder + skin and subcutaneous diseases | 489.9 | -44.9 [-50.5, -39.2] |
| low back and neck pain + breast cancer | 1052.1 | -45.1 [-52.2, -41.7] |
| gynecological diseases + colon and rectum cancer | 611.2 | -45.3 [-50.8, -40.6] |
| EMBI-disorders + reproductive organ cancers + hypertension | 221.1 | -45.4 [-47.2, -39.2] |
| gynecological diseases + hypertension + skin and subcutaneous diseases | 957.5 | -45.7 [-55.4, -41.8] |
| diabetes + breast cancer + hyperlipidemia | 327.5 | -46 [-53.7, -39.8] |
| diabetes + osteoarthritis + low back and neck pain | 256.6 | -46.9 [-54.2, -39.9] |
| osteoarthritis + colon and rectum cancer + skin and subcutaneous diseases | 102.1 | -47.2 [-53.7, -36] |
| EMBI-disorders + breast cancer + obesity | 216.2 | -47.3 [-56, -41.3] |
| diabetes + colon and rectum cancer + sense organ diseases | 107.1 | -47.5 [-57.9, -42.5] |
| gastritis and duodenitis, peptic ulcer disease + colon and rectum cancer + skin and subcutaneous diseases | 178.5 | -47.5 [-56.6, -36.9] |
| IHD + hemoglobinopathies and hemolytic anemias + obesity | 132.7 | -47.6 [-49.5, -37.7] |
| anxiety disorders + obesity + tobacco use | 123.6 | -47.7 [-52.1, -42.6] |
| low back and neck pain + reproductive organ cancers + hypertension | 201.2 | -47.7 [-55.3, -41.1] |
| low back and neck pain + colon and rectum cancer + sense organ diseases | 101.1 | -47.8 [-49.8, -43] |
| EMBI-disorders + gynecological diseases + asthma | 193.4 | -48.3 [-53.2, -46] |
| hemoglobinopathies and hemolytic anemias + anxiety disorders + obesity | 229.6 | -48.4 [-58.9, -44.3] |
| gynecological diseases + Attention-deficit / hyperactivity disorder + anxiety disorders | 164.7 | -48.4 [-55.6, -46.7] |
| osteoarthritis + colon and rectum cancer + hypertension | 110 | -49.1 [-54, -38.3] |
| asthma + hyperlipidemia + skin and subcutaneous diseases | 228.8 | -49.5 [-53.6, -41.2] |
| EMBI-disorders + osteoarthritis | 881.7 | -49.9 [-56.7, -42.5] |
| Attention-deficit / hyperactivity disorder + anxiety disorders + skin and subcutaneous diseases | 215 | -49.9 [-58.8, -45.8] |
| gastritis and duodenitis, peptic ulcer disease + low back and neck pain + breast cancer | 168 | -50 [-57.1, -44.6] |
| COPD + hypertension + obesity | 109.7 | -50.1 [-53.2, -47.6] |
| gastritis and duodenitis, peptic ulcer disease + breast cancer + hyperlipidemia | 177 | -50.5 [-53.2, -42.7] |
| headache disorders + obesity + skin and subcutaneous diseases | 184.7 | -50.5 [-53.5, -48.3] |
| hyperlipidemia + sense organ diseases + skin and subcutaneous diseases | 706.7 | -50.7 [-54.9, -47.8] |
| diabetes + reproductive organ cancers + hypertension | 294.7 | -50.9 [-58.5, -43.9] |
| EMBI-disorders + asthma + obesity | 190.3 | -51.2 [-57.6, -49.1] |
| gastritis and duodenitis, peptic ulcer disease + EMBI-disorders + osteoarthritis | 192.5 | -52.2 [-63.7, -42.9] |
| EMBI-disorders + colon and rectum cancer + hypertension | 263.1 | -52.6 [-55.1, -42.2] |
| EMBI-disorders + low back and neck pain | 2645.5 | -52.7 [-64.4, -44.6] |
| gynecological diseases + depressive disorders + low back and neck pain | 410.2 | -52.8 [-57.2, -44.9] |
| diabetes + low back and neck pain + headache disorders | 108.7 | -52.9 [-58.3, -43.1] |
| low back and neck pain + reproductive organ cancers + skin and subcutaneous diseases | 251.8 | -53.1 [-59, -40.9] |
| gastritis and duodenitis, peptic ulcer disease + low back and neck pain + colon and rectum cancer | 129 | -53.2 [-64.9, -51.5] |
| EMBI-disorders + depressive disorders + headache disorders | 121.5 | -53.2 [-62.4, -49.2] |
| diabetes + low back and neck pain + hyperlipidemia | 918.9 | -53.3 [-64.5, -51] |
| EMBI-disorders + gynecological diseases + breast cancer | 433.3 | -53.4 [-61.3, -51.5] |
| inguinal, femoral, and abdominal hernia + gastritis and duodenitis, peptic ulcer disease + hyperlipidemia | 141.3 | -53.5 [-64.8, -44.3] |
| diabetes + EMBI-disorders + colon and rectum cancer | 151.3 | -53.5 [-59.3, -43.8] |
| gastritis and duodenitis, peptic ulcer disease + anxiety disorders + low back and neck pain | 411.6 | -53.8 [-56.4, -41.7] |
| asthma + hypertension + sense organ diseases | 132.1 | -53.9 [-61.8, -49.1] |
| diabetes + low back and neck pain + tobacco use | 105 | -54.4 [-67.5, -51.7] |
| anxiety disorders + obesity + sense organ diseases | 168.9 | -54.8 [-60.6, -51.7] |
| diabetes + gout + hypertension | 136.8 | -55.8 [-67.9, -45] |
| gynecological diseases + anxiety disorders + osteoarthritis | 145.6 | -56.5 [-68.2, -51.4] |
| anxiety disorders + low back and neck pain + headache disorders | 328.7 | -57 [-64.9, -42.9] |
| bipolar disorder + hyperlipidemia | 123.9 | -57.2 [-63.5, -51.6] |
| anxiety disorders + breast cancer + sense organ diseases | 104.5 | -57.9 [-61.7, -52.1] |
| diabetes + breast cancer + sense organ diseases | 134.8 | -58.2 [-70.4, -44.9] |
| low back and neck pain + breast cancer + hypertension | 297.8 | -58.4 [-68.7, -51.8] |
| IHD + breast cancer | 120.7 | -58.5 [-66.6, -54.4] |
| inguinal, femoral, and abdominal hernia + gastritis and duodenitis, peptic ulcer disease + skin and subcutaneous diseases | 160.6 | -58.6 [-63.1, -55.7] |
| obesity + tobacco use + skin and subcutaneous diseases | 154.2 | -59.5 [-61.5, -47] |
| diabetes + hyperlipidemia + skin and subcutaneous diseases | 1317 | -60 [-61.9, -45.8] |
| hemoglobinopathies and hemolytic anemias + breast cancer + obesity | 106.2 | -60.8 [-64.6, -56.1] |
| breast cancer + hyperlipidemia | 1062.9 | -61.1 [-75.7, -52.8] |
| breast cancer + hyperlipidemia + skin and subcutaneous diseases | 417.2 | -61.6 [-65, -52.3] |
| cirrhosis + low back and neck pain | 362.7 | -61.7 [-64.2, -53.8] |
| diabetes + substance use disorders + hyperlipidemia | 116.8 | -62.7 [-72.2, -60.7] |
| colon and rectum cancer + hypertension | 1079.4 | -62.8 [-75.8, -52.5] |
| depressive disorders + COPD | 102.5 | -63.1 [-71.4, -53.5] |
| gynecological diseases + low back and neck pain + breast cancer | 415.6 | -63.2 [-72.5, -54.9] |
| gastritis and duodenitis, peptic ulcer disease + sense organ diseases | 734.7 | -63.7 [-74.5, -57.3] |
| IHD + hemoglobinopathies and hemolytic anemias + hyperlipidemia | 215.7 | -63.8 [-71.6, -49.5] |
| IHD + anxiety disorders + low back and neck pain | 104.1 | -63.9 [-70.9, -51.5] |
| EMBI-disorders + reproductive organ cancers + hyperlipidemia | 210.6 | -63.9 [-67.7, -52.6] |
| gastritis and duodenitis, peptic ulcer disease + anxiety disorders + tobacco use | 117.7 | -64.1 [-68.6, -62.1] |
| osteoarthritis + breast cancer + skin and subcutaneous diseases | 177.9 | -64.2 [-67.5, -50.5] |
| low back and neck pain + headache disorders + hypertension | 265.1 | -64.3 [-68.5, -53.5] |
| breast cancer + hyperlipidemia + hypertension | 519.4 | -64.3 [-73.9, -60.4] |
| diabetes + gastritis and duodenitis, peptic ulcer disease + skin and subcutaneous diseases | 367.8 | -64.6 [-68.3, -59.8] |
| gynecological diseases + Attention-deficit / hyperactivity disorder + skin and subcutaneous diseases | 122.4 | -64.6 [-67.1, -62.3] |
| diabetes + gynecological diseases + hyperlipidemia | 631 | -64.8 [-70.4, -49.7] |
| gynecological diseases + osteoarthritis + low back and neck pain | 284.6 | -64.9 [-69.6, -62.1] |
| IHD + anxiety disorders + hyperlipidemia | 175.7 | -65.3 [-80.1, -58.7] |
| gynecological diseases + Attention-deficit / hyperactivity disorder | 338.5 | -65.3 [-72.1, -52.1] |
| EMBI-disorders + sense organ diseases | 1388.9 | -65.4 [-75.9, -60] |
| gynecological diseases + anxiety disorders + tobacco use | 137.6 | -65.6 [-79.4, -58.5] |
| gastritis and duodenitis, peptic ulcer disease + headache disorders + skin and subcutaneous diseases | 171.8 | -66.6 [-70.8, -60.1] |
| EMBI-disorders + Attention-deficit / hyperactivity disorder + anxiety disorders | 117.2 | -66.6 [-76.8, -63.4] |
| low back and neck pain + breast cancer + colon and rectum cancer | 111.3 | -67.4 [-73.8, -51.1] |
| IHD + gastritis and duodenitis, peptic ulcer disease + hyperlipidemia | 230.4 | -67.5 [-74.7, -63.5] |
| colon and rectum cancer + hyperlipidemia + skin and subcutaneous diseases | 313 | -67.5 [-70.4, -63.6] |
| cirrhosis + gastritis and duodenitis, peptic ulcer disease + obesity | 141.5 | -67.8 [-77.6, -55.4] |
| colon and rectum cancer + hyperlipidemia + hypertension | 506.8 | -68.7 [-72.5, -57.6] |
| diabetes + sense organ diseases + skin and subcutaneous diseases | 497.8 | -70.1 [-82.1, -62.4] |
| gynecological diseases + hypertension + tobacco use | 132 | -70.2 [-79.6, -67.3] |
| gastritis and duodenitis, peptic ulcer disease + gynecological diseases + osteoarthritis | 147.8 | -70.4 [-85.9, -63.2] |
| low back and neck pain + colon and rectum cancer + hypertension | 219.5 | -70.4 [-74.1, -56] |
| hyperlipidemia + obesity + skin and subcutaneous diseases | 961 | -70.7 [-86.4, -59.2] |
| breast cancer + hypertension + sense organ diseases | 225.3 | -70.9 [-83.7, -66.1] |
| diabetes + obesity + sense organ diseases | 398.8 | -71.1 [-82.6, -54.4] |
| diabetes + gout | 187.4 | -71.2 [-87.1, -64.6] |
| diabetes + colon and rectum cancer + hypertension | 331 | -71.6 [-87.1, -61.6] |
| anxiety disorders + colon and rectum cancer + skin and subcutaneous diseases | 129.9 | -72.5 [-85.1, -62] |
| EMBI-disorders + depressive disorders + osteoarthritis | 131.1 | -73.2 [-80.7, -54.9] |
| IHD + EMBI-disorders + sense organ diseases | 121.9 | -73.3 [-83.7, -69.3] |
| anxiety disorders + asthma + skin and subcutaneous diseases | 216.5 | -73.6 [-75.8, -59.3] |
| reproductive organ cancers + hypertension + skin and subcutaneous diseases | 306.7 | -73.9 [-78.8, -69.5] |
| hemoglobinopathies and hemolytic anemias + sense organ diseases | 616.5 | -74 [-78.1, -59.6] |
| low back and neck pain + hyperlipidemia + skin and subcutaneous diseases | 1055.8 | -74 [-87.5, -68.7] |
| gastritis and duodenitis, peptic ulcer disease + hyperlipidemia + sense organ diseases | 301.1 | -74.2 [-84.9, -68.9] |
| low back and neck pain + hyperlipidemia + tobacco use | 184.6 | -74.3 [-86.2, -64.9] |
| gynecological diseases + breast cancer + reproductive organ cancers | 176.9 | -74.4 [-91, -67.7] |
| COPD + hypertension + tobacco use | 126.6 | -74.7 [-90.9, -66.8] |
| gastritis and duodenitis, peptic ulcer disease + gynecological diseases + asthma | 152.4 | -75.5 [-79.7, -65.1] |
| chronic kidney disease + sense organ diseases + skin and subcutaneous diseases | 105.2 | -76.1 [-86.2, -62] |
| anxiety disorders + hyperlipidemia + tobacco use | 150.1 | -76.3 [-81.2, -60.4] |
| diabetes + gynecological diseases + hypertension | 693.8 | -76.7 [-84.2, -61.5] |
| EMBI-disorders + low back and neck pain + colon and rectum cancer | 156.6 | -77.1 [-80.1, -60] |
| EMBI-disorders + headache disorders + hyperlipidemia | 158.1 | -77.5 [-96.2, -73.6] |
| EMBI-disorders + colon and rectum cancer + hyperlipidemia | 256.2 | -77.8 [-84.4, -68.6] |
| diabetes + gastritis and duodenitis, peptic ulcer disease + low back and neck pain | 289.1 | -78.8 [-88.1, -70.1] |
| low back and neck pain + COPD + hypertension | 111.6 | -78.8 [-90.8, -67.8] |
| low back and neck pain + hypertension + skin and subcutaneous diseases | 1266.6 | -79.2 [-83.3, -62.3] |
| diabetes + gynecological diseases + low back and neck pain | 347 | -79.4 [-97.4, -72.4] |
| gastritis and duodenitis, peptic ulcer disease + breast cancer + sense organ diseases | 101.2 | -79.4 [-95.3, -62.5] |
| diabetes + low back and neck pain + skin and subcutaneous diseases | 636 | -79.6 [-88.5, -68.6] |
| colon and rectum cancer + sense organ diseases | 433.4 | -79.8 [-89.3, -76.1] |
| diabetes + cirrhosis + gastritis and duodenitis, peptic ulcer disease | 119.9 | -80 [-82.9, -63.5] |
| anxiety disorders + low back and neck pain + skin and subcutaneous diseases | 933.6 | -80.1 [-89.7, -77.2] |
| EMBI-disorders + obesity | 2261.6 | -80.2 [-94.6, -62.4] |
| diabetes + osteoarthritis + sense organ diseases | 154.1 | -80.2 [-88.5, -62.5] |
| gynecological diseases + anxiety disorders + headache disorders | 262.4 | -81.6 [-89.4, -68.5] |
| Attention-deficit / hyperactivity disorder + anxiety disorders | 787 | -82.1 [-96.9, -74.5] |
| diabetes + low back and neck pain + breast cancer | 142 | -82.3 [-88.2, -75.5] |
| diabetes + asthma + hypertension | 245.8 | -82.3 [-87.5, -76.7] |
| depressive disorders + hyperlipidemia + skin and subcutaneous diseases | 388.6 | -82.9 [-100.9, -78.2] |
| diabetes + hemoglobinopathies and hemolytic anemias + low back and neck pain | 242.4 | -83.6 [-97.7, -71.8] |
| asthma + sense organ diseases + skin and subcutaneous diseases | 144.9 | -83.7 [-102.8, -72.3] |
| breast cancer + colon and rectum cancer + hyperlipidemia | 136.9 | -84.1 [-103.3, -69.2] |
| diabetes + asthma + hyperlipidemia | 207 | -84.3 [-95.5, -69.2] |
| gastritis and duodenitis, peptic ulcer disease + obesity + sense organ diseases | 179.9 | -85.4 [-90.2, -76.6] |
| cirrhosis + sense organ diseases | 194.6 | -86 [-93.2, -66.1] |
| gynecological diseases + sense organ diseases | 1233 | -87.1 [-108.5, -72.6] |
| gynecological diseases + anxiety disorders + skin and subcutaneous diseases | 942.9 | -88.4 [-98.2, -68.5] |
| IHD + diabetes + sense organ diseases | 158.8 | -88.7 [-108.1, -73.4] |
| diabetes + colon and rectum cancer + skin and subcutaneous diseases | 171.6 | -88.8 [-101.4, -83.7] |
| headache disorders + hypertension + skin and subcutaneous diseases | 233.8 | -89.4 [-97.5, -80.8] |
| EMBI-disorders + hemoglobinopathies and hemolytic anemias + obesity | 382.8 | -89.6 [-106.5, -68.4] |
| anxiety disorders + osteoarthritis + skin and subcutaneous diseases | 239.1 | -89.8 [-112.1, -78.3] |
| gastritis and duodenitis, peptic ulcer disease + breast cancer + hypertension | 203.8 | -89.9 [-105, -79.3] |
| gynecological diseases + low back and neck pain + skin and subcutaneous diseases | 1254.5 | -90.6 [-96.6, -77.7] |
| gastritis and duodenitis, peptic ulcer disease + anxiety disorders + sense organ diseases | 153.3 | -91 [-97.9, -69] |
| anxiety disorders + hyperlipidemia + hypertension | 891 | -91.1 [-99.9, -82.2] |
| IHD + low back and neck pain | 502 | -91.7 [-98.9, -75] |
| EMBI-disorders + reproductive organ cancers + skin and subcutaneous diseases | 225.9 | -92.1 [-114.4, -83.3] |
| hemoglobinopathies and hemolytic anemias + anxiety disorders + low back and neck pain | 294.7 | -93.7 [-101.3, -87.1] |
| gastritis and duodenitis, peptic ulcer disease + gynecological diseases + hypertension | 458.4 | -93.7 [-99.9, -89.8] |
| anxiety disorders + obesity + skin and subcutaneous diseases | 546.4 | -93.8 [-113.7, -88.9] |
| gynecological diseases + low back and neck pain + tobacco use | 123.8 | -95 [-112.8, -74.5] |
| hemoglobinopathies and hemolytic anemias + breast cancer | 448.4 | -96.7 [-110.5, -77.9] |
| EMBI-disorders + colon and rectum cancer + skin and subcutaneous diseases | 249.5 | -96.9 [-119.8, -74.3] |
| asthma + hypertension + skin and subcutaneous diseases | 302.5 | -97.7 [-120.5, -80.5] |
| gynecological diseases + colon and rectum cancer + skin and subcutaneous diseases | 253.3 | -97.8 [-103.6, -94.3] |
| depressive disorders + hyperlipidemia + tobacco use | 105.6 | -98.2 [-105.8, -76.7] |
| gastritis and duodenitis, peptic ulcer disease + breast cancer + skin and subcutaneous diseases | 238.1 | -98.4 [-112.9, -85] |
| diabetes + anxiety disorders + hyperlipidemia | 490.9 | -98.8 [-103.4, -83.5] |
| IHD + EMBI-disorders + hyperlipidemia | 396.7 | -98.8 [-118.8, -83.6] |
| EMBI-disorders + depressive disorders + obesity | 317.1 | -99.1 [-111.2, -78.2] |
| gynecological diseases + obesity + sense organ diseases | 231.1 | -99.4 [-107.8, -93.8] |
| gynecological diseases + osteoarthritis + obesity | 203.1 | -99.4 [-124, -87.2] |
| anxiety disorders + low back and neck pain + hyperlipidemia | 515.2 | -99.8 [-121.3, -89.8] |
| gynecological diseases + low back and neck pain + colon and rectum cancer | 156 | -100.5 [-118.5, -76.9] |
| gastritis and duodenitis, peptic ulcer disease + hypertension + skin and subcutaneous diseases | 771.6 | -100.7 [-121.7, -77.1] |
| EMBI-disorders + gynecological diseases + obesity | 607.9 | -100.9 [-108.1, -90.6] |
| substance use disorders + osteoarthritis + low back and neck pain | 113.5 | -101.2 [-118.5, -91.8] |
| diabetes + gynecological diseases + breast cancer | 208.2 | -102.7 [-112.9, -77.3] |
| cirrhosis + EMBI-disorders + obesity | 176.2 | -103.2 [-121.8, -93.1] |
| gynecological diseases + headache disorders + skin and subcutaneous diseases | 345.8 | -103.2 [-115.2, -94.4] |
| diabetes + hemoglobinopathies and hemolytic anemias + obesity | 327.7 | -103.4 [-113.6, -85.5] |
| EMBI-disorders + osteoarthritis + breast cancer | 115.6 | -103.6 [-116.6, -98.8] |
| hemoglobinopathies and hemolytic anemias + colon and rectum cancer + skin and subcutaneous diseases | 105.6 | -104.1 [-112.5, -82.3] |
| gynecological diseases + hyperlipidemia + skin and subcutaneous diseases | 782.9 | -104.3 [-120.3, -86.9] |
| EMBI-disorders + osteoarthritis + low back and neck pain | 353.7 | -104.4 [-108.5, -93.7] |
| colon and rectum cancer + sense organ diseases + skin and subcutaneous diseases | 177 | -104.6 [-123, -98.4] |
| EMBI-disorders + hyperlipidemia + obesity | 904 | -104.8 [-119.4, -97.1] |
| EMBI-disorders + headache disorders + obesity | 139.7 | -104.8 [-109.8, -90.6] |
| diabetes + gynecological diseases + osteoarthritis | 134.8 | -105.4 [-118.8, -90.3] |
| diabetes + breast cancer + obesity | 191.2 | -105.6 [-121.3, -94.3] |
| hemoglobinopathies and hemolytic anemias + depressive disorders + obesity | 163.3 | -105.7 [-113.1, -91.1] |
| gynecological diseases + low back and neck pain + hypertension | 692.1 | -106.3 [-119.3, -91.8] |
| cirrhosis + hemoglobinopathies and hemolytic anemias + hyperlipidemia | 104.7 | -106.9 [-128.3, -103] |
| gastritis and duodenitis, peptic ulcer disease + gynecological diseases + hemoglobinopathies and hemolytic anemias | 275.8 | -107.2 [-120.8, -92] |
| hemoglobinopathies and hemolytic anemias + depressive disorders + low back and neck pain | 187.6 | -107.3 [-116.5, -93.8] |
| anxiety disorders + low back and neck pain + hypertension | 670.9 | -107.6 [-114.3, -93.2] |
| diabetes + EMBI-disorders + obesity | 722 | -107.7 [-115, -92.4] |
| hemoglobinopathies and hemolytic anemias + osteoarthritis + hyperlipidemia | 163.2 | -109.4 [-130.4, -102] |
| substance use disorders + hyperlipidemia + skin and subcutaneous diseases | 117.3 | -109.6 [-135.1, -90.8] |
| gynecological diseases + hemoglobinopathies and hemolytic anemias + skin and subcutaneous diseases | 538.5 | -109.6 [-115.8, -95.3] |
| diabetes + gastritis and duodenitis, peptic ulcer disease + sense organ diseases | 192.8 | -110 [-119.8, -86.1] |
| EMBI-disorders + anxiety disorders + obesity | 436.3 | -110.4 [-127.1, -100] |
| gastritis and duodenitis, peptic ulcer disease + low back and neck pain + skin and subcutaneous diseases | 671.3 | -110.7 [-121.6, -90.5] |
| breast cancer + reproductive organ cancers + skin and subcutaneous diseases | 160.3 | -110.8 [-115.4, -96.6] |
| colon and rectum cancer + hypertension + skin and subcutaneous diseases | 340.8 | -111.3 [-115.3, -83.5] |
| IHD + hypertension + sense organ diseases | 239.7 | -111.3 [-120.2, -98.1] |
| diabetes + breast cancer + hypertension | 348.1 | -111.7 [-127.4, -84.2] |
| diabetes + hemoglobinopathies and hemolytic anemias + osteoarthritis | 113.9 | -111.9 [-117, -90.1] |
| EMBI-disorders + Attention-deficit / hyperactivity disorder | 259.2 | -112.5 [-123, -85] |
| anxiety disorders + substance use disorders + hyperlipidemia | 124.9 | -112.6 [-116.4, -98.4] |
| diabetes + low back and neck pain + hypertension | 1004.7 | -113.4 [-136, -101.3] |
| diabetes + hemoglobinopathies and hemolytic anemias + sense organ diseases | 185.7 | -114 [-122.5, -105.8] |
| anxiety disorders + headache disorders + skin and subcutaneous diseases | 282.9 | -114.4 [-121.7, -109.7] |
| cirrhosis + gynecological diseases + hyperlipidemia | 106.2 | -114.8 [-121.1, -86.4] |
| gastritis and duodenitis, peptic ulcer disease + anxiety disorders + hyperlipidemia | 345.8 | -114.9 [-141.1, -101.6] |
| diabetes + EMBI-disorders + asthma | 143 | -115 [-122, -103] |
| hemoglobinopathies and hemolytic anemias + Attention-deficit / hyperactivity disorder | 110.6 | -115.5 [-143.4, -94] |
| EMBI-disorders + breast cancer + hyperlipidemia | 354.4 | -115.7 [-131.2, -109.4] |
| EMBI-disorders + low back and neck pain + sense organ diseases | 392.2 | -115.7 [-123.8, -90.5] |
| EMBI-disorders + depressive disorders + low back and neck pain | 376.7 | -115.8 [-136.9, -90.6] |
| gynecological diseases + hyperlipidemia + hypertension | 946.9 | -117 [-135.1, -105.3] |
| gastritis and duodenitis, peptic ulcer disease + gynecological diseases + hyperlipidemia | 367.8 | -117.6 [-128.7, -89.4] |
| EMBI-disorders + low back and neck pain + asthma | 194.7 | -118 [-122.3, -97.4] |
| diabetes + EMBI-disorders + sense organ diseases | 423.6 | -118.3 [-139, -100.2] |
| obesity + sense organ diseases + skin and subcutaneous diseases | 399.3 | -118.4 [-132.9, -95.6] |
| cirrhosis + low back and neck pain + skin and subcutaneous diseases | 159.3 | -118.7 [-146.5, -96.5] |
| breast cancer + colon and rectum cancer + hypertension | 158.4 | -119 [-142.2, -101.9] |
| diabetes + hypertension | 5043.9 | -119.2 [-148.2, -110.6] |
| gynecological diseases + osteoarthritis + hypertension | 272 | -120.1 [-144.1, -108.9] |
| hyperlipidemia + tobacco use + skin and subcutaneous diseases | 209.6 | -120.2 [-146.5, -113.4] |
| EMBI-disorders + depressive disorders + hyperlipidemia | 370.7 | -120.4 [-132.4, -91] |
| chronic kidney disease + gastritis and duodenitis, peptic ulcer disease + hyperlipidemia | 115.5 | -120.7 [-140.1, -105.1] |
| colon and rectum cancer + hypertension + sense organ diseases | 171.3 | -121.3 [-139.4, -95] |
| gastritis and duodenitis, peptic ulcer disease + anxiety disorders + hypertension | 445 | -121.3 [-128.9, -100.1] |
| osteoarthritis + sense organ diseases + skin and subcutaneous diseases | 256.5 | -121.8 [-147.8, -97] |
| EMBI-disorders + osteoarthritis + hypertension | 440.8 | -122.4 [-133, -96.1] |
| hypertension + obesity + sense organ diseases | 557.7 | -122.7 [-129.1, -92.5] |
| chronic kidney disease + diabetes + sense organ diseases | 154 | -123.4 [-147.9, -115.5] |
| diabetes + osteoarthritis + skin and subcutaneous diseases | 287 | -124.3 [-132.6, -116.2] |
| IHD + osteoarthritis + hyperlipidemia | 150.2 | -124.5 [-131.5, -104.8] |
| EMBI-disorders + breast cancer + hypertension | 364.1 | -124.8 [-144.9, -94.7] |
| EMBI-disorders + substance use disorders + hyperlipidemia | 126 | -125.1 [-134.5, -113.8] |
| breast cancer + sense organ diseases | 707 | -125.6 [-138.5, -121.5] |
| diabetes + breast cancer | 624.3 | -126.5 [-151.9, -111.3] |
| IHD + hyperlipidemia + sense organ diseases | 222 | -127.5 [-138.4, -104.6] |
| IHD + gastritis and duodenitis, peptic ulcer disease + obesity | 127.4 | -128.6 [-153.5, -97.4] |
| gastritis and duodenitis, peptic ulcer disease + EMBI-disorders + asthma | 155.7 | -128.6 [-139.7, -108.1] |
| anxiety disorders + hyperlipidemia | 1714 | -128.9 [-147.3, -108.2] |
| EMBI-disorders + headache disorders + hypertension | 183.3 | -129 [-135.3, -107.9] |
| gastritis and duodenitis, peptic ulcer disease + hypertension + sense organ diseases | 337.6 | -129.1 [-143.9, -98.8] |
| reproductive organ cancers + skin and subcutaneous diseases | 1097.7 | -129.3 [-136.4, -117.4] |
| gynecological diseases + anxiety disorders + low back and neck pain | 710.7 | -129.5 [-150.5, -112.6] |
| gynecological diseases + hemoglobinopathies and hemolytic anemias + sense organ diseases | 171.1 | -129.8 [-150.4, -121.8] |
| EMBI-disorders + asthma + hypertension | 243.8 | -131.2 [-148, -101.9] |
| IHD + low back and neck pain + skin and subcutaneous diseases | 206.3 | -131.6 [-160.6, -117.8] |
| low back and neck pain + breast cancer + skin and subcutaneous diseases | 472 | -133 [-140.1, -124] |
| hemoglobinopathies and hemolytic anemias + osteoarthritis + obesity | 146.8 | -133.7 [-139.5, -121] |
| IHD + gynecological diseases + hyperlipidemia | 160.3 | -134.6 [-143.5, -125.9] |
| gynecological diseases + sense organ diseases + skin and subcutaneous diseases | 560 | -135.2 [-164.6, -110.6] |
| EMBI-disorders + asthma + hyperlipidemia | 199 | -135.9 [-164.8, -121.2] |
| IHD + EMBI-disorders + obesity | 213.7 | -136.2 [-151.3, -120.8] |
| gastritis and duodenitis, peptic ulcer disease + EMBI-disorders + headache disorders | 125.1 | -136.6 [-143, -123.6] |
| gastritis and duodenitis, peptic ulcer disease + hemoglobinopathies and hemolytic anemias + hypertension | 407.8 | -138.1 [-162.5, -107.3] |
| substance use disorders + low back and neck pain + obesity | 138.6 | -138.4 [-172.8, -133.8] |
| cirrhosis + gynecological diseases + low back and neck pain | 103.6 | -139.7 [-152, -106.5] |
| EMBI-disorders + headache disorders | 584.6 | -140.6 [-150.6, -108] |
| EMBI-disorders + low back and neck pain + breast cancer | 285.3 | -142.3 [-163.6, -128.2] |
| hemoglobinopathies and hemolytic anemias + hypertension + sense organ diseases | 277.4 | -142.6 [-155.3, -131.2] |
| diabetes + cirrhosis + hypertension | 268.3 | -143 [-166.6, -115.2] |
| EMBI-disorders + substance use disorders + low back and neck pain | 178.2 | -143.4 [-167.3, -112.3] |
| EMBI-disorders + colon and rectum cancer + sense organ diseases | 109.7 | -143.6 [-165.8, -108.8] |
| gastritis and duodenitis, peptic ulcer disease + EMBI-disorders + breast cancer | 168.8 | -144 [-161.7, -127.5] |
| gynecological diseases + breast cancer + sense organ diseases | 245 | -144.6 [-179.2, -130] |
| gynecological diseases + breast cancer + colon and rectum cancer | 178 | -145.8 [-164.8, -134.2] |
| gastritis and duodenitis, peptic ulcer disease + hemoglobinopathies and hemolytic anemias + osteoarthritis | 119.6 | -146.2 [-176.5, -135.2] |
| EMBI-disorders + hemoglobinopathies and hemolytic anemias + breast cancer | 154.9 | -146.4 [-180.2, -119.3] |
| EMBI-disorders + obesity + sense organ diseases | 304.8 | -146.8 [-171.1, -126.6] |
| chronic kidney disease + diabetes + low back and neck pain | 134.3 | -146.9 [-180.3, -118.2] |
| hemoglobinopathies and hemolytic anemias + low back and neck pain + sense organ diseases | 186.5 | -147.9 [-181.6, -113.8] |
| diabetes + EMBI-disorders + low back and neck pain | 552 | -148 [-167, -118.8] |
| IHD + osteoarthritis + hypertension | 175.2 | -148.8 [-168.4, -112.1] |
| hemoglobinopathies and hemolytic anemias + sense organ diseases + skin and subcutaneous diseases | 275.1 | -149.6 [-183.6, -129.6] |
| diabetes + EMBI-disorders + osteoarthritis | 241.3 | -150 [-184.8, -114.5] |
| breast cancer + hypertension + skin and subcutaneous diseases | 470.2 | -150.8 [-157, -138] |
| hemoglobinopathies and hemolytic anemias + depressive disorders + hyperlipidemia | 158 | -152.4 [-165.1, -129.8] |
| IHD + sense organ diseases + skin and subcutaneous diseases | 143.4 | -152.6 [-180, -123.4] |
| gastritis and duodenitis, peptic ulcer disease + gynecological diseases + sense organ diseases | 198.8 | -154.4 [-167.4, -117.2] |
| EMBI-disorders + hypertension + sense organ diseases | 591.8 | -154.8 [-189.6, -123.8] |
| EMBI-disorders + anxiety disorders + low back and neck pain | 589.6 | -154.8 [-187, -130.8] |
| chronic kidney disease + hypertension + sense organ diseases | 176.9 | -154.8 [-167.1, -138.6] |
| hemoglobinopathies and hemolytic anemias + breast cancer + hypertension | 165.2 | -155 [-174.9, -144.8] |
| EMBI-disorders + anxiety disorders + headache disorders | 184.3 | -155.5 [-188.7, -137.6] |
| cirrhosis + gastritis and duodenitis, peptic ulcer disease + hypertension | 200.9 | -155.8 [-178.5, -146.9] |
| EMBI-disorders + anxiety disorders + osteoarthritis | 172.9 | -156.4 [-175.4, -119.1] |
| hyperlipidemia + skin and subcutaneous diseases | 3892.8 | -156.6 [-193.7, -126] |
| IHD + depressive disorders + hyperlipidemia | 119.8 | -159.5 [-197.2, -120.5] |
| gynecological diseases + osteoarthritis + skin and subcutaneous diseases | 319.9 | -160 [-191.3, -130.2] |
| inguinal, femoral, and abdominal hernia + gastritis and duodenitis, peptic ulcer disease + low back and neck pain | 126.5 | -161.8 [-193.6, -156.4] |
| hemoglobinopathies and hemolytic anemias + obesity + skin and subcutaneous diseases | 406.2 | -162 [-190, -156.8] |
| breast cancer + colon and rectum cancer + skin and subcutaneous diseases | 201.7 | -163.1 [-176.5, -123.6] |
| IHD + gastritis and duodenitis, peptic ulcer disease + low back and neck pain | 118.4 | -163.4 [-185.7, -136] |
| gastritis and duodenitis, peptic ulcer disease + hemoglobinopathies and hemolytic anemias + obesity | 266.1 | -163.9 [-169.9, -153] |
| gynecological diseases + hyperlipidemia | 1942.8 | -164.5 [-178.2, -140.7] |
| IHD + obesity + sense organ diseases | 102.4 | -164.8 [-174.3, -134.3] |
| hemoglobinopathies and hemolytic anemias + osteoarthritis + hypertension | 220 | -165.5 [-193.7, -153.9] |
| diabetes + asthma + skin and subcutaneous diseases | 153 | -166 [-192, -146.2] |
| EMBI-disorders + breast cancer + colon and rectum cancer | 126.5 | -166.1 [-197.3, -146.3] |
| hemoglobinopathies and hemolytic anemias + hypertension + tobacco use | 151.8 | -166.4 [-206.9, -157.4] |
| hemoglobinopathies and hemolytic anemias + low back and neck pain + breast cancer | 125.4 | -168.8 [-205.5, -145.8] |
| cirrhosis + hypertension + sense organ diseases | 100.1 | -168.8 [-209.4, -160.7] |
| diabetes + EMBI-disorders + breast cancer | 207.9 | -168.9 [-181.5, -134.6] |
| EMBI-disorders + hyperlipidemia + sense organ diseases | 562 | -169.1 [-191.6, -154.1] |
| hemoglobinopathies and hemolytic anemias + osteoarthritis + low back and neck pain | 172.4 | -171.9 [-199.3, -161.2] |
| EMBI-disorders + osteoarthritis + sense organ diseases | 168.2 | -172.4 [-188.2, -149.8] |
| hypertension + sense organ diseases + skin and subcutaneous diseases | 785.1 | -173.7 [-196.5, -147.1] |
| EMBI-disorders + breast cancer | 1120.3 | -174.9 [-192.8, -167.7] |
| gastritis and duodenitis, peptic ulcer disease + EMBI-disorders + sense organ diseases | 237.2 | -175.6 [-196.4, -144.7] |
| cirrhosis + obesity + skin and subcutaneous diseases | 162.3 | -175.8 [-195, -135.1] |
| EMBI-disorders + gynecological diseases + osteoarthritis | 222.7 | -176 [-208.2, -170.5] |
| gastritis and duodenitis, peptic ulcer disease + gynecological diseases + low back and neck pain | 453.9 | -176 [-202.7, -165] |
| breast cancer + sense organ diseases + skin and subcutaneous diseases | 314.1 | -176.2 [-192.2, -147.7] |
| hemoglobinopathies and hemolytic anemias + anxiety disorders + hyperlipidemia | 231.3 | -176.3 [-212.4, -147.9] |
| gastritis and duodenitis, peptic ulcer disease + EMBI-disorders + gynecological diseases | 427.4 | -177.6 [-194.2, -171.6] |
| gastritis and duodenitis, peptic ulcer disease + asthma + skin and subcutaneous diseases | 206.4 | -177.6 [-185.5, -145.7] |
| EMBI-disorders + low back and neck pain + obesity | 607.9 | -177.7 [-219.5, -162.1] |
| IHD + osteoarthritis | 236.4 | -177.9 [-191.4, -139.5] |
| gynecological diseases + hemoglobinopathies and hemolytic anemias + asthma | 169.4 | -178.4 [-216.3, -155] |
| breast cancer + reproductive organ cancers | 456 | -179.9 [-201.7, -149.5] |
| IHD + diabetes + low back and neck pain | 178.4 | -180.4 [-194.7, -174.5] |
| EMBI-disorders + gynecological diseases + headache disorders | 222.1 | -182.3 [-196.9, -168.4] |
| EMBI-disorders + breast cancer + sense organ diseases | 194.3 | -182.7 [-210.4, -149.5] |
| EMBI-disorders + gynecological diseases + skin and subcutaneous diseases | 1144.7 | -184.4 [-206.9, -145.4] |
| cirrhosis + hypertension + obesity | 239.4 | -184.6 [-221.6, -156.4] |
| EMBI-disorders + low back and neck pain + headache disorders | 270.1 | -185.9 [-225, -148.6] |
| EMBI-disorders + asthma + skin and subcutaneous diseases | 259.4 | -186.9 [-199.9, -171.8] |
| EMBI-disorders + gynecological diseases + hyperlipidemia | 695.9 | -189.4 [-235.6, -168.1] |
| hyperlipidemia + hypertension + skin and subcutaneous diseases | 2064.6 | -189.8 [-199.5, -183.6] |
| EMBI-disorders + headache disorders + skin and subcutaneous diseases | 251.4 | -191.9 [-223.5, -183.4] |
| gastritis and duodenitis, peptic ulcer disease + gynecological diseases + skin and subcutaneous diseases | 584.8 | -195.8 [-227.6, -149.8] |
| EMBI-disorders + gynecological diseases + sense organ diseases | 361.1 | -195.9 [-236.5, -157.4] |
| hemoglobinopathies and hemolytic anemias + hyperlipidemia + sense organ diseases | 234.8 | -196.4 [-210.5, -186.7] |
| hemoglobinopathies and hemolytic anemias + obesity + sense organ diseases | 149.7 | -198.4 [-238.4, -189.8] |
| diabetes + breast cancer + skin and subcutaneous diseases | 231.7 | -198.6 [-212.8, -156.1] |
| hemoglobinopathies and hemolytic anemias + anxiety disorders + headache disorders | 102.7 | -199.1 [-232.8, -185.7] |
| gynecological diseases + hemoglobinopathies and hemolytic anemias + osteoarthritis | 111.9 | -200.2 [-249.4, -168.2] |
| chronic kidney disease + osteoarthritis + hypertension | 105 | -200.7 [-224.4, -152.7] |
| hemoglobinopathies and hemolytic anemias + osteoarthritis | 412.5 | -200.9 [-218.5, -179.6] |
| IHD + osteoarthritis + skin and subcutaneous diseases | 101.1 | -202.2 [-241.8, -158.1] |
| chronic kidney disease + hypertension + obesity | 297.2 | -202.8 [-253, -162] |
| IHD + obesity + skin and subcutaneous diseases | 213.8 | -203.2 [-253, -174.4] |
| EMBI-disorders + hypertension + obesity | 1128.9 | -205.7 [-220, -193.1] |
| hemoglobinopathies and hemolytic anemias + headache disorders + hypertension | 103.8 | -206.4 [-237.9, -163.6] |
| chronic kidney disease + osteoarthritis | 144.6 | -207.3 [-248.8, -159.6] |
| gastritis and duodenitis, peptic ulcer disease + sense organ diseases + skin and subcutaneous diseases | 336.2 | -207.6 [-258.2, -194.3] |
| diabetes + hemoglobinopathies and hemolytic anemias + asthma | 108 | -209.4 [-234.2, -190.6] |
| gynecological diseases + hemoglobinopathies and hemolytic anemias + hyperlipidemia | 296.2 | -209.4 [-222.9, -181.4] |
| hemoglobinopathies and hemolytic anemias + hyperlipidemia + tobacco use | 104.1 | -209.8 [-262.3, -166.5] |
| gastritis and duodenitis, peptic ulcer disease + hyperlipidemia + skin and subcutaneous diseases | 654.8 | -211.2 [-240.6, -167.4] |
| diabetes + substance use disorders + low back and neck pain | 110.1 | -211.8 [-218.9, -184.3] |
| hemoglobinopathies and hemolytic anemias + low back and neck pain + skin and subcutaneous diseases | 516.2 | -213.1 [-260.9, -168.9] |
| EMBI-disorders + low back and neck pain + skin and subcutaneous diseases | 1104.2 | -213.8 [-234, -162.3] |
| hemoglobinopathies and hemolytic anemias + asthma + obesity | 163.3 | -214.8 [-238.6, -181] |
| IHD + low back and neck pain + hypertension | 343 | -215.6 [-224.1, -197.6] |
| hemoglobinopathies and hemolytic anemias + breast cancer + hyperlipidemia | 131.9 | -217.3 [-235.4, -203.7] |
| substance use disorders + low back and neck pain + hypertension | 260.4 | -218.2 [-225.2, -169.7] |
| stroke + hyperlipidemia + skin and subcutaneous diseases | 145.3 | -226.4 [-233.2, -178.6] |
| chronic kidney disease + hyperlipidemia + sense organ diseases | 144.7 | -226.6 [-270.8, -190] |
| hemoglobinopathies and hemolytic anemias + anxiety disorders + asthma | 127 | -228.7 [-257.6, -182] |
| IHD + low back and neck pain + obesity | 158.6 | -230 [-239.6, -206.6] |
| diabetes + hyperlipidemia | 4625.1 | -230.6 [-266.5, -218.7] |
| hemoglobinopathies and hemolytic anemias + headache disorders + skin and subcutaneous diseases | 130.9 | -231 [-265.9, -207.4] |
| EMBI-disorders + obesity + skin and subcutaneous diseases | 814.5 | -232.8 [-287.7, -205.2] |
| gastritis and duodenitis, peptic ulcer disease + hemoglobinopathies and hemolytic anemias + sense organ diseases | 148.9 | -236.8 [-251.5, -216.3] |
| cirrhosis + osteoarthritis | 126.7 | -237.7 [-259.5, -222.7] |
| gynecological diseases + hemoglobinopathies and hemolytic anemias + headache disorders | 122.6 | -239.4 [-273.9, -198.3] |
| anxiety disorders + hyperlipidemia + skin and subcutaneous diseases | 611.6 | -243.8 [-267.1, -203.8] |
| EMBI-disorders + hyperlipidemia + hypertension | 1887.8 | -244.4 [-303, -187.1] |
| substance use disorders + low back and neck pain + hyperlipidemia | 170.6 | -245.5 [-294, -195.1] |
| IHD + EMBI-disorders + low back and neck pain | 177.1 | -249.1 [-261.9, -221.8] |
| hemoglobinopathies and hemolytic anemias + low back and neck pain | 1219.5 | -249.2 [-275.7, -210.8] |
| EMBI-disorders + sense organ diseases + skin and subcutaneous diseases | 601.1 | -249.6 [-262.2, -188.5] |
| chronic kidney disease + hyperlipidemia + skin and subcutaneous diseases | 229.1 | -250.4 [-294.2, -242.8] |
| EMBI-disorders + hemoglobinopathies and hemolytic anemias + headache disorders | 106.6 | -251 [-276, -228.5] |
| hemoglobinopathies and hemolytic anemias + asthma + hyperlipidemia | 145.8 | -251.1 [-281.1, -236.8] |
| hemoglobinopathies and hemolytic anemias + hyperlipidemia + obesity | 381.9 | -253.7 [-280.1, -202.8] |
| diabetes + cirrhosis + low back and neck pain | 107.7 | -253.7 [-314.2, -203.4] |
| hemoglobinopathies and hemolytic anemias + low back and neck pain + asthma | 163.8 | -256.3 [-303.2, -243.7] |
| diabetes + cirrhosis + obesity | 165.7 | -257.6 [-277.4, -247.9] |
| EMBI-disorders + low back and neck pain + hyperlipidemia | 863.5 | -257.7 [-288.4, -228.1] |
| hemoglobinopathies and hemolytic anemias + hypertension + obesity | 565.5 | -264.3 [-293.9, -221.4] |
| hemoglobinopathies and hemolytic anemias + low back and neck pain + headache disorders | 139.9 | -270.2 [-289.5, -250.7] |
| gynecological diseases + hemoglobinopathies and hemolytic anemias + low back and neck pain | 382.7 | -272.6 [-281.7, -262.9] |
| breast cancer + colon and rectum cancer | 529.8 | -275.9 [-325, -245] |
| chronic kidney disease + diabetes + obesity | 228.1 | -277.1 [-327.2, -217.5] |
| hemoglobinopathies and hemolytic anemias + headache disorders | 306.2 | -280.3 [-306.7, -247.1] |
| hemoglobinopathies and hemolytic anemias + breast cancer + skin and subcutaneous diseases | 193.8 | -281.6 [-320.6, -270] |
| cirrhosis + hyperlipidemia + obesity | 179.6 | -283.9 [-348.6, -246.5] |
| EMBI-disorders + low back and neck pain + hypertension | 960.5 | -286.1 [-331.8, -240.2] |
| EMBI-disorders + hemoglobinopathies and hemolytic anemias + osteoarthritis | 159.9 | -286.7 [-358.2, -243.8] |
| chronic kidney disease + diabetes + hyperlipidemia | 398.6 | -295.9 [-316.8, -260.9] |
| EMBI-disorders + anxiety disorders + hyperlipidemia | 539.6 | -298.9 [-368.9, -274.8] |
| gastritis and duodenitis, peptic ulcer disease + EMBI-disorders + hyperlipidemia | 560.1 | -298.9 [-358.8, -265.3] |
| EMBI-disorders + gynecological diseases + low back and neck pain | 787.6 | -301.2 [-327.9, -288.3] |
| chronic kidney disease + hyperlipidemia | 676.8 | -304.8 [-369, -294.1] |
| diabetes + EMBI-disorders + hyperlipidemia | 1314.6 | -306.5 [-344.3, -252.4] |
| chronic kidney disease + low back and neck pain + hypertension | 198.1 | -306.7 [-361.6, -256.8] |
| gastritis and duodenitis, peptic ulcer disease + hemoglobinopathies and hemolytic anemias + asthma | 150.8 | -309.1 [-383.8, -289.7] |
| IHD + hyperlipidemia + skin and subcutaneous diseases | 419.5 | -310.7 [-356.4, -290.3] |
| EMBI-disorders + hemoglobinopathies and hemolytic anemias + asthma | 175.3 | -312.6 [-330.8, -258.3] |
| EMBI-disorders + breast cancer + skin and subcutaneous diseases | 474.5 | -316.1 [-382.4, -238] |
| diabetes + hemoglobinopathies and hemolytic anemias + hyperlipidemia | 511.6 | -316.6 [-391, -276.2] |
| hemoglobinopathies and hemolytic anemias + low back and neck pain + obesity | 307.2 | -319 [-341, -290.7] |
| gastritis and duodenitis, peptic ulcer disease + hemoglobinopathies and hemolytic anemias + hyperlipidemia | 311.3 | -322.7 [-367.2, -264.2] |
| cirrhosis + low back and neck pain + hypertension | 169.1 | -322.8 [-359.8, -290.6] |
| hemoglobinopathies and hemolytic anemias + low back and neck pain + hypertension | 463.3 | -326.7 [-405.8, -302.4] |
| hemoglobinopathies and hemolytic anemias + asthma + hypertension | 206.7 | -333.3 [-359.2, -305.1] |
| cirrhosis + low back and neck pain + obesity | 118 | -346.1 [-366.5, -270.2] |
| cirrhosis + gastritis and duodenitis, peptic ulcer disease + low back and neck pain | 119.2 | -348.6 [-400.6, -333.4] |
| hemoglobinopathies and hemolytic anemias + osteoarthritis + skin and subcutaneous diseases | 186.6 | -358.4 [-443.3, -329] |
| gastritis and duodenitis, peptic ulcer disease + EMBI-disorders + low back and neck pain | 470 | -360 [-431.7, -339.9] |
| hemoglobinopathies and hemolytic anemias + hyperlipidemia + hypertension | 770 | -361.7 [-382.2, -284.3] |
| EMBI-disorders + osteoarthritis + skin and subcutaneous diseases | 393.9 | -365.7 [-411.8, -320.2] |
| chronic kidney disease + hyperlipidemia + hypertension | 505.9 | -371.5 [-388.8, -302] |
| cirrhosis + low back and neck pain + hyperlipidemia | 134.9 | -376.9 [-447.8, -327.6] |
| diabetes + cirrhosis + hyperlipidemia | 238 | -393 [-469.5, -315.2] |
| cirrhosis + gastritis and duodenitis, peptic ulcer disease + hyperlipidemia | 153.8 | -394.3 [-462.2, -375.1] |
| hemoglobinopathies and hemolytic anemias + low back and neck pain + hyperlipidemia | 356.6 | -403.7 [-451.1, -323.8] |
| cirrhosis + EMBI-disorders + hyperlipidemia | 212.8 | -407.9 [-460.8, -336.9] |
| IHD + low back and neck pain + hyperlipidemia | 305 | -412.8 [-487.2, -388.2] |
| chronic kidney disease + hyperlipidemia + obesity | 223.8 | -415.6 [-511.9, -344.9] |
| gastritis and duodenitis, peptic ulcer disease + hemoglobinopathies and hemolytic anemias + low back and neck pain | 291.8 | -437.6 [-451.3, -372.5] |
| hemoglobinopathies and hemolytic anemias + asthma + skin and subcutaneous diseases | 218.1 | -460.9 [-530, -394.7] |
| cirrhosis + hyperlipidemia + hypertension | 316.1 | -474.1 [-573.2, -439.4] |
| hemoglobinopathies and hemolytic anemias + asthma | 570 | -476 [-530.1, -417.1] |
| EMBI-disorders + hemoglobinopathies and hemolytic anemias + hyperlipidemia | 514.3 | -495 [-616.9, -448.1] |
| cirrhosis + hyperlipidemia + skin and subcutaneous diseases | 190.5 | -503.8 [-542.4, -390.5] |
| EMBI-disorders + hyperlipidemia + skin and subcutaneous diseases | 1210.9 | -534.9 [-637.7, -405.2] |
| chronic kidney disease + low back and neck pain + hyperlipidemia | 149.7 | -545.1 [-573.9, -499.1] |
| hemoglobinopathies and hemolytic anemias + hyperlipidemia + skin and subcutaneous diseases | 488.4 | -558.4 [-689.3, -501.5] |
| cirrhosis + hyperlipidemia | 514.2 | -610.2 [-701.5, -491.2] |
| hemoglobinopathies and hemolytic anemias + hyperlipidemia | 1281.9 | -702.4 [-762.7, -554.2] |
| EMBI-disorders + hyperlipidemia | 3523.9 | -732.7 [-906, -618] |

* IHD = ischemic heart disease, EMBI = endocrine, metabolic, blood, and immune disorders

1. <https://scikit-learn.org/0.17/modules/generated/sklearn.linear_model.SGDRegressor.html> [↑](#footnote-ref-1)
