# Supplementary material for: Estimating health spending associated with chronic multimorbidity in 2018: An observational study among adults in the United States
Source: PLoS Med. 2023 Apr 4;20(4):e1004205. doi: 10.1371/journal.pmed.1004205 (PMC10072449; doi:10.1371/journal.pmed.1004205)
Supplement: Attachment — Submitted filename: plosmed_RR_dec2022.docx [file pmed.1004205.s003.docx]

Response to editors’ and reviewers’ comments

--------------

GENERAL
Please respond to all editor and reviewer comments detailed below, in full.
Please note reviewer comments below requiring the use of language which clearly clarifies your descriptions and definitions/categorizations 
Please address the conceptualization issues identified

Thank you for reporting (we think!) according to RECORD – see below, please clarify.  
Please review the guidance using the link below regarding reporting of studies: 
<https://eur03.safelinks.protection.outlook.com/?url=http%3A%2F%2Fwww.equator-network.org%2F%3Fpost_type%3Deq_guidelines%26eq_guidelines_study_design%3Deconomic-evaluations%26eq_guidelines_clinical_specialty%3D0%26eq_guidelines_report_section%3D0%26s%3D&data=05%7C01%7Cachang%40health.sdu.dk%7Cc7974603d4fa4e8497e208dadd2b4f1e%7C9a97c27db83e4694b35354bdbf18ab5b%7C0%7C0%7C638065475329564933%7CUnknown%7CTWFpbGZsb3d8eyJWIjoiMC4wLjAwMDAiLCJQIjoiV2luMzIiLCJBTiI6Ik1haWwiLCJXVCI6Mn0%3D%7C3000%7C%7C%7C&sdata=5u7VAYn%2BjFAQM%2FlgkKphG71yuK74exXoJ5kueTmANXg%3D&reserved=0> 

We suggest you report in line with RECORD (CHEERS is targeted to economic evaluations of interventions and STROBE is epidemiological reporting).  Please provide the relevant completed checklist. In the checklist, please include sufficient text excerpted from the manuscript to explain how you accomplished all applicable items.
Please include the relevant associated checklist and indicate in your statement in the methods section where it can be located

The manuscript indeed followed the RECORD guidelines, and we have uploaded the RECORD table as a supplement.

DATA AVAILABLIITY STATEMENT
The Data Availability Statement (DAS) requires revision. For each data source used in your study: 
a) If the data are freely or publicly available, note this and state the location of the data: within the paper, in Supporting Information files, or in a public repository (include the DOI or accession number).
b) If the data are owned by a third party but freely available upon request, please note this and state the owner of the data set and contact information for data requests (web or email address). Note that a study author cannot be the contact person for the data.
**c) If the data are not freely available, please describe briefly the ethical, legal, or contractual restriction that prevents you from sharing it.** Please also include an appropriate contact (web or email address) for inquiries (again, this cannot be a study author).

Data Availability Statement: Data used in this paper cannot be made available due to contractual restrictions but can be purchased from Truven Health Analytics (<https://marketscan.truvenhealth.com/marketscanportal/>).

ABSTRACT
Please structure your abstract using the PLOS Medicine headings (Background, Methods and Findings, Conclusions).
Please combine the Methods and Findings sections into one section, “Methods and findings”.
Abstract Background: Provide the context of why the study is important. The final sentence should clearly state the study question.
Abstract Methods and Findings:
Please ensure that all numbers presented in the abstract are present and identical to numbers presented in the main manuscript text.
Please include the years during which the study took place, length of follow up, and clearly define the main outcome measures.
Please include the actual amounts and/or absolute risk(s) of relevant outcomes (including NNT or NNH where appropriate), not just relative risks or correlation coefficients. (Example for absolute risks: PMID: 28399126). 
Please define the numerical values contained within square parentheses (UIs as per your main methods/results).
Please also quantify the main results with p values (as well as UIs) 
Please include any important dependent variables that are adjusted for in the analyses.
In the last sentence of the Abstract Methods and Findings section, please describe the main limitation(s) of the study's methodology.

Abstract Conclusions:
Please address the study implications without overreaching what can be concluded from the data; the phrase "In this study, we observed ..." may be useful.
Please interpret the study based on the results presented in the abstract, emphasizing what is new without overstating your conclusions.
Please avoid vague statements such as "these results have major implications for policy/clinical care".  Mention only specific implications substantiated by the results.
Please avoid assertions of primacy ("We report for the first time....")

We have reviewed these requests and confirm the abstract reflects them.

AUTHOR SUMMARY
Thank you for including an author summary
Line 79: “Few studies have explored whether the combinations….” suggest “whether different combinations of conditions…” or something similar
Line 83: “16+ million…” suggest over 16 million
Line 95: “Multimorbidity adjustments should be done…” suggest performed in place of done
Please remove the data availability statement form the end of the author summary and include only in the manuscript submission form

INTRODUCTION
If there has been a systematic review of the evidence related to your study (or you have conducted one), please refer to and reference that review and indicate whether it supports the need for your study.

METHODS
Line 167: “…Strengthening the Reporting of Observational Studies in 168 Epidemiology (RECORD) guideline.” Should read, “REporting of studies Conducted using Observational Routinely collected health Data (RECORD) guideline.”
Please remove role of the funding source from the end of the methods section and include only in the manuscript submission form

FIGURES and TABLES
To improve accessibility to those with color blindness, please consider avoiding the use of green and/or red
Please provide an appropriate caption/legend for all figures and tables such that its contents are clearly described without the need to refer to the text.
Please provide a table showing the baseline characteristics of the study population
Table 1 (and throughout) where you report adjusted analyses, please also provide the unadjusted analyses for comparison, and please indicate in an appropriate caption or footnote which factors are adjusted for
Figures 1 and 2:  are not very accessible to the reader – text overlies other text and makes it unreadable.  The legend refers to sub-categories which the specific conditions presented in the graph fall into, this can be confusing to the reader.  Please revise accordingly
Figure 3: in isolation, it is difficult to interpret what this figure shows.  Is combination 1, 2, 3 (as referred to in the legend) the equivalent of single, dyads and triads (as referred to in the title)?  Please revise to improve accessibility in-line with previous comments
Figure 4: please see reviewer comments which we agree with, and revise accordingly

DISCUSSION
Please remove the sub-heading conclusion such that the discussion reads as a continuous piece of prose
Please remove statements at line 602 and 603 and include only in the masncuript submission form

SUPPORTING INFORMATION
Please provide appropriate captions for all tables
Please provide p-values where 95% UIs are reported
Please provide unadjusted analyses where relevant and in the caption/footnote indicate which factors are adjusted for
Table A1 and A2: Please define GBD 

Thank you for the suggestions, we have followed all of the suggestions except for the point on the supporting information. There are no p-values because the 95% UIs were derived using bootstrapping and simulation methods. We also cannot provide unadjusted analyses, as all regression analysis were adjusted with basic demographic covariates (we did not perform unadjusted analyses). However, since the focus of the paper is on multimorbidity, we conducted a separate regression without the disease combination interaction terms as a counterfactual for comparison, and we have added this explanation in the methods section (as also suggested by one reviewer).

Comments from the reviewers:

**Reviewer #1:** I enjoyed reading this paper, which is well-written and clearly organised. The study aims to (1) assess if disease combinations are super-additive, additive, or sub-additive in regard to health spending, and  (2) estimate disease-specific health spending that accounts for multimorbidity. The first research aim is particularly relevant to policy and clinical practice as it may inform prioritization of disease combinations most likely to result in the highest healthcare spending. Using private insurance claims of 16m enrollees, the paper finds that 60% of them (pairs or triads) are super-additive. The chronic conditions with the highest multimorbidity-adjusted spending include CKD, cirrhosis, IHD, and inflammatory bowel disease. The authors claim that prioritizing interventions to reduce the prevalence and/or spending of these conditions could yield to cost-effective results. Below I provide some comments that the authors may consider to potentially improve the paper:

1. Conceptualisation of the health expenditure model: 
*	The paper offers no rationale for the specification of the health spending equation (equation 1, page 7). How did the authors choose the independent variables? Some sort of conceptual framework, specific to multimorbidity such as the one by Zulman et al 2014 on multimorbidity interrelatedness, could have guided this piece. How do the authors think that important variables such as disease severity, time since diagnosis, or total number of conditions may be confounding existing results?

Thank you for pointing this out, we have added further details in the main text following your comment. Our study designed followed the conceptual framework proposed by Lehnert et al. (2011) *Health care utilization and costs of elderly persons with multiple chronic conditions.* They suggested that any study on multimorbidity to consider one’s use of service as a function of their predisposition to use services (predisposing factors), factors that support or impede use (enabling factors), and their need for health care (illness level), and that the latter likely has a stronger impact on healthcare use. We then reviewed our administrative claims dataset and selected variables based on availability, data quality (as reported by other studies), and the Lehnert et al. framework. For example, predisposing variables included age sex, and region of residence, but not ethnicity, religion, education, or ethnicity because they were not available in the dataset. We also lacked data on income, which is an enabling factor for whether people seek care. The reviewer’s comment on disease severity, time since diagnosis, and total number of conditions all fall under the illness factor category and they are indeed important factors. Disease severity is captured insofar as it is reflected in the diagnosis codes. The number of conditions is captured in the dyads and triads we included in the models. We were unable to capture data on time since diagnosis since this is a cross sectional study, and we have highlighted this as a limitation in the paper (and this is a common limitation in the broader literature).

Reference: Lehnert T, Heider D, Leicht H, Heinrich S, Corrieri S, Luppa M, Riedel-Heller S, König HH. Health care utilization and costs of elderly persons with multiple chronic conditions. Medical Care Research and Review. 2011 Aug;68(4):387-420.

*	Why did the authors limit themselves to disease dyads and triads? Based on the characteristics of the study population this may be particularly relevant (94% of those with multimorbidity had more than three conditions). Did the authors at some point consider the possibility of characterising the actual disease combinations in the sample using cluster analysis?

Going beyond dyads and triads was something we considered initially in the study design. However, we chose not to do so due to two reasons: first, it was computationally much more challenging to go beyond a 3-disease combination. Just with dyads and triads, we had 41,664 combinations (all unique combinations of 63 conditions). If extended to tetrads, we would have several million more covariates, making the regression very difficult to run, even under the SGD model. Second, we noticed that even among the triads, the frequency (prevalence) of most of the combination were low. While multimorbidity is common in this population, the combinations were concentrated to a few combinations, and the remainders were rare. The higher prevalence combinations are presented in Appendix Table A6. We did not perform a formal cluster analysis but rather included all possible combinations as covariates in our regression model.

2. Estimation of the health expenditure model:
*	Which estimator was used in the stochastic gradient descent approach? How does this estimation approach account for the skewed (non-normal) distribution of health expenditures?

The estimator is the same as the OLS. The stochastic gradient descent follows the OLS equation but takes an iterative approach to optimize the same objective function as a regression model. It randomly selects a subset of the data and iteratively approximates the estimator. Spending was transformed on a natural logarithmic scale. This has been clarified in the main text.

*	How was goodness of fit of the model assessed? What percentage of the variability in individual health expenditures is this model able to explain?

The underlying approach of SGD is very close to regression models, and the model outputs include goodness of fit metrics, such as MSE and R^2^. In the appendix (and copied here) we provide a summary of the R^2^ of the SGD model runs:

|  | Mean | Median | Range | SD |
| --- | --- | --- | --- | --- |
| R2 | 0.320 | 0.324 | 0.165 – 0.417 | 0.058 |

3. Limitations of the sample used:
*	The authors used a cross-sectional sample. Without adjusting for time since diagnosis or disease severity, the disease stage that the estimated expenditures pertain to is unclear.

*	How is the study sample different from a US nationally representative sample? On page 5, the authors state that differences have not been assessed before but I wonder if they could be at least approximated by comparisons to publicly available sources. How could the disease prevalence of a subsample of commercially insured enrolled differ from a nationally representative sample? I think this is an important point to understand the usability and generalisability of study findings.

We agree with the reviewer about the limitation of using a cross-sectional dataset. We indeed only have data on spending associated with a diagnosis and cannot adjust time since diagnosis or severity. Our estimates represent the average all patients who had the disease regardless of their severity. This has been stated in our limitations more clearly. On the question of our sample compared to the US population, there are several key differences. First is age, we only include adults below age 65. Second, other studies have shown that in Marketscan the South region is over represented (44% in this dataset compared to 30%, as reported by Dunn et al. (2013)). Third, we do not have income data, and it is likely that our study population has higher income than the nationally representative sample because this is an employment-based (and from larger employers) claims database. For example, breast cancer is more likely to occur among higher income women, and in our study the prevalence rate for breast cancer is much higher than the national average. Similarly, we noted a much lower prevalence rate of substance abuse in our dataset. We considered providing a table comparing the prevalence rates from the Global Burden of Disease dataset to our study, but noted that the age groups (ages 20-54 versus 18-64) and disease categories are not directly comparable.

Reference: Dunn A, Liebman E, Pack S, Shapiro AH. Medical Care Price Indexes for Patients with Employer-Provided Insurance: Nationally Representative Estimates from MarketScan Data. Health Serv Res. 2013 Jun;48(3):1173–90

4. Consideration on study definitions: What is the rationale behind not restricting claims to primary diagnosis to generate a more accurate estimate of the cost of a chronic condition? (page 6, lines 205-206). Does that mean that under the cost of CKD, costs of acute events are also included?

This is a point we debated during study design. The focus of this study is multimorbidity, and by only focusing on primary diagnosis we will be answering a different research question (i.e., spending of each condition but not how the combinations affect spending). The reviewer is correct that any spending associated with the patient will be assigned to all diagnoses one has. From a multimorbidity perspective, we are not sure whether spending is tied to specific conditions or whether it is caused by the combination of diseases, for example, interactions between diseases and whether the spending is sub-additive, additive, or super-additive. In other words, a patient with CKD may have other healthcare encounters and spending that may not be directly related to CKD but may be due to the interaction of CKD and other co-existing conditions.

5. Other conceptualisation issues:
*In my opinion, how generating multimorbidity-adjusted health expenditure estimates for *individual* chronic conditions helps understanding the economic burden of multimorbidity could be more clearly articulated. The paper seems focused on multimorbidity, yet its conclusions apply to individual conditions. How do the conclusions of this paper then relate to the design of interventions to improve health outcomes of individuals with multimorbidity?

This is a useful suggestion and we have reflected it in the revised manuscript. The focus is still on multimorbidity since we apply multimorbidity-adjustments to single condition spending estimates. We believe both perspectives are important for the following reasons. First, discussions around the multimorbidity-adjusted single condition estimates are needed because single conditions are still at the center of the current research, policy, and clinical frameworks. Second, much more emphasis should be given to disease combinations. We provided estimates for the disease combinations and described the differences between super-additive, additive, and sub-additive spending relationships. Understanding why certain combinations are super- or sub-additive could be key to understanding how these diseases and its care interact with one another. In the conclusion section, we added that both single conditions with high prevalence and spending, as well as super-additive disease combinations should be the priority.

*	The authors only considered costs but not health outcomes, so the conclusion on cost-effectiveness towards the end is hard to follow and unclear how it can be drawn from the study results.

Our study looked at both prevalence and spending, and the point we made refers to the fact that if certain high-spending and high-prevalence diseases are prevented, it could lead to large spending saving with large reduction in prevalence. What we do not have in our study is the cost of interventions (such as prevention or treatment policies or services). We follow the reviewer’s comment and agree that “cost-effectiveness” may not be the appropriate terminology since we do not have intervention costs. We have revised them accordingly to say that policymakers interested in reducing overall health spending could focus on these priority diseases.

Other comments:
*	From what perspective was annual spending computed? It seems to include provider payments but exclude out-of-pocket expenditures incurred by the beneficiary? What is the rationale behind this choice and how does that affect your disease-specific expenditure estimates? How could spending data be negative (line 184, page 6)?

As administrative claims data are primarily designed for reimbursement purposes, we took a healthcare payer’s perspective. This likely underestimates the true costs, since this excludes any out-of-pocket payments and indirect costs (such as reduced productivity) incurred by the patients. We have added this explanation in the main text. Negative spending rarely occurred in our dataset (less than 100 individuals with small dollar amounts), and this is likely due to correcting for overbilled line items from previous years.

**Reviewer #2:** Reviewer's comments

1.	Summary of the research and general comment:

This is an interesting and topical multimorbidity study, which estimated the costs of disease combinations and explored the interaction of co-existing conditions within an individual and their impact on costs. The study is very well structured, with a large sample size and number of disease combinations. 

The main limitations are that the study did not capture cost components beyond outpatient and inpatient services and unable to account for changes through time; however these are well acknowledged in the limitation. The methods section relating to the decomposition of the change in multimorbidity-adjusted spending can be more elaborative.

In general, writing up a paper on the topic of (the cost of) multimorbidity is challenging. It is easy for readers to get lost and confuse themselves amidst the myriads of jargons, the overwhelming number of ways diseases are combined, and all the different ways of expressing the same term (e.g. when referring to the cost of a condition, it is important to emphasize whether the author is referring to a condition as part of a disease combination or a single condition by itself; etc.)

In light of such complexities, phrasing should be as accurate and consistent as possible and details on methodology, terminologies should be elaborated as much as possible, to enable readers (who are not experts on multimorbidity) to easily follow. It is commendable that the author gave various examples of specific conditions/dyads throughout the paper. It would be helpful to be a bit more elaborative in places (see points below), and to be consistent with the choice of terms. 

Thank you for the positive and constructive feedback. We agree that the topic can be complicated and therefore there is a need to be as clear, consistent, and accurate as possible. We have updated the manuscript with these goals in mind.

2.	Discussion of specific areas for improvement:

A.	Major comments:
1.	Line 190-192: Can the author explain why injuries were excluded from the list of chronic conditions? For example, hip fracture rates in the US are amongst the highest in the world and links to long-term disability outcomes, similar to stroke.

We agree with the reviewer that some injuries lead to long-term disabilities and have added this as a limitation. We chose not to include injuries for four reasons. First, our GBD experts were asked to choose GBD causes that were deemed chronic, defined as having a duration of one or more years. For all causes, they were asked to think “on average” – for example, breast cancer is “chronic” for all patients; respiratory infections are “on average” not (i.e., some infections may last over a year, but most will not). In the case of injuries, the experts deemed that they were not chronic (only a relatively small fraction of the time). The full list of GBD injury causes are listed here: <https://vizhub.healthdata.org/gbd-results/>.

Second, our team has previously worked on ICD codes for injuries in other projects, and attributing spending to injuries requires a much more complicated process than non-injuries. For example, injuries can be coded in the ICD system in one of two ways: by the nature of injury (N-codes) or by the external cause of injury (E-codes). The external cause of an injury might be a car crash, while the nature of that injury might be a broken hip. The former are most relevant for clinicians and for billing purposes, while the latter are more relevant for public health and describe what the injury was but less frequently included in administrative claims. Injuries in the GBD hierarchy correspond to injuries classified by E-codes. In the cases where E-codes are not available, we probabilistically map N codes to E codes based on proportions in the data source that require both. This extra step means our injury estimates are a little less certain than other causes of illness. After a deliberate discussion, we concluded that including injuries may distract the readers from the other diseases and results. Third, our dataset includes patients ages 18 to 64, and while injuries with long-term disabilities do occur, they are proportionally small compared to other chronic conditions. Finally, we also noticed that in the existing multimorbidity literature, injuries are not included as a condition or disease.

2.	Line 301-305, the author described the third and fourth step in estimating spending associated with each individual health condition, adjusting for multimorbidity. It will be beneficial to the readers if the author explains the reasons for undertaking these steps. Perhaps, the author can also demonstrate this using an equation. 

Thank you for the suggestion. We have edited this section as follows: “Third, we calculated prevalence, i.e., the probability of the disease combinations occurring among people with diabetes (for example, the probability of someone with diabetes also having osteoarthritis). This is needed for the next step, in which we adjust each spending estimates based on its prevalence, such that more common combinations of diabetes and another disease receives a higher weight than combinations that are less common. This is done by multiplying the spending associated with the combination with its prevalence from step 3, and summed across disease combinations. This final figure is the part of the spending associated with dyads that should be attributed to diabetes."

3.	Can the author elaborate more in the method section what they specifically did to arrive at the estimates for the total spending associated with combinations?

Following equation 1, the terms ${dyad}_{k}$ and ${triad}_{l}$ represent the interaction terms for all possible chronic condition dyads and triads, and these coefficients are the estimated spending associated with the combinations (above and beyond the sum of the individual diseases). For example, to estimate total spending associated with diabetes and osteoarthritis, we add up the coefficient on diabetes, coefficient on osteoarthritis, and coefficient on the interaction term diabetes * osteoarthritis. We have added this clarification on page 8: “Estimates of total spending associated with any combination can be derived by adding the coefficients for the single conditions independently and the coefficients from the interaction terms (from the combinations). For example, total spending for diabetes + osteoarthritis is the sum of the coefficient from diabetes, coefficient from osteoarthritis, and coefficient on the interaction term diabetes * osteoarthritis.”

4.	Did the author log-transform cost, or how did the author address non-linearity/non-normality/unequal variance in the data?

Spending was transformed on a natural logarithmic scale. This has been clarified in the main text.

5.	Line 420-427: "Decomposition of the change in multimorbidity-adjusted spending by the type of combination (single, dyads, and triads) for conditions with the highest spending per treated case". To the reviewer's understanding, this is the spending on single conditions, when taking into account other co-morbidities. For example in the case of CKD, is the below the correct interpretation of this finding?
*For MM-adjusted spending for CKD, less than 25% is attributed to the base cost itself (in the case of an individual with only 1 condition, i.e. CKD). The yellow stripe (which represents around 50% of the total), is the increase in cost of CKD due to the interaction with the second condition -  in the case of dyad. The red (which represents over 25% of the total), is the increase in cost of CKD due to the interaction with a third condition - in the case of triad.
*Does it imply that individuals with only 1 condition (CKD only) will incur lower cost than the adjusted estimate above and proportionate to the green stripe in the graph?
*Is this the increase 'on average'? For example, if CKD is comorbid with 2 other conditions (triad), the increase in CKD cost is the same on average and not particular to any specific conditions?
The reviewer advises that the author describes more in this section, to help readers understand what is being explored. If the above bullet points are the correct way to interpret this result, perhaps the author can consider elaborating it in such a way - citing the specific breakdowns (25%, 50%,…) as an example.

Yes, the reviewer is correct that this paragraph and accompanying figure (Figure 2) take the single-condition perspective, i.e., spending associated with a single condition after multimorbidity-adjustment. Your interpretations on the decomposition and the colored bars are correct. The increases are not exactly “on average” but instead weighted average – average across all the possible dyads that CKD could be with, weighted by the prevalence of the dyads. A further decomposition is presented in Figure 3, in which the contribution of the other conditions (in combination with CKD, for example) are shown. We have followed the reviewer’s suggestion and expanded these paragraphs to provide further interpretation of the figures.

6.	Line 429: "Comparing pre- and post-multimorbidity adjustment spending estimates, we found that 50 conditions (among 63) had higher spending after adjustments, 7 had less than 5% difference, and 6 had lower spending (Table 1)." The author mentions pre-adjusted costs but has not presented these anywhere, or how it was calculated. The reviewer deems this necessary, especially for the latter (to be included in the method section). 

Thank you for pointing this out. We have added a paragraph in the methods describing how the unadjusted costs were derived (namely, using the same regression framework without the dyad and triad interaction terms). This is used simply for comparison against the main results, as have been clarified now in the methods and results sections.

7.	Line 442: "In other words, among these diseases, estimated multimorbidity-adjusted spending are at least twice higher than the non-adjusted spending estimates." By "non-adjusted", is the author referring to the average cost of this specific condition among those with only 1 condition (base cost)? By "twice higher", does the author mean "double"? The reviewer suggests that the author rephrases "twice higher" to "double" for more accuracy and clarity. The author may also consider revising Line 442-443 as follow, so as to clearly distinguish between the spending for individual conditions and the spending for combinations. "In other words, the estimated multimorbidity-adjusted spending for these individual diseases are at least, double that of their respective non-adjusted estimates." 

We agree that this paragraph as it was written was not easy to read. We have edited it, first by linking it to the previous paragraph (decomposition in Figure 2), and removing the descriptions on the multiples. How we described it previously was in fact incorrect. For example for CKD, spending is not 3x or 4x the unadjusted estimate; rather, CKD’s multimorbidity-adjusted spending is 3x or 4x than the spending for only CKD. We have decided to remove this description to avoid confusion.

9.	Line 438-448:
For this section, the reviewer would also like to clarify for enhanced understanding:
*In the example of CKD, the different colors show the increase in cost for CKD when CKD is comorbid with other conditions. For example, when comorbid with "other NCD" (bright green), the cost of CKD doubles that of the base cost (the base cost is the cost of CKD in the case of an individual with only 1 condition, i.e. CKD). Is this the correct way to understand?
*Is the change in CKD cost different when it is comorbid with 2 other conditions simultaneously instead of 1 (in the case of triads)? For example, when CKD is comorbid with both CVD and 'other NCD' at the same time, is the increase in cost of CKD simply additive of the thickness of their respective stripes?

Thank you for these questions, as they help us interpret our results better for the readers. You are partly correct in that spending for CKD increases with dyads and triads. However, your interpretation can be applied to (the new) Figure 2 and not Figure 3. In Figure 2, we decomposed the dyads and triads so we know how much increase can be attributed to dyads or triads. In Figure 3, however, we combined both dyads and triads and instead analyzed the data using the type of conditions. For example, the bright green “other NCD” bar represents the increase in CKD spending associated with any types of combination with other NCD, and this could be either dyads or triads (i.e., CKD + other NCD + another disease). Also note that these are all prevalence-weighted, so they reflect how common/uncommon CKD is in combination with other diseases.

10.	Line 446: "For cancers, we see that other cancers account for the largest share of increases, and specifically for brain and nervous system cancer we also see a large contribution from neurological disorders." 

- By 'other cancers', the reviewer assumes the author is referring to 'neoplasms' in Figure 3, however 'neoplasms' may be benign (not cancer) or malignant (cancer). The author should refrain from using the term 'other cancers'.

Thank you for pointing this out, we have edited all wordings accordingly.

11.	It would be useful if the author also presented in the Appendix the estimated spending for all disease combinations with a prevalence rate greater than 100 per 100,000 (or a higher threshold, in case the number of combinations is too high).

This is currently presented as Appendix Table A6.

12.	The estimated spending for disease combinations reported in the paper are on average much lower than those reported in other studies from the US (see 10.1186/s12916-022-02427-9), some of which also analyzed MarketScan data. Does the author have more thoughts on why that is?

The paper referred by the reviewer performed a meta-analysis across 15 studies with large differences in study population and setting. Most of the papers come from target populations, such as those with Medicare and/or Medicaid eligibility (for example, Lin et al. 2010), older adults (MacNeil-Vroomen et al. 2020), or veterans. Another potential reason is that several of these studies looked at patients with greater severity.

Among those that are more comparable, such as studies using Medical Expenditure Panel Survey (though most include all ages 18 and above and not just working age population), the results are more comparable qualitatively, although they only report incremental health spending associated with a condition and not after adjusting for multimorbidity. For example, our multimorbidity-adjusted spending for stroke is about $5400, compared to Lekoubou et al. (2018)’s $4057. Kirkland et al. (2018) and Wang et al. (2017) estimated that hypertension adds an additional $2000 when in combination with others. We estimated the multimorbidity-adjusted spending at about $550.

There were four papers that analyzed Marketscan data in the review. Given the differences in research question, methods, time period, and age groups, the numbers are not directly comparable. Li et al (2019) reported incremental increase in rheumatoid arthritis to be $578 (versus our estimate of $424). Mannino et al. (2015) studied older COPD patients (40 to 90 years old) and co-morbidities, and reported that having chronic kidney disease and anemia to have the highest overall healthcare cost. Here the numbers are quite different, for example, they estimated having CKD to incur additional $40,000 in spending, while we estimate it to be less than $20,000. Rustgi et al. (2020) estimated the incremental cost of having chronic kidney disease among chronic liver disease patients of ages 18 and above (including older population), and reported an average additional $4000 annually. Our estimate is around $2500. Mehta et al. (2018) estimated that people ages 18 and above with diabetes and previous history of cardiovascular diseases had approximately $2000 more spending than those without the history. In our study, the coefficients for the interaction terms between diabetes and CVD (such as ischemic heart disease, stroke, atrial fibrillation) were approximately $500. Adding the coefficient for the CVD itself and the interaction term will amount to approximately $2500-4000, depending on the disease.

We have cited the relevant papers listed here in the main text.

Reference:

MacNeil-Vroomen JL, et al. Health-care use and cost for multimorbid persons with dementia in the National Health and Aging Trends Study. Alzheimer’s Dement. 2020;16(9):1224–33.

Lekoubou A, Bishu KG, Ovbiagele B. Nationwide healthcare expendi- tures among hypertensive individuals with stroke: 2003-2014. J Stroke Cerebrovasc Dis. 2018;27(7):1760–9.

Wang G, et al. Annual total medical expenditures associated with hypertension by diabetes status in U.S. adults. Am J Prev Med. 2017;53(6):S182–9.

Li N, Chan E, Peterson S. The economic burden of depression among adults with rheumatoid arthritis in the United States. J Med Econ. 2019;22(4):372–8.

Mannino DM, et al. Economic burden of COPD in the presence of comorbidities. Chest. 2015;148(1):138–50.

Rustgi VK, et al. Health care resource use and cost burden of chronic kidney disease in patients with chronic liver disease: a real-world claims analysis. Hepatol Commun. 2020;4(10):1404–18.

Mehta S, et al. Differences in all-cause health care utilization and costs in a type 2 diabetes mellitus population with and without a history of cadiovascular disease. J Managed Care Specialty Pharm. 2018;24(3):280

13.	Exploring the distribution of cost components (i.e., outpatient vs inpatient) of costly disease combinations would also be useful in identifying the driver of high costs. This is particularly relevant in the discussion, where the author discusses the potential reasons for super-additive spending.

This is a good follow up study to consider for the future. We have added this suggestion in the discussion section.

14.	The strengthening of the integrated primary care system is an important intervention to target as the burden of multimorbidity increases. It would be relevant to mention this alongside prioritizing prevention, in the Author Summary (Line 94-99). The author has mentioned service coordination, patient-provider communication in the discussion, and these are aspects of integrated care. In addition, provider-provider communication and self-management are important aspects to also consider. Provider financing models (fee-for-service vs capitation) may also affect the level of service utilization/cost. 

15.	Line 481-483: Here it would be useful to give an example of triads or dyads that had sub-additive spending.


B.	Minor comments:

1.	Line 167-168: Please cite the RECORD guideline.

2.	Line 198: Please remove "to".

3.	The "==" in equation 3 should just be "=".

4.	Line 377-380: Are the reported costs in brackets total or incremental? If they are indeed incremental (i.e. the overall increase in cost due to the interaction of the component diseases), then perhaps the author should clearly specify that, and also add a "+" before each Dollar sign.

E.g. blood cancers + hemoglobinopathies and hemolytic anemias (henceforth anemias) (+$3227, 95%UI [2541-3905]).

5.	Line 382: Please add '$' to "cirrhosis + hyperlipidemia (-610 [-838- -370])" for consistency.

6.	Line 384: Please revise '$-' to '-$' in "hyperlipidemia ($-545 [-727- -363])" for consistency.

7.	Line 422: Consider changing "size" to "sizes".

8.	Line 473: Please revise "point to" to "have pointed to".

9.	The reviewer finds the style of visualization in Figure 1 a bit difficult to follow.

10.	For Figure 4, please name the ten graphs from A to J for ease of referencing.

We are impressed by the level of detail in this review, and we are grateful for your suggestions. We have carefully addressed all of them.

11.	Figure 4:

*	Each graph gives the false impression that it represents the proportions of cost of various conditions co-existing in the same individual. Readers may have to refer to the main text to understand. In theory, graphs and charts should be self-explanatory; readers should be able to infer from them immediately without having to read the main text. If this is the most optimal way to visualize the result, the author may want to be more descriptive in the main text (as suggested in the points made above) as well as add a short sentence on what is going on in the graphs/what was done to get there (also for Figure 3).

*	The bright red and/or dark orange stripe are often small and shadowed by the dark red bar (due to the color palette, and as these stripes tend to be very thin), the author may consider changing the bright red/dark orange to other distinctive colors or change the orders of the colors so that distinctive colors are next to each other. In the legend, dark orange refers to both diabetes and CKD merged together. In the first graph for CKD, is it correct that there is a thin dark orange stripe that represents both Diabetes and CKD? If yes, would there be an overlap here? If that stripe is actually not dark orange, but a bright red stripe (these two colors cannot be distinguished by the reviewer, as it is thin and shadowed by the dark red color of CVD), then is Diabetes missing from this graph? Where in fact, does the Diabetes and CKD stripe (dark orange) appear across the 10 graphs?

Thank you for the suggestion. We have expanded the interpretation of this figure to reflect your comments. Namely, we used CKD as an example to illustrate what each bar and color mean. On the specific question of diabetes, diabetes contributed less than 1% to the increase in CKD spending. One possibility is that the relationship between CKD (or other diseases) and diabetes is additive, i.e., the interaction term is close to zero because their spending is already captured by the coefficient of each term. By definition, these colored bars only show prevalence-weighted spending of super-additive or sub-additive interactions, i.e., spending that is above and beyond (or lower) the sum of the spending of single conditions. Another possible reason is that estimated spending for diabetes is relatively lower than most other diseases ($910, see Table 2).

**Reviewer #3:** This study explores the relationship between multimorbidity and spending on inpatient and outpatient care among adults in the US.

Comments:

"This study is reported following the Strengthening the Reporting of Observational Studies in 168 Epidemiology (RECORD) guideline."
Can the authors please clarify if they followed STROBE or RECORD guidelines here?
Can the authors also please supply the associated checklist in the supplementary material?

"This study took a person-based regression approach - regressing a person's total 2018 health spending on health conditions indicators - in estimating spending per treated case [28,29]. We applied the following linear regression model:... "
and
"we applied a regression framework using the stochastic gradient descent (SGD) approach...  we applied a lasso penalized regression model to shrink the coefficient values of these covariates"
and
"To ensure stability of the model results, we conducted 50 SGD model runs and bootstrapped the results across runs for 10,000 times to get the estimates for all coefficients"
The authors have applied technically appropriate methods, which they describe clearly within the article.

Similarly, the authors have conducted rigorous and comprehensive models for "Estimating spending associated with each individual health condition, adjusting for multimorbidity".

"First, to generate 95% uncertainty interval (UI) for spending associated with disease combinations, we bootstrapped the means from all the model runs for 10,000 times. Second, to generate UI for spending associated with single conditions, we ran Monte Carlo simulations (n=1,000 draws) while varying the estimates associated with the combination and the proportion of combination attributed to each single condition." 
The authors have conducted suitable analyses to help demonstrate the uncertainty and robustness of the study findings.

Overall, the authors have communicated the study Results accurately and the main study limitations have been suitably addressed in the Discussion.
Furthermore, the authors provide satisfactorily detailed methods and results in the supplementary material.

We appreciate the positive feedback and the detailed review. We followed the RECORD guideline and have added it as a reference in the manuscript, and have attached the checklist as a supplementary material.
